# Development, content and planned evaluation of a behavioural support intervention to reduce ultraprocessed food intake and increase physical activity in UK healthcare workers: UPDATE trial stage 2 study protocol

**DOI:** 10.1136/bmjopen-2025-107435

**Published:** 2025-10-29

**Authors:** Gabriella Niamh Heuchan, Caroline Buck, Rana Conway, Samuel Dicken, Adrian Carl Brown, Friedrich C Jassil, Janine Makaronidis, Chris Van Tulleken, Claudia Angela Gandini Wheeler-Kingshott, Rachel Batterham, Abigail Fisher

**Affiliations:** 1 Behavioural Science and Health, UCL, London, UK; 2 Centre for Obesity Research, Division of Medicine, UCL, London, UK; 3 Biomedical Research Centre, UCLH, London, UK; 4 Bariatric Centre for Weight Management and Metabolic Surgery, University College London Hospitals NHS Foundation Trust, London, UK; 5 Service of Endocrinology and Diabetology, Department of Medicine, Geneva University Hospitals, Geneva, Switzerland; 6 Division of Infection, University College London, London, UK; 7 Neuroinflammation, Queen Square Multiple Sclerosis Centre, UCL Institute of Neurology, London, UK; 8 Department of Brain and Behavioural Sciences, University of Pavia, Pavia, Italy; 9 Digital Neuroscience Center, IRCCS Mondino, Pavia, Italy

**Keywords:** NUTRITION & DIETETICS, Obesity, Behavior, Psychosocial Intervention

## Abstract

**Introduction:**

Diets high in ultraprocessed food (UPF) are associated with poor health outcomes and weight gain. Healthcare workers are particularly at risk of consuming diets high in UPF due to erratic work patterns, high stress and limited access to fresh food at work. Despite this, no interventions to date have specifically targeted a reduction in UPF intake in healthcare workers.

**Methods and analysis:**

This article describes the development and content of a 6-month behavioural support intervention targeting a reduction in UPF intake in UK healthcare workers. The intervention was offered to all participants who took part in the UltraProcessed versus minimally processed Diets following UK dietAry guidance on healTh outcomEs trial—a two-stage study in which Stage 1 was a controlled-feeding crossover randomised controlled trial of provided UPF versus minimally processed food (MPF) diets (published previously) and was completed before the start of Stage 2. Stage 2, reported here, aimed to support participants to reduce their UPF consumption, increase MPF and increase physical activity in real-world settings. The intervention was developed using the behaviour change wheel framework, which systematically links behavioural diagnoses to intervention functions, incorporating the capability, opportunity and motivation model for behaviour change. It included tailored one-to-one and group support sessions, bespoke digital and print resources and a mobile-optimised website. The detailed description is intended to support future replication and adaptation. The acceptability and feasibility of the intervention will be assessed using quantitative and qualitative data in a future paper.

**Ethics and dissemination:**

Sheffield Research Ethics Committee approved the trial (22/YH/0281). Findings will be disseminated through peer-reviewed publications, conference presentations and summaries shared with participants and stakeholders.

**Trial registration number:**

NCT05627570.

**Strengths and limitations of this study**

- A key strength of this behavioural support programme is that it was developed in line with evidence-based behaviour change techniques and intervention functions.
- A strength of this behavioural support programme is the in-depth quantitative data collected at baseline and after the intervention to assess self-reported beliefs and attitudes across several domains of diet, exercise and lifestyle.
- A limitation of this work is that the study was powered for the Stage 1 crossover controlled trial, and the behavioural support programme was offered to all in a pre–post exploratory design; therefore, efficacy cannot be determined.

## Introduction

 Almost two-thirds of adults in England live with overweight or obesity,^1^ raising the risk of life-limiting diseases and an early death.^2^ Obesity poses a significant healthcare challenge, with financial implications estimated at £58–63 billion in the UK.^3 4^ Large numbers of UK healthcare workers are living with obesity, including 25% of nurses and 24% of those in non-health-related hospital roles.^5^

Changes in the food environment and the wide availability of ultraprocessed foods (UPFs) have been identified as potential contributors to obesity.^6 7^ UPFs are defined by the Nova classification system as food formulations generally comprising five or more ingredients, binding molecules, including preservatives, flavourings and colourings to the extracts of original foodstuffs.^8^ Examples include most commercial breakfast cereals and breads, convenience foods and confectionery.^8^ The relatively low cost of UPF^9^ coupled with widespread availability^10^ and convenience,^11^ among other factors, has contributed to the high levels of consumption in the UK, where UPF accounts for an estimated 60% of adults’ daily energy intake.^12^ In 2019, a randomised controlled trial (RCT) of ad libitum UPF versus minimally processed food (MPF) diets matched for presented energy, macronutrients and participant-rated pleasantness found people consumed approximately 500 kcals/day more on the UPF diet than the MPF diet.^7^ During the 2-week UPF diet, participants also gained nearly a kilogram of bodyweight compared with nearly a kilogram weight loss on the 2-week MPF diet. A recent crossover RCT in fewer participants (n=9) comparing 1-week ad libitum UPF and MPF diets matched for presented energy, macronutrients and energy density similarly found participants consumed 813 kcal/day more on the UPF diet than the MPF diet, gaining 1.1 kg more weight.^13^ Several prospective cohort studies have also shown that higher consumption of UPF is associated with increased risks of overweight and obesity,^14 15^ cancer,^16^ cardiometabolic disease,^17^ poorer mental health^18^ and all-cause mortality.^19^

With mounting evidence linking high UPF intake to poor health outcomes, there is a need for interventions to support people in reducing their intake; however, until recently, relatively few had been developed. Encouragingly, there has been a notable increase in the past 3–4 years, with research now emerging across diverse populations and settings. Recent behavioural intervention trials targeting UPF intake have been conducted in Brazil among pregnant women,^20 21^ adolescents^22 23^ and individuals diagnosed with metabolic syndrome.^24^ In the USA, pilot pre–post studies have targeted adults seeking behavioural treatment to improve dietary habits^25^ and food pantry clients (individuals experiencing food insecurity), among whom the intervention aimed to reduce UPF availability and consumption.^26^ All interventions featured group or individual sessions with a trained professional, with varying additional components, such as motivational approaches based on the transtheoretical model of change,^23 24^ goal-setting activities^23 24 26^ and provision of print materials.^21 26^ Some, but not all, reported effective reductions in UPF intake.^2022^,^25^

Beyond these trials, evidence from other interventions shows that reducing UPF intake can elicit meaningful metabolic and dietary improvements, even in the absence of weight change. In one study, reported outcomes include reductions in visceral fat and increases in lean mass percentage, alongside improved glycaemic markers and lipid profiles, resulting in pre-diabetes remission in half of the participants and metabolic syndrome remission in 70%.^27^ Other interventions designed to reduce UPF intake have demonstrated improvements in dietary quality, such as increases in moderately processed food intake and fruit consumption,^28^ and a rise in fruit and vegetable intake among children.^29^

These studies provide promising evidence that behavioural interventions to reduce UPF can be effective; however, to date, no interventions have been conducted in the UK nor have any targeted healthcare workers.^25^ Healthcare workers may be at particular risk of having a higher UPF intake due to factors, including high availability of unhealthy food in the hospital environment, erratic shift work patterns, irregular breaks and work-related-stress-induced eating.^30^ Healthcare workers may require tailored support to overcome the multiple barriers they face; therefore, well-designed behavioural interventions targeting this group are required. Specifically, interventions should be based on evidence-based behaviour change models, such as the capability, opportunity and motivation (COM-B) model.^31^ The behaviour change wheel (BCW) for designing behaviour change interventions has the COM-B model at its centre and is highlighted and described in the UK National Institute for Health and Care Excellence public health guideline on behaviour change^32^ and Public Health England’s local^33^ and national^34^ government guidance for achieving behaviour change.

Investigating the effects of Ultra-Processed versus minimally processed Diets following UK dietAry guidance on healTh outcomEs (UPDATE) is a two-stage study on UPF consumption among healthcare workers in the UK.^35^ Stage 1 was a 2×2 crossover RCT comparing the health effects of MPF and UPF diets adhering to UK dietary guidelines set out in the Eatwell Guide.^36^ It included a specified washout period between the two diets as a part of the crossover design, which was relevant only to Stage 1 analysis. All participants completed Stage 1, including the washout period, before progressing to Stage 2.

Stage 2 of UPDATE was a 6-month behavioural support programme to reduce UPF intake and increase physical activity and was independent of the Stage 1 crossover design. The UPDATE trial protocol paper was published previously,^35^ with a focus on Stage 1 and highlighted that we would produce a follow-up describing details of the behavioural support intervention development and content to facilitate transparency and future replication. Lack of transparency in reporting of design and content of behaviour change interventions is an issue that has been previously documented.^37^ Here, we describe the stages of planning and design of the intervention, as well as the intervention content in detail.

### Objectives

The overarching aim of Stage 2 of the UPDATE trial was to develop a behaviour change intervention to support healthcare workers to reduce UPF intake (and increase physical activity) and explore the feasibility and acceptability of the intervention. The objectives of this article are as follows.

1. Describe the development and content of the UPDATE behavioural support programme.
2. Describe the planned exploratory analyses and process evaluation.

## Methods and analysis

### Sample and setting

Recruitment, inclusion and exclusion criteria are described in full in the main trial paper.^35^ In brief, participants were 55 staff (≥18 years old) recruited from one hospital trust in Central London, UK, who were living with overweight or obesity (BMI≥25 to <40 kg/m^2^) and had habitual UPF intake of ≥50% of total energy intake.

### Intervention development

Current and planned UPDATE behavioural support work from development to evaluation follows the Medical Research Council (MRC) framework for developing and evaluating complex interventions.^38^ The UPDATE behavioural support programme was developed using the BCW framework for intervention development,^31^ which is designed to support the early stages outlined in the MRC framework. Key steps in the BCW guidance include using the COM-B^1^ to (1) understand behaviour and link COM-B to the theoretical domains framework (TDF) to use behaviour change theory, (2) identify intervention options (intervention functions) and (3) identify content by selecting behaviour change techniques (BCTs).^31^ Content was selected considering affordability, practicability and cost-effectiveness.^39^

At the time of development, there was no literature on UPF reduction in healthcare workers, so to understand the behaviour and what needed to change, existing literature on barriers and facilitators to healthy eating in healthcare workers was extensively reviewed, as we hypothesised that influences would be similar. Identified barriers included work stress,^30 40^ abnormal eating patterns due to shift work and irregular breaks,^3040^,^42^ limited self-efficacy,^30 40^ high availability of unhealthy food and limited access to healthy food at work,^30 40^ including being gifted unhealthy food by patients.^30 40 41^ Facilitators to healthy eating included peer support,^30 40^ self-monitoring^30^ and optimism around achieving goals.^30^ Barriers and facilitators were mapped onto the COM-B and TDF (tables1  2). We also gathered COM-B and TDF linked barriers and facilitators from our participants in an introductory one-to-one session (described in more detail in the next section) before they began the behavioural intervention.

**Table 1:** Key domains identified as potential barriers to the context of reducing UPF intake in healthcare professionals aligned with elements from the COM-B model

COM-B	TDF	Description
Psychological capability	Knowledge	Lack of information on what UPFs are and how to recognise them.
	Behavioural regulation	Lack of strategies to support goal setting/action planning/self-monitoring in a healthy eating context.
	Memory, attention and decision processes	The ability to retain information about UPF. Remembering to buy ingredients, organisation and ability to plan.
Physical capability	Physical skills	Lack of cooking proficiency and ability.
Social opportunity	Social influence	The influence of others on eating habits.
Physical opportunity	Environment	Lack of opportunity to access MPF easily and high availability of UPF.
Reflective motivation	Beliefs about consequences	Unfamiliarity with links between UPF and health.

COM-B, capability, opportunity and motivation model for behaviour change; MPF, minimally processed food; TDF, theoretical domains framework; UPF, ultraprocessed food.

**Table 2:** Key domains identified as potential facilitators to context of reducing UPF intake in healthcare professionals aligned with elements from the COM-B model

COM-B	TDF	Description
Physical capability	Physical skills	Skills to prepare meals using minimally processed, healthy ingredients.
Psychological capability	Knowledge	Understanding the impact of UPF, knowledge of the importance of making changes, ability to make changes, knowledge about cooking and purchasing MPF and reducing UPF.
	Memory, attention and decision processes	Memory, attention and capacity to plan changes.
Social opportunity	Social influences	Cultural beliefs and social support align with reducing UPF.
Physical opportunity	Environmental context and resources	Access to and finances for MPF.
Reflective motivation	Social/professional role and identity	Identify with a healthier lifestyle.
	Beliefs about consequences	Belief that managing dietary behaviours is important.
Automatic motivation	Reinforcement	Substituting unhealthy eating habits with healthy ones.
	Habit (automaticity)	Developing strategies that will help establish new habits.

COM-B, capability, opportunity and motivation model for behaviour change; MPF, minimally processed food; TDF, theoretical domains framework; UPF, ultraprocessed food.

Barriers and facilitators were linked to intervention functions. To form the foundational components of the programme, intervention functions were then linked to BCTs, which had been identified through further literature scoping as consistently being associated with successful dietary change. For example, a systematic review and meta-analysis of 48 studies indicated that goal setting and self-monitoring of behaviour, alongside a person-centred interaction, are particularly important for successfully promoting and maintaining healthy eating behaviour change in adults living with overweight/obesity.^43^ A systematic review of 25 studies found instructions on how to perform the behaviour, behaviour practice and self-monitoring to be the most promising BCTs to achieve weight loss in healthcare staff living with overweight and obesity.^44^ Goal setting, action planning, self-monitoring, restructuring the environment and problem solving emerged as key intervention requirements to encourage participants to reduce UPF consumption.^45^,^47^ We also used the theories and techniques tool to help guide the selection of BCTs.^48^

### UPDATE behavioural support programme

The resulting programme was multicomponent with online (Microsoft Teams; or in-person by participant choice) one-to-one behavioural support sessions, bespoke print resources, which were given to each participant, a website and regular support groups (approximately every 6 weeks), delivered over a 6-month period. The key content of the behavioural support programme mapped to the TDF is presented in table 3.

**Table 3:** Key components and BCTs mapped TDF domains covered in the UPDATE behavioural support programme

UPDATE intervention components*	Intervention component	BCTs and taxonomy grouping no.	TDF
Expertise and support	Introductions: introduce self as behavioural scientist working with the trial team at UCL	9.1. Credible source	Social influences
	Inform the participant we will send notes after the call because some people find it helpful to have records	6.2. Social comparison	Social influences
Introduction to booklet and website	Introduce the booklet/website	12.5. Adding object to the environment	Environmental context
Assess awareness of UPF prior to behavioural support/education	Ask if participant has heard of UPF; find out how confident they are at being able to recognise UPF in their food	2.3. Self-monitoring of behaviour	Knowledge
UPF scientific evidence	Introduce scientific evidence on UPF	5.1. Information about health consequences 9.1 Credible source	Knowledge beliefs about consequences
Education on Nova classification	Introducing Nova; endorsed by World Cancer Research Fund	9.1 Credible source	Social influences
	What UPFs are	5.1. Information about health consequences	Knowledge belief about consequences
	How to recognise UPF	4.1. Instruction on how to perform the behaviour	Skills
	Evidence on associations between UPF and health	5.1. Information about health consequences	Knowledge belief about consequences
Checking to see if understanding and confidence have grown after education components	Reassess how confident the participant is at recognising UPF now we have discussed them; discuss how they can increase their confidence; discuss what they have learnt so far about UPFs and their impact	15.1 Verbal persuasion about capability 4.4. Behavioural experiments 5.1. Information about health consequences	Beliefs about capabilities Knowledge beliefs about consequences
Feedback on diet prior to UPDATE participation	Inform the participant how much of their diet is made up of UPF; feedback on last 7 days food/beverage diary; ask participant where they think the UPF comes from in their current diet; identify their current dietary approach/pattern, how they shop, cook, etc.	2.2. Feedback on behaviour 1.6. Discrepancy between current behaviour and goal 5.1. Information about health consequences	Knowledge beliefs about consequences Skills and social influences
Notes and reflections	Discuss participant’s drivers/motivations for eating a diet high in UPF	2.3. Self-monitoring of behaviour 2.2. Feedback on behaviour	Behavioural regulation
Instruction on how to reduce UPF	Discuss benefits of reducing the amount of UPF in their diet and increasing MPF/unprocessed food; introduce ways to avoid UPF; discuss motivation to reduce UPF; discuss barriers to reducing UPF and brainstorm solutions	5.1. Information about health consequences 1.2. Problem solving 9.2. Pros and cons 1.2. Problem solving	Beliefs about consequences Beliefs about capabilities Intentions: goals Beliefs about capabilities Environmental context
Goal setting/action planning (behaviour)	Discuss goal(s) defined in terms of the behaviour to be achieved; promote goal-setting resource in booklet/website; discuss specific plans and introduce planning/rate confidence form to help secure goals are achieved	1.1. Goal setting (behaviour) 12.5. Adding objects to the environment 1.4. Action planning	Goals Environmental context and resources Behavioural regulation
Goal setting (outcome)	Discuss key goals in terms of a positive outcome of wanted behaviour	1.3. Goal setting (outcome)	Goals
Booklet/website resources: recipes, snack swaps, mapping of local area for food outlets	Go through resources, such as recommendations for recipes, cooking from scratch and local food environment availability.	1.2. Problem solving 4.1. Instructions on how to perform the behaviour	Environmental context and resources Knowledge
Habits	Describe habit formation through repetition of a behaviour, for example, looking regularly at goal/action planner	8.1. Behavioural practice/rehearsal 8.3. Habit formation	Skills
Self-monitoring	Explain purpose of self-monitoring and introduce tracker; ask them if they are self-monitoring/tracking activity in subsequent sessions	2.3. Self-monitoring of behaviour	Behavioural regulation
Support	Promote asking for support from friend/colleague/family member	3.3. Social support (emotional) 3.2. Social support (practical)	Social influences
Checking understanding and knowledge	Ask participant’s thoughts on the benefits of reducing UPF from their diet; check confidence at ability to recognise UPF	1.1. Problem solving 8.1. Behavioural practice/rehearsal	Beliefs about capabilities Skills
Goal map	Has participant designed a goal map?	1.7. Review outcome goal(s)	Goals
Assess success to date	Ask participant if they have reduced the amount of UPF in their diet	1.5. Review behaviour goal(s)	Goals
Goal setting/action planning	Remind participant of their goals and plans from last month; thoughts and feelings about goals set and action plan set last month; ask how they are getting on with their goals and plans	1.5. Review behaviour goal(s) 1.6. Discrepancy between current behaviour and goal	Goals
Habit	Ask participant if they are creating habits by looking regularly at goal/action plan and tracker	8.1. Behavioural practice/rehearsal 8.3. Habit formation	Skills
Provide feedback	Feedback on goal setting/action planning and activity so far; provide encouragement and enthusiasm for continued adherence to programme; monitor and provide informative or evaluative feedback on performance of the behaviour, for example, reduced number of UPF convenience foods, cooked more from scratch	2.2. Feedback on behaviour 2.7. Feedback on outcome of behaviour	Knowledge
Set new goal/action plan if relevant	Ask participant if they want to change their goals and plans for next month; analyse or prompt the participant to analyse factors influencing the behaviours and generate strategies that include overcoming the barriers and/or increasing facilitators	1.5. Review behaviour goal(s) 2.2. Problem solving	Goals Beliefs about capabilities

* In addition to UPF-related content, information and guidance around PA—including health benefits and instructions for increasing PA—were also provided as a part of the behavioural support.

BCT, behaviour change technique; PA, physical activity; TDF, theoretical domain framework; UCL, University College London; UPDATE, Ultra-Processed versus minimally processed Diets following UK dietAry guidance on healTh outcomEs; UPF, ultraprocessed food.

### Individual tailoring of the behavioural support programme

The two-stage design of UPDATE meant that some of the baseline data were collected 1–2 weeks before participants started the first diet in UPDATE Stage 1.^35^ During the baseline assessments, information was gathered to facilitate tailoring of the behavioural support programme. Sociodemographic data, including occupation and work pattern, as well as data on physical and mental health were collected as previously described.^35^

**Figure 1:**
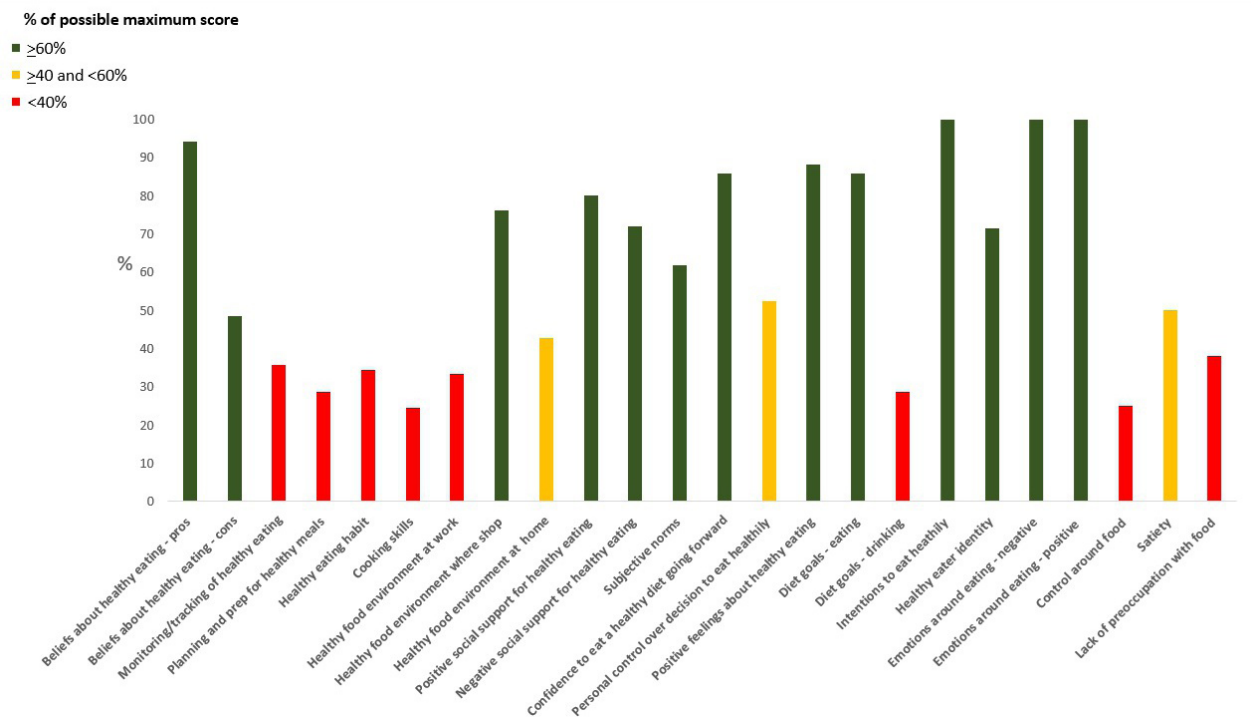
Exemplar graphical representation of a participant’s scores across various barriers and facilitators to reducing their UPF intake according to their responses to a capability, opportunity and motivation questionnaire. Responses are plotted against a possible maximum score for each domain. UPF, ultraprocessed food.

Participants completed a 257-item COM-B questionnaire on diet (n=120 items) and physical activity (n=137 items) from Willmott *et al*’s study,^49^ (with minor adaptation for UK setting) designed to capture rich quantitative data on the aspects of each participant’s attitudes, behaviours and beliefs as they relate to diet, appetite, healthy eating and physical activity. The full COM-B questionnaire is provided in online supplemental material 1. To provide an illustrative guide of barriers and facilitators for the behavioural scientist delivering the sessions, individual participants’ COM-B-TDF scores were summarised graphically, representing different barriers and facilitators, with participants’ responses plotted against a possible maximum score for each domain (see figure 1 for an exemplar). Assessing the COM-B questionnaire responses in the context of the participants’ broader sociodemographic, occupation and health circumstances was important for ensuring that the behavioural scientist (CB, EB or GNH) was able to deliver the intervention with suitable tailoring for each participant’s needs. For example, a participant who works night shifts as a nurse may be more likely to score lower on domains relating to opportunity than a participant who works from home in an administrative role, and therefore may need more support around goal setting and action planning.

Participants also completed the 15-item power of food scale (PFS)^50^ and the 21-item control of eating questionnaire (CoEQ).^51^ The PFS is used to assess the psychological impact of living in food-abundant environments and assesses appetite for palatable foods across three proximity domains, these being when such foods are (1) available, (2) present and (3) tasted. A five-point Likert scale from *do not agree at all* to *strongly agree* is used to measure responses to items across each domain, for example, for food present, “If I see or smell a food I like, I get a powerful urge to have some.” A score is generated for each domain, and an aggregate score of the mean of the three proximity domains is calculated. The CoEQ is used to measure the experience of food cravings across four subscales (craving control, craving for sweet, craving for savoury and positive mood), as well as measuring general appetite. Items are measured using a 100-mm visual analogue scale. Both the PFS and CoEQ have been validated and have Cronbach’s α of 0.81–0.91^50^ and 0.66–0.88,^51^ respectively. The behavioural scientists referred to each participant’s responses for these measures when considering factors that might influence their ability to adhere to dietary advice, which may influence the type and content of support delivered in the one-to-one behavioural support sessions.

Behavioural scientists also had access to participants’ baseline dietary recalls.^35^ Observing the participants’ habitual intake provided insight into their food preferences, eating patterns and typical food intake before they enrolled on the trial.

Overall, these data allowed the behavioural scientists to evaluate several aspects of the participants’ knowledge, beliefs and actions around healthy eating, diet planning, cooking skills, impact of work/home environment on choices, self-efficacy, healthy eating goals, affect/emotion around eating and motivations to eat. This facilitates identification of the combination of capability (participants’ knowledge and resources and skills in relation to healthy eating), opportunity (environmental factors that might impede healthy eating, eg, access to healthy food at work and social support) and motivation (emotional/cognitive issues impacting participants’ motivation to eat more healthily) factors, which should be targeted to elicit the required behaviour change.

### Individual behavioural support sessions

One-to-one behavioural support sessions were delivered for 6 months by a behavioural scientist (CB, EB and GNH). Each participant had all their behavioural support sessions delivered by the same behavioural scientist for the duration of the 6-month intervention. These sessions were delivered via video call or in person at University College London (UCL) to accommodate participant preference. All sessions were audio recorded using a Dictaphone for process evaluation. Initially, 1-hour sessions were delivered one time per month. However, early participant and behavioural scientist feedback indicated challenges with engagement with a 1-month gap between sessions, leading to changing the schedule to 30-min sessions every 2 weeks. The structure and content of these meetings are given in table 4.

**Table 4:** Structure and content of one-to-one behavioural support intervention sessions

Month	Content
Introduction Occurs shortly after participant finishes RCT diet 2 (Stage 1)	Introduction to the support programme Understanding motivations to take part in the trial Discuss experience of trial diets (Stage 1) Explore participant’s eating habits prior to the trial Brief discussion about UPF and MPF Goals and perceived barriers to achieving them Introduction to food/mood diary in the tracking booklet (online supplemental material 2)
Month 1 1 week after introduction meeting	Review food/mood diary Examine eating habits, emotions and behaviours associated with eating In-depth exploration of UPF and trial objectives using the behavioural support booklet (online supplemental material 3) Introduction to UPDATE intervention resources Quiz to test participant’s understanding of UPF Discussion of government dietary guidelines Ask participant to discuss their own UPF consumption habits Ask participant how they feel about reducing UPF Discuss barriers to and concerns about reducing UPF and increasing MPF Set goals and action plan for participant to achieve their goals Discuss self-monitoring in tracking booklet
Month 2	Catch up and review previous month’s goals Feedback on involvement in programme so far Feedback on goals and action plan Troubleshoot issues and barriers Encourage participant to do a goal outcome map If relevant, set new goals and relevant action plan Reiterate importance of self-monitoring; introduce idea of habit formation
Month 3	Catch up and review previous month’s goals Feedback on goals and action plan Troubleshoot issues and barriers If relevant, set new goals and relevant action plans Reiterate the importance of self-monitoring and habit formation In-depth exploration of PA Examine current PA levels, habits, barriers and concerns to doing more Encourage participants to set PA goals and action plans
Months 4–6	Review previous month How are they feeling now a few months have passed Compare dietary habits/PA behaviour now to pretrial Discuss progress to date Troubleshoot barriers/resistance/problems Make new goals and determine action plan for going forward Support programme feedback Plans going forward

MPF, minimally processed food; PA, physical activity; RCT, randomised controlled trial; UPDATE, Ultra-Processed versus minimally processed Diets following UK dietAry guidance on healTh outcomEs; UPF, ultraprocessed food.

For each participant, the discussion during the introductory meeting, as well as the quantitative data collected at baseline described above, informed the subsequent meetings and the approach used by the behavioural scientists to deliver the behavioural support. Subsequent meetings were used to discuss participants’ progress with reducing their UPF intake, troubleshoot barriers they may be encountering and maintain engagement and motivation. While this level of tailoring encouraged engagement and relevance for participants, the approach is clearly resource intensive. In future large-scale delivery, it may be possible to automate certain aspects, such as COM-B scoring. Furthermore, artificial inteligence-driven tools could be used to generate tailored feedback and suggest personalised strategies for participants.

### Print materials

In the period between completing the Stage 1 RCT and starting the Stage 2 behavioural intervention, participants received two booklets, which were developed to aid intervention delivery. The Stage 2 behavioural support booklet (online supplemental material 3) is an educational resource. It contains accessible information on what UPFs are, how to identify UPF, the evidence surrounding UPF and its associations with health, as well as general dietary advice, including the UK Government’s Eatwell Guide.^36^ The behavioural scientist guides participants through the booklet in the ‘Month 1’ individual support call, explaining the content at an appropriate level. During the third month of the Stage 2 behavioural support intervention, the behavioural scientist guides the participant through the booklet’s content on physical activity and exercise. The booklet also introduces the evidence around goal setting and action planning for achieving behaviour change with some examples.

The tracking booklet (online supplemental material 2) was designed to facilitate goal setting and habit tracking, which were identified as key BCTs for achieving weight loss in the literature scoping stage.^43 44^ The booklet prompts participants to set monthly goals, record their reasons for choosing them and outline when and how they plan to achieve them. There are pages for the participants to track their progress on achieving their goals each week, reflecting on barriers to achieving their goals and considering whether they are ready to add a new goal for the following month. Participants were encouraged to engage with the tracking booklet during their one-to-one behavioural support calls.

### Website

A mobile-optimised website was also created for participants to use (URL: findmempf.com; screenshots are provided in online supplemental material 4). The key features of the website include a recipe bank, information on UPF and how to identify it and the UK government Eatwell Guide. The website also features a food mapper, showing a range of food outlets (including chain eateries and supermarkets) within a 500-m radius of the main hospital site. These outlets were linked to a ‘Find Foods’ page with a directory of non-UPF products and menu items participants could refer to when considering replacements for UPF items in their diet. For example, a participant seeking a non-UPF bread could visit the Find Foods page, select ‘Bread’, and be taken to a landing page with hyperlinked lists of non-UPF breads available at major UK supermarkets. A participant could also select, for example, ‘Breakfast’, and be taken to a landing page with hyperlinked lists of non-UPF breakfast menu items at chain food outlets around the hospital site.

Product ingredient lists were assessed by GNH to ensure that they did not meet the Nova criteria for UPF and were reviewed regularly, including discussion with a specialist dietitian (ACB). The Find Foods page is also linked to a food map of the area around the central London hospital site, so the participants could identify local food outlets and the non-UPF items available there, including some chain restaurants and cafes.

In response to requests from participants, a 2-week low-UPF meal plan was developed and was uploaded to the website. Other resources featured on the website were a directory of podcast episodes, documentaries and videos on UPF, which had been deemed appropriate by the research team, the Portable Document Format (PDF) files of both booklets and information from the eating disorder charity, Beat. The website was continuously developed in response to participants’ feedback.

### Online group support sessions

Participants had the option to join an online peer discussion group hosted on a closed online meeting group moderated by a member of the behavioural support team. The purpose of this group was to enhance engagement over the 6-month intervention and to provide a forum for troubleshooting, ‘ask the expert’ sessions and peer and research team support.

The optional peer support group was introduced to participants during their first one-to-one support session. If participants expressed interest in joining, they were asked for verbal confirmation that they understood that if they joined the group, others on the trial (some of whom may be their colleagues) would know that they were taking part. For members of the peer support group, group sessions were held approximately every 6 weeks. All current and past participants were invited to attend. Group sessions consisted of a brief presentation by an expert in a relevant field, followed by a question-and-answer session and group discussion. The list of topics for these sessions is provided in table 5.

**Table 5:** Topics of group sessions

Title	Session content
Practical skills to deal with emotional eating	Meet the team and other participants Portion control Urge surfing Managing holidays, parties and Christmas Dealing with internal and external triggers Changing thoughts, feelings and behaviours Self-efficacy Stimulus control
Understanding emotional eating	Detailed information on emotional eating, including its origins, mechanisms and overall impact Insights into the consequences associated with emotional eating Practical advice on how to tackle emotional eating episodes
Understanding habits	What is a habit? Why do habits matter? How do we form habits? How habits can assist us in tackling emotional eating episodes Making and breaking habits—how to form good ones and how to disrupt bad ones Preparing your environment to form habits Planning and tracking
Emotional eating—in more depth	What is emotional eating? Why do people emotionally overeat? How might we change emotional eating? ACT and CBT for emotional eating What works? What can we improve?
UPF, calories and sustained weight loss	The science of calories and benefits of reducing UPF Achieving calorie deficit The difficulty in maintaining weight loss—physiological and emotional responses to weight reduction Behaviour associated with long-term success that is, self-monitoring, increased physical activity, reduced calorie intake and consistency Non-weight benefits of reducing UPF
UPF, MPF, processed food and food addiction	The science of UPF and why we care Food addiction—the evidence Weight gain and weight loss Policies and ‘big food’ What the future holds for food
ACT for managing unwanted eating behaviours	Understanding ACT: the science of acceptance and commitment therapy Emotional regulation: why it matters Mindfulness in eating: noticing urges without reacting Values-based eating: choosing food in line with long-term goals
Learnings from the trial; two trial participants discuss their experiences	Switching between UPF and MPF diets Cravings and withdrawal experiences Challenges of eating out and social situations Insights from the behavioural support programme Long-term changes and takeaways from the trial
Navigating UPF at Christmas	Strategies for navigating food-oriented celebrations Portion control Urge surfing Dealing with internal and external triggers ACT components; health values for Christmas Behaviour associated with long-term success, that is, self-monitoring, increased physical activity, reduced calorie intake and consistency
Revisiting the science of habits	What is a habit? Why do habits matter? How do we form habits? How habits can assist us in tackling emotional eating episodes Making and breaking habits—how to form good ones and how to disrupt bad ones Preparing your environment to form habits Planning and tracking

ACT, acceptance and commitment therapy; CBT, cognitive behavioural therapy; MPF, minimally processed food; UPF, ultraprocessed food.

### Moderated group chat

Participants also had the opportunity to join an optional closed discussion group hosted on a mobile messaging application (WhatsApp) moderated by a member of the behavioural support team using a designated UCL telephone number. The purpose of this group was to enhance engagement over the 6-month intervention and provide a forum for sharing hints, tips and encouragement, provide an opportunity for troubleshooting issues and a place for discussion (eg, about the science of UPF), as well as provide peer and research team support. As with the online meeting support group, this group was introduced to participants during their first one-to-one support session. If participants expressed interest in joining, they were asked for verbal confirmation that they understood that if they joined the group, others on the trial (some of whom may be their colleagues) would know they were taking part and that their mobile phone numbers would be visible.

### Exit interview

At the end of the behavioural support programme, whether they completed the programme or not, all participants were invited to take part in a qualitative exit interview.

## Exploratory evaluation: planned analyses

Stage 1 of UPDATE was a fully powered crossover trial, the full results of which have been published,^35^ whereas Stage 2—the behavioural support programme—was offered to all participants who took part in Stage 1 with the aim of gathering data on feasibility, uptake, retention and acceptability in order to further develop the intervention. Stage 2 was not designed to be statistically powered to detect change, and the nature of the two-stage design means that any comparisons between measures taken after Stage 2 to baseline must be considered as exploratory.

All participants embarked on Stage 2 after completing Stage 1, so it is feasible that experiences from the earlier phase might have influenced this later component. However, the potential carryover effect is not a concern for the planned exploratory evaluation of Stage 2, which will focus on feasibility and acceptability. Future pilot RCT testing will recruit participants who have not taken part in Stage 1 to provide a clearer assessment of intervention efficacy. Main analyses will be descriptive statistics on the proposed feasibility and acceptability outcomes described below.

Interest in the behavioural support programme will be assessed directly from a question in the exit interview: ‘were you interested in the behavioural support programme when you signed up or just the (Stage 1) diets?’ Uptake to Stage 2 will be assessed as the percentage of participants who agreed to start the behavioural support programme after completing Stage 1 of the trial. The feasibility of administering the intervention will be assessed as the percentage of participants who receive the key behavioural support content, which is delivered in the Month 1, one-to-one behavioural support sessions. Retention will be assessed as the percentage of participants who were still engaged with the behavioural support programme at 6 months.

The acceptability of the intervention will be assessed through several indicators. First, overall acceptability or usefulness of all aspects of the behavioural support programme, addressed during the exit interviews, will be assessed. Participants’ responses on the acceptability or usefulness of the individual aspects of the intervention (eg, booklets, one-to-one sessions and website) will also be assessed. Additionally, the percentage of one-to-one behavioural support sessions a participant was offered that they attended will be calculated. The percentage of participants who withdraw entirely from the behavioural support programme will also be reported.

Intervention delivery fidelity will be assessed via the application of BCT checklists to the transcribed intervention sessions. The scoring checklists for the one-to-one sessions are adapted from the BCTs outlined in table 3, and further detail on the scoring system, adapted from Cross *et al*’s study,^52^ is available in online supplemental material 5. Mean/median (SD/IQR) scores for the delivery of BCTs in each TDF domain will be presented.

As a part of the exploratory analyses, baseline and follow-up COM-B questionnaire scores in each component of the TDF will be compared using descriptives and appropriate simple comparison statistics (paired sample t-test or Wilcoxon test). Comparisons of clinical outcomes will also be conducted, including changes in bodyweight and body composition. Details on the collection of all clinical outcomes are available in the RCT protocol.^35^

## Ethics and dissemination

###  Ethical approval

The study is approved by The Yorkshire and The Humber—Sheffield Research Ethics Committee, who approved the trial on 22 December 2022 (22/YH/0281). The study was prospectively registered on ClinicalTrials.gov (NCT05627570). The behavioural support programme is ongoing and is being conducted in compliance with the principles of the Declaration of Helsinki 1996 and the principles of the International Council for Harmonisation Good Clinical Practice. Any amendments will be recorded in academic publications and will be submitted for approval to the Sponsor and Research Ethics Committee prior to implementation. Sponsor contact: (University College London Hospital/UCL) Joint Research Office (uclh.randd@nhs.net).

The following amendments have been made to the published study protocol.^35^

1. The initial protocol stated that the one-to-one behavioural support sessions would be delivered via telephone or video call^35^; however, on 27 July 2023, this was amended to allow sessions to be delivered via video call or in-person at UCL to accommodate participant preference.
2. The study protocol was amended on 27 July 2023 to add an online peer discussion group hosted on a closed online meeting group moderated by a member of the behavioural support team.
3. On 11 March 2024, an additional amendment was made to the published protocol to add an optional closed discussion group moderated by a member of the behavioural support team hosted on a mobile messaging application (WhatsApp).

### Dissemination

Results will be disseminated through peer-reviewed academic journals and will be presented at national and international conferences.

## Supplementary material

10.1136/bmjopen-2025-107435 online supplemental file 1

10.1136/bmjopen-2025-107435 online supplemental file 2

10.1136/bmjopen-2025-107435 online supplemental file 3

10.1136/bmjopen-2025-107435 online supplemental file 4

10.1136/bmjopen-2025-107435 online supplemental file 5
