# Supplementary material for: Development, content and planned evaluation of a behavioural support intervention to reduce ultraprocessed food intake and increase physical activity in UK healthcare workers: UPDATE trial stage 2 study protocol
Source: BMJ Open. 2025 Oct 29;15(10):e107435. doi: 10.1136/bmjopen-2025-107435 (PMC12574385; doi:10.1136/bmjopen-2025-107435)
Supplement: online supplemental file 1 [file bmjopen-15-10-s001.docx]

**DATE: ____________________ VISIT: _________________**

**Physical activity**

Please indicate whether the following statements are true or false. (tick one box in each row)

|  | True | False |
| --- | --- | --- |
| Adults aged 18 - 64 years should engage in at least 150 minutes of moderate intensity (i.e., exercises requiring some effort, but conversation is still possible) physical activity each week. |  |  |
| Adults aged 18-64 years should engage in at least 75 minutes of vigorous intensity (i.e., exercises which induce heaving breathing) physical activity each week. |  |  |
| An equivalent combination of both moderate and vigorous activities each week is sufficient (e.g., 1.5 hrs of moderate intensity + 1 hr of vigorous intensity exercise) for adults aged 18-64 years. |  |  |
| Adults aged 18-64 years should do muscle strengthening activities on at least 2 days each week. |  |  |
| 34% of men and 42% of women aged 18 years and over are either inactive or have low levels of physical activity. |  |  |
| Regular physical activity can reduce your risk of cardiovascular disease, type 2 diabetes, and some cancers, but only if it is performed at a moderate-vigorous intensity. |  |  |

Please read the following statements and indicate your level of agreement when it comes to making the decision of whether to exercise or not. (tick one box in each row)

|  | Strongly disagree | Disagree | Somewhat disagree | Neither disagree or agree | Somewhat agree | Agree | Strongly agree |
| --- | --- | --- | --- | --- | --- | --- | --- |
| I would have more energy for my family and friends if I exercised regularly. |  |  |  |  |  |  |  |
| Regular exercise would help me relieve tension. |  |  |  |  |  |  |  |
| I would feel more confident if I exercised regularly. |  |  |  |  |  |  |  |
| I would sleep more soundly if I exercised regularly. |  |  |  |  |  |  |  |
| I would feel good about myself if I kept my commitment to exercise regularly. |  |  |  |  |  |  |  |
| I would like my body better if I exercised regularly. |  |  |  |  |  |  |  |
| It would be easier for me to perform routine physical tasks if I exercised regularly. |  |  |  |  |  |  |  |
| I will feel less stressed if I exercise regularly. |  |  |  |  |  |  |  |
| I would be more comfortable with my body if I exercised regularly. |  |  |  |  |  |  |  |
| Regular exercise would help me have a more positive outlook on life. |  |  |  |  |  |  |  |
| I think I would be too tired to do my daily work after exercising. |  |  |  |  |  |  |  |
| I would find it difficult to find an exercise activity that I enjoy that is not affected by bad weather. |  |  |  |  |  |  |  |
| I feel uncomfortable when I exercise because I get out of breath and my heart beats very fast. |  |  |  |  |  |  |  |
| Regular exercise would take too much of my time. |  |  |  |  |  |  |  |
| I would have less time for my family and friends if I exercised regularly. |  |  |  |  |  |  |  |
| At the end of the day, I am too exhausted to exercise. |  |  |  |  |  |  |  |

Think about the last four weeks and indicate your level of agreement with the following statements. During the last four weeks I have... (tick one box in each row)

|  | Very untrue | Untrue | Somewhat untrue | Neutral | Somewhat true | True | Very true |
| --- | --- | --- | --- | --- | --- | --- | --- |
| Constantly monitored whether I exercise frequently enough. |  |  |  |  |  |  |  |
| Watched carefully that I exercise for at least 30 minutes at a moderate-intense level. |  |  |  |  |  |  |  |
| I have had my exercise intention often on my mind. |  |  |  |  |  |  |  |
| I have always been aware of my planned exercise regime. |  |  |  |  |  |  |  |
| I have really tried to exercise regularly. |  |  |  |  |  |  |  |
| Tried my best to act in accordance with my standards (for exercise). |  |  |  |  |  |  |  |

Think about the last four weeks and indicate your level of agreement with the following statements. During the last four weeks I have made a detail planned regarding... (tick one box in each row)

|  | Very untrue | Untrue | Somewhat untrue | Neutral | Somewhat true | True | Very true |
| --- | --- | --- | --- | --- | --- | --- | --- |
| When to do my physical exercise. |  |  |  |  |  |  |  |
| Where to exercise. |  |  |  |  |  |  |  |
| How to do my physical exercise. |  |  |  |  |  |  |  |
| How often to do my physical exercise. |  |  |  |  |  |  |  |

Please indicate your level of agreement with the following statements. Physical activity is something... (tick one box in each row)

|  | Strongly disagree | Disagree | Somewhat disagree | Neither disagree or agree | Somewhat agree | Agree | Strongly agree |
| --- | --- | --- | --- | --- | --- | --- | --- |
| I do frequently. |  |  |  |  |  |  |  |
| I do automatically. |  |  |  |  |  |  |  |
| I do without having to consciously remember. |  |  |  |  |  |  |  |
| That makes me feel weird if I do not do it. |  |  |  |  |  |  |  |
| I do without thinking. |  |  |  |  |  |  |  |
| That would require effort not to do it. |  |  |  |  |  |  |  |
| That belongs to my (daily, weekly, monthly) routine. |  |  |  |  |  |  |  |
| I start doing before I realise, I’m doing it. |  |  |  |  |  |  |  |
| I would find hard not to do. |  |  |  |  |  |  |  |
| I have no need to think about doing. |  |  |  |  |  |  |  |
| That’s typically “me.” |  |  |  |  |  |  |  |
| I have been doing for a long time. |  |  |  |  |  |  |  |

Please indicate which of the following statements most describes you. (tick one box in each row)

|  | Very untrue | Untrue | Somewhat untrue | Neutral | Somewhat true | True | Very true |
| --- | --- | --- | --- | --- | --- | --- | --- |
| I wish I could be a lot better at physical activities. |  |  |  |  |  |  |  |
| In physical activities I usually watch instead of participating. |  |  |  |  |  |  |  |
| I feel that others my age are generally better than me at physical activities. |  |  |  |  |  |  |  |
| I don’t feel I can do well at new physical activities. |  |  |  |  |  |  |  |

Please indicate which of the following statements most describes you. (tick one box in each row)

|  | Very untrue | Untrue | Somewhat untrue | Neutral | Somewhat true | True | Very true |
| --- | --- | --- | --- | --- | --- | --- | --- |
| I do very well at all kinds of physical activities. |  |  |  |  |  |  |  |
| I think I could do well at just about any new physical activity I haven’t tried before. |  |  |  |  |  |  |  |

Please read the following statements and indicate your level of agreement. (tick one box in each row)

|  | Strongly disagree | Somewhat disagree | Neither disagree or agree | Somewhat  agree | Strongly agree |
| --- | --- | --- | --- | --- | --- |
| My neighbourhood offers many opportunities to be physically active. |  |  |  |  |  |
| Local sports clubs and other similar providers in my neighbourhood offer many opportunities to get exercise. |  |  |  |  |  |
| It is pleasant to walk in my neighbourhood. |  |  |  |  |  |
| There are enough trees in my neighbourhood to provide shade. |  |  |  |  |  |
| In my neighbourhood it is easy to walk to places. |  |  |  |  |  |
| There are stores within walking distance of my home. |  |  |  |  |  |
| In my neighbourhood, the streets and sidewalk are in good condition. |  |  |  |  |  |
| I often see other people walking in my neighbourhood. |  |  |  |  |  |
| I often see other people exercising (e.g., jogging, cycling, playing sports) in my neighbourhood. |  |  |  |  |  |

Please read the following statements and indicate your level of agreement. (tick one box in each row)

|  | Strongly disagree | Somewhat disagree | Neither disagree or agree | Somewhat  agree | Strongly agree |
| --- | --- | --- | --- | --- | --- |
| My neighbourhood has heavy traffic. |  |  |  |  |  |
| There are busy roads to cross when out for walks in my neighbourhood. |  |  |  |  |  |

Please indicate if there are any of the following facilities within a 20-minute walk from your neighbourhood. (tick one box in each row)

|  | Yes | No |
| --- | --- | --- |
| Public Park. |  |  |
| Public (or private) sports field, basketball court, or tennis court. |  |  |
| Public (or private) park or pool. |  |  |
| Schools, colleges, or community centres with recreational facilities that are free and open to the public. |  |  |
| Gyms, health/fitness clubs, or pools that you must join and pay for. |  |  |
| YMCA or similar facilities. |  |  |
| Bicycle path in the street or park. |  |  |
| Pavements. |  |  |

Please read the following statements and indicate your level of agreement. (tick one box in each row)

|  | Strongly disagree | Disagree | Somewhat disagree | Neither disagree or agree | Somewhat  agree | Agree | Strongly agree |
| --- | --- | --- | --- | --- | --- | --- | --- |
| Most people in my social network want me to exercise regularly in the next 2 weeks. |  |  |  |  |  |  |  |
| Most people in my social network would approve if I exercised regularly in the next 2 weeks. |  |  |  |  |  |  |  |
| Most of my friends exercise regularly. |  |  |  |  |  |  |  |
| Most of my family members exercise regularly. |  |  |  |  |  |  |  |
| Most of my co-workers exercise regularly. |  |  |  |  |  |  |  |

During the past 12 months, how often have your family, friends, and experts... (tick one box in each row)

|  | Never | Almost never | Rarely | Sometimes | Usually | Almost always | Always |
| --- | --- | --- | --- | --- | --- | --- | --- |
| Exercised with me. |  |  |  |  |  |  |  |
| Offered to exercise with me. |  |  |  |  |  |  |  |
| Gave me helpful reminders to exercise (“Are you going to exercise tonight?”). |  |  |  |  |  |  |  |
| Gave me encouragement to stick with my exercise program. |  |  |  |  |  |  |  |
| Changed their schedule so we could exercise together. |  |  |  |  |  |  |  |
| Discussed exercise with me. |  |  |  |  |  |  |  |
| Gave me rewards for exercising (bought me something or gave me something I like). |  |  |  |  |  |  |  |
| Planned for exercise on recreational outings |  |  |  |  |  |  |  |
| Helped plan activities around my exercise. |  |  |  |  |  |  |  |
| Asked me for ideas on how they can get more exercise. |  |  |  |  |  |  |  |
| Talked about how much they like to exercise. |  |  |  |  |  |  |  |

During the past 12 months, how often have your family, friends, and experts... (tick one box in each row)

|  | Never | Almost never | Rarely | Sometimes | Usually | Almost always | Always |
| --- | --- | --- | --- | --- | --- | --- | --- |
| Complained about the time I spend exercising. |  |  |  |  |  |  |  |
| Criticised me or made fun of me for exercising. |  |  |  |  |  |  |  |

Please read the following statements and indicate your level of confidence or agreement. (tick one box in each row)

|  | Very unconfident | Unconfident | Somewhat unconfident | Neutral | Somewhat confident | Confident | Very confident |
| --- | --- | --- | --- | --- | --- | --- | --- |
| How confident are you that you will be able to exercise regularly in the next 2 weeks? |  |  |  |  |  |  |  |
| How confident are you over the next 2 weeks that you could overcome obstacles that prevent you from exercising regularly? |  |  |  |  |  |  |  |

|  | Very untrue | Untrue | Somewhat untrue | Neutral | Somewhat true | True | Very true |
| --- | --- | --- | --- | --- | --- | --- | --- |
| I believe I have the ability to regularly exercise in the next 2 weeks. |  |  |  |  |  |  |  |

Please read the following statements and indicate the level of agreement or control you feel. (tick one box in each row)

|  | Strongly disagree | Disagree | Somewhat disagree | Neither disagree or agree | Somewhat  agree | Agree | Strongly agree |
| --- | --- | --- | --- | --- | --- | --- | --- |
| Whether or not I exercise regularly in the next 2 weeks is entirely up to me. |  |  |  |  |  |  |  |

|  | No control | Almost no control | Little control | Some control | A lot of control | Almost complete control | Complete control |
| --- | --- | --- | --- | --- | --- | --- | --- |
| How much personal control do you feel you have over exercising regularly in the next 2 weeks? |  |  |  |  |  |  |  |

|  | Completely beyond my control | Almost completely beyond my control | A little within my control | Somewhat within my control | Quite within my control | Almost very much within my control | Very much within my control |
| --- | --- | --- | --- | --- | --- | --- | --- |
| How much do you feel that exercising regularly in the next 2 weeks is within your control? |  |  |  |  |  |  |  |

For me, exercising regularly over the next 2 weeks would be... (tick one box in each row)

|  | Harmful |  |  |  |  |  | Beneficial |
| --- | --- | --- | --- | --- | --- | --- | --- |
| For me, exercising regularly over the next 2 weeks would be... |  |  |  |  |  |  |  |
|  | Useless |  |  |  |  |  | Useful |
| For me, exercising regularly over the next 2 weeks would be... |  |  |  |  |  |  |  |
|  | Foolish |  |  |  |  |  | Wise |
| For me, exercising regularly over the next 2 weeks would be... |  |  |  |  |  |  |  |
|  | Boring |  |  |  |  |  | Interesting |
| For me, exercising regularly over the next 2 weeks would be... |  |  |  |  |  |  |  |
|  | Unenjoyable |  |  |  |  |  | Enjoyable |
| For me, exercising regularly over the next 2 weeks would be... |  |  |  |  |  |  |  |
|  | Stressful |  |  |  |  |  | Relaxing |
| For me, exercising regularly over the next 2 weeks would be... |  |  |  |  |  |  |  |

Personally, I exercise (or might exercise)... (tick one box in each row)

|  | Very untrue | Untrue | Somewhat untrue | Neutral | Somewhat true | True | Very true |
| --- | --- | --- | --- | --- | --- | --- | --- |
| To spend time with friends. |  |  |  |  |  |  |  |
| To enjoy the social aspects of exercising. |  |  |  |  |  |  |  |
| To have fun being active with other people. |  |  |  |  |  |  |  |
| To make new friends. |  |  |  |  |  |  |  |
| To help me look younger. |  |  |  |  |  |  |  |
| To have a good body. |  |  |  |  |  |  |  |
| To improve my appearance. |  |  |  |  |  |  |  |
| To look more attractive. |  |  |  |  |  |  |  |
| To give me goals to work towards. |  |  |  |  |  |  |  |
| To give me personal challenges to face. |  |  |  |  |  |  |  |
| To develop personal skills. |  |  |  |  |  |  |  |
| To measure myself against personal standards. |  |  |  |  |  |  |  |

Over the next three months I... (tick one box in each row)

|  | Strongly disagree | Disagree | Somewhat disagree | Neither disagree or agree | Somewhat agree | Agree | Strongly agree |
| --- | --- | --- | --- | --- | --- | --- | --- |
| Intend to engage in at least 30 minutes of moderate intensity physical activity, five times per week. |  |  |  |  |  |  |  |
| Determined to engage in at least 30 minutes of moderate intensity physical activity, five times per week. |  |  |  |  |  |  |  |
| Plan to engage in at least 30 minutes of moderate intensity physical activity, five times per week. |  |  |  |  |  |  |  |

The following questions concern your personal beliefs about exercise. Please indicate the degree to which you agree or disagree with each statement when thinking about your exercise participation. (tick one box in each row)

|  | Strongly disagree | Disagree | Somewhat disagree | Neither disagree or agree | Somewhat agree | Agree | Strongly agree |
| --- | --- | --- | --- | --- | --- | --- | --- |
| I consider myself an exerciser. |  |  |  |  |  |  |  |
| When I describe myself to others, I usually include my involvement in exercise. |  |  |  |  |  |  |  |
| I have numerous goals related to exercising. |  |  |  |  |  |  |  |
| Physical exercise is a central factor to my self-concept. |  |  |  |  |  |  |  |
| I need to exercise to feel good about myself. |  |  |  |  |  |  |  |
| Others see me as someone who exercises regularly. |  |  |  |  |  |  |  |
| For me, being an exerciser means more than just exercising. |  |  |  |  |  |  |  |
| I would feel a real loss if I were forced to give up exercising. |  |  |  |  |  |  |  |
| Exercising is something I think about often. |  |  |  |  |  |  |  |

Thinking about yourself and how you normally feel, to what extent do you generally feel the following emotions about physical activity. (tick one box in each row)

|  | Never | Rarely | Sometimes | Usually | Always |
| --- | --- | --- | --- | --- | --- |
| Determined. |  |  |  |  |  |
| Attentive. |  |  |  |  |  |
| Alert. |  |  |  |  |  |
| Inspired. |  |  |  |  |  |
| Active. |  |  |  |  |  |
| Afraid. |  |  |  |  |  |
| Nervous. |  |  |  |  |  |
| Upset. |  |  |  |  |  |
| Ashamed. |  |  |  |  |  |
| Hostile. |  |  |  |  |  |

Please indicate how often you do the following after exercising. (tick one box in each row)

|  | Never | Rarely | Sometimes | Usually | Always |
| --- | --- | --- | --- | --- | --- |
| I reward myself when I exercise. |  |  |  |  |  |
| I try to set realistic goals for myself rather than setting myself up for failure by expecting too much. |  |  |  |  |  |
| When I exercise, I tell myself that I am being good to myself by taking care of my body in this way. |  |  |  |  |  |
| I do something nice for myself for making efforts to exercise more. |  |  |  |  |  |

**Diet**

The first few items are about what advice you think experts are giving us.

Do health experts recommend that people should be eating more, the same amount, or less of the following foods? (tick one box per food)


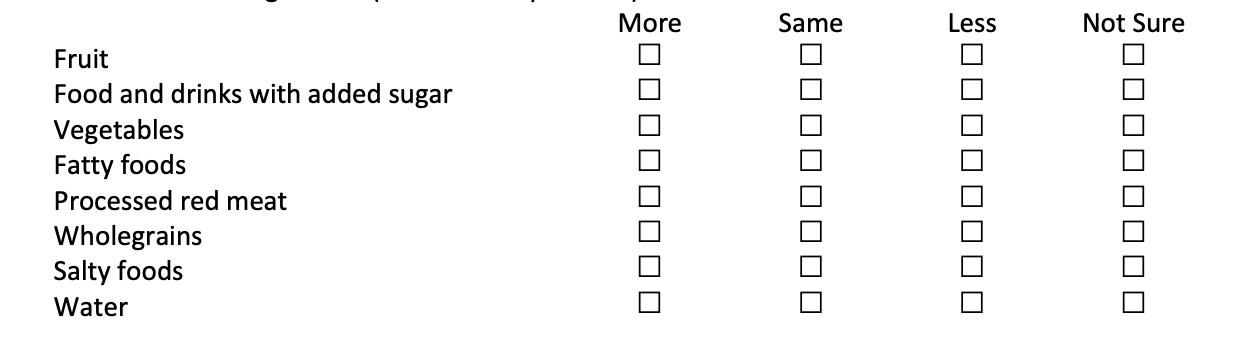


How many servings of fruit and vegetables per day do experts are advice people to eat as a Which of these types of fats do experts recommend that people should eat less of? (tick one box per food)


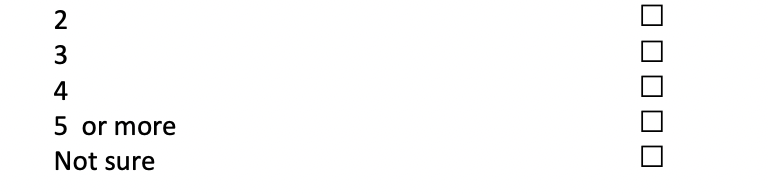


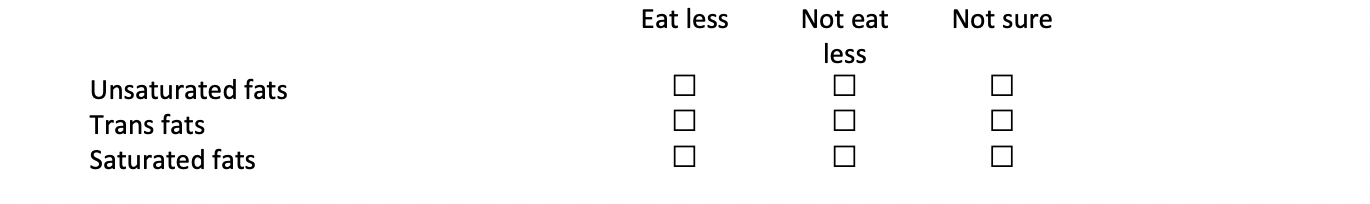
Which of these types of fats do experts recommend that people should eat less of? (tick one box per food)


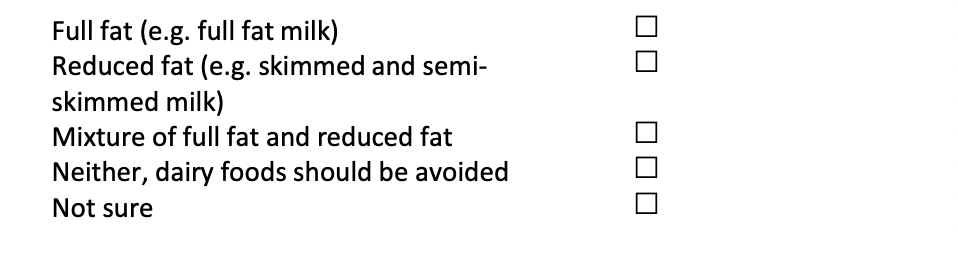
Which types of dairy foods do experts say people should drink? (tick one)

How many times per week do experts recommend that people eat oily fish (e.g. salmon and mackerel)? (tick one)


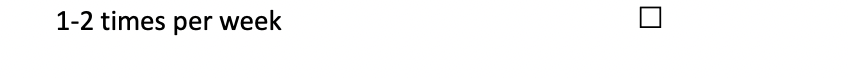

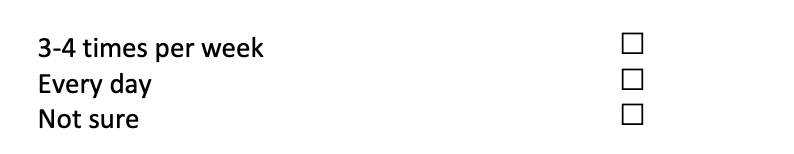


Approximately how many alcoholic drinks is the maximum recommended per day (The exact number depends on the size and strength of the drink)? (tick one)


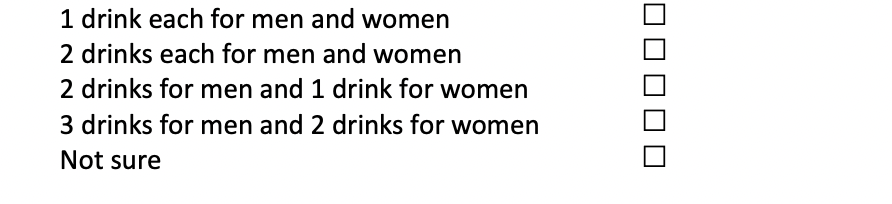


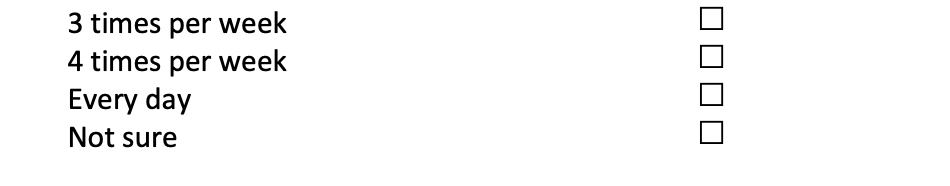
How many times per week do experts recommend that people eat breakfast? (tick one)

If a person has two glasses of fruit juice in a day, how many of their daily fruit and vegetable servings would this count as? (tick one)


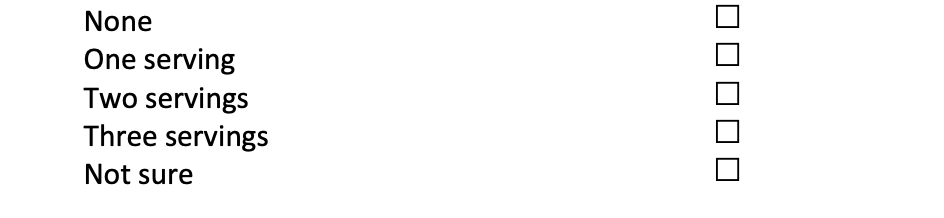


According to the ‘Eatwell guide’ (a guideline showing the proportions of food types people should eat to have a balanced and healthy diet), how much of people’s diet should be made up of starchy foods? (tick one)


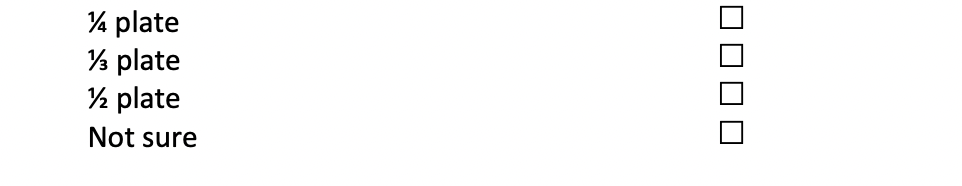


Experts classify foods into groups. We are interested to see whether people are aware of food groups and the nutrients they contain.

Do you think these foods and drinks are typically high or low in added sugar? (tick one box per food)


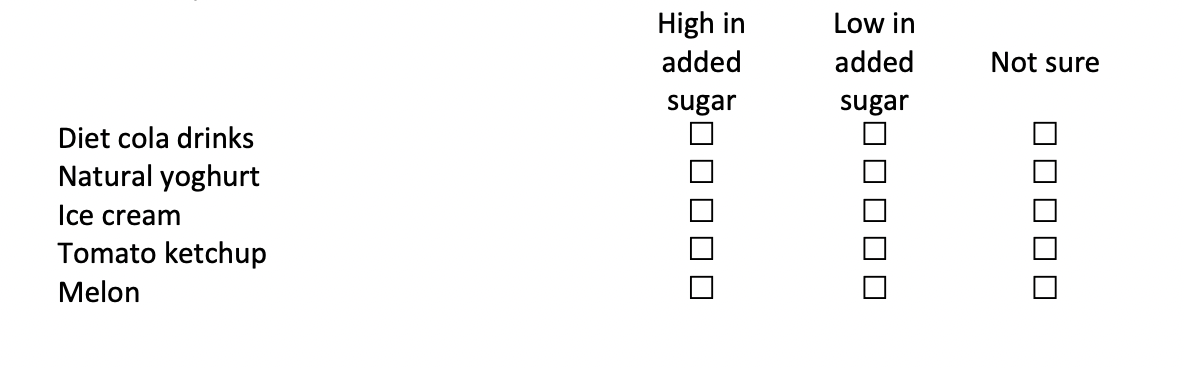


Do you think these foods are typically high or low in salt? (tick one box per food)
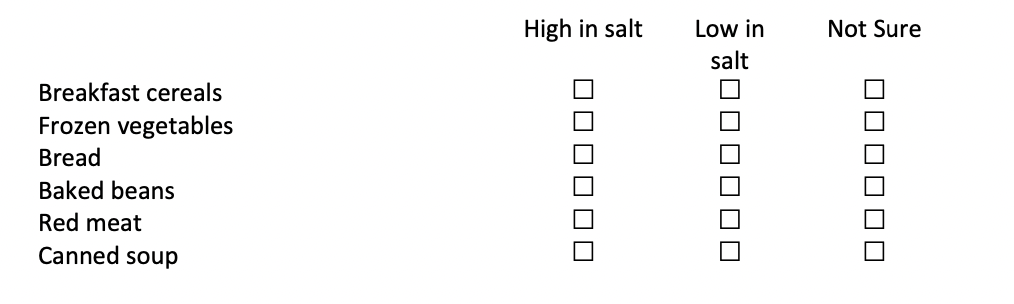


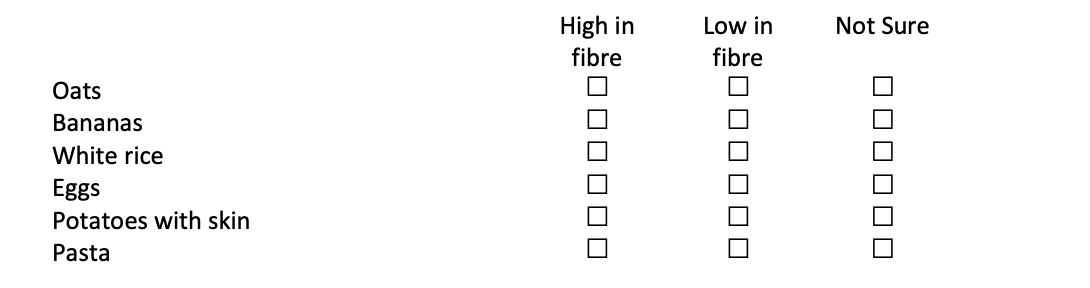
Do you think these foods are typically high or low in fibre? (tick one box per food)


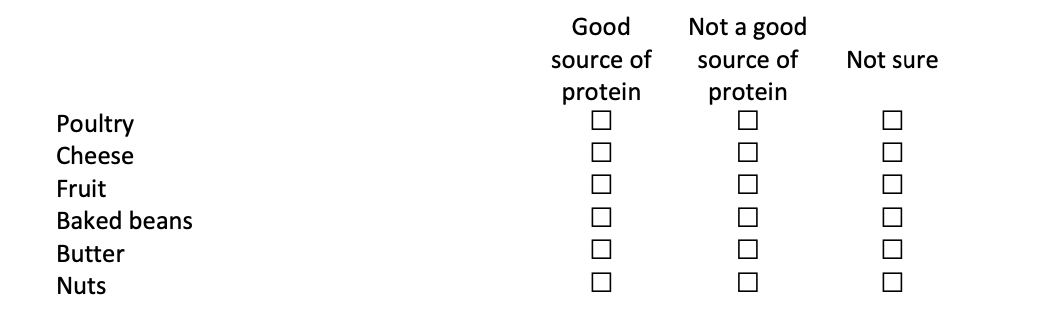
Do you think these foods are a good source of protein? (tick one box per food)


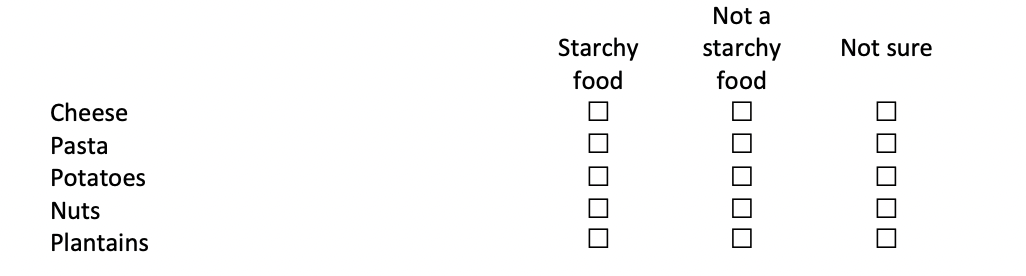
Which of the following foods do experts count as starchy foods? (tick one box per food)

Which is the main type of fat present in each of these foods? (tick one box per food)


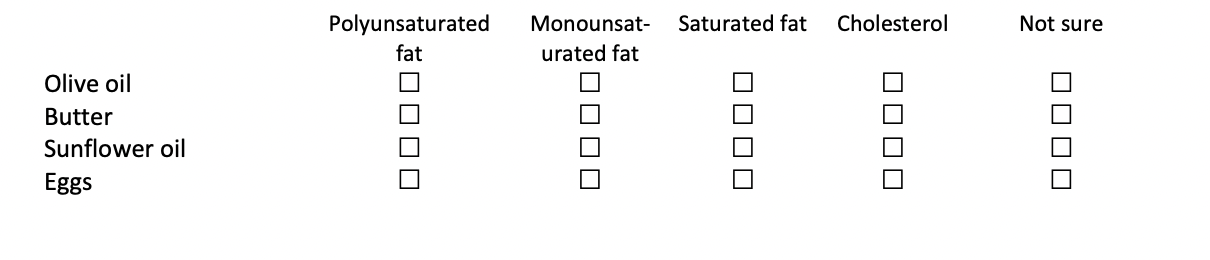


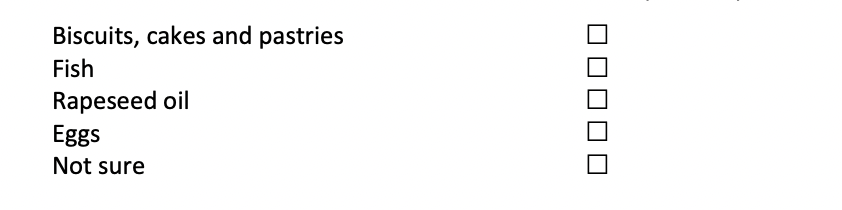
Which of these foods has the most trans-fat? (tick one)

The amount of calcium in a glass of whole milk compared to a glass of skimmed milk is: (tick one)


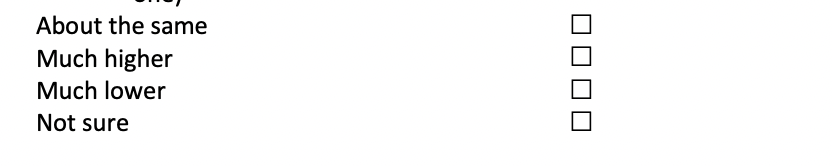


Which one of the following nutrients has the most calories for the same weight of food? (tick one)


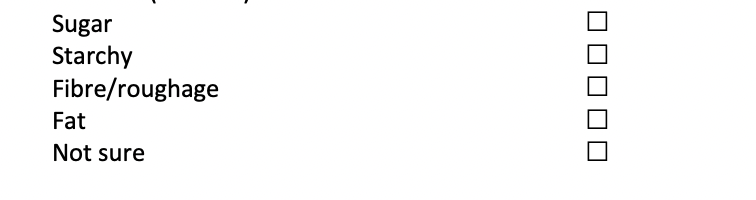


The next few items are about choosing foods.


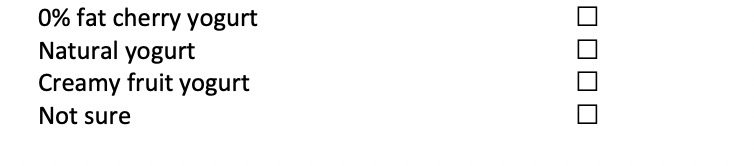
If a person wanted to buy a yogurt at the supermarket, which would have the least sugar/sweetener? (tick one)


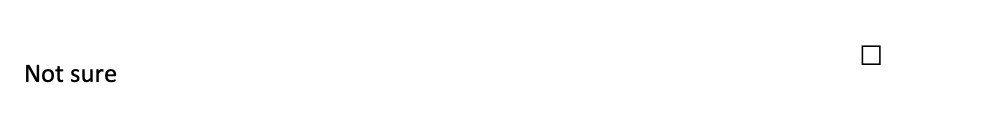
If a person wanted a soup in a restaurant or cafe, which one would be the lowest fat option? (tick one)


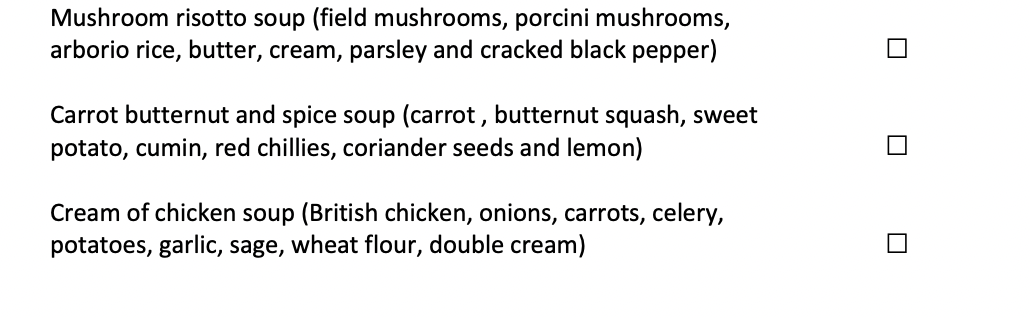


Which would be the healthiest and most balanced choice for a main meal in a restaurant? (tick one)


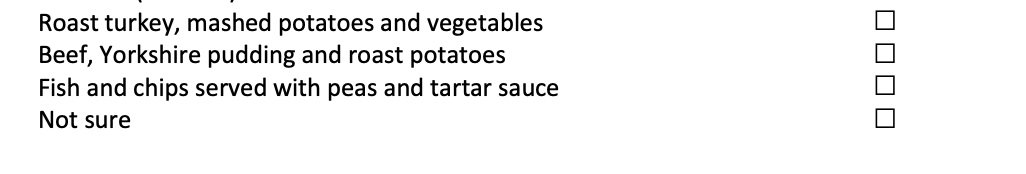


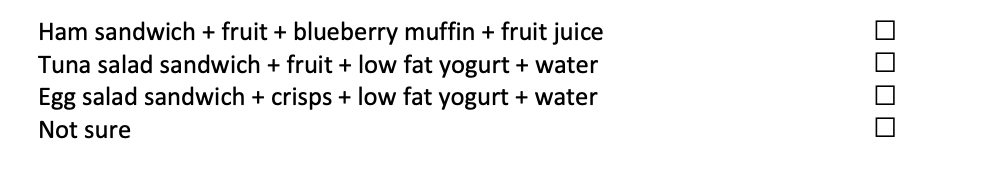
Which would be the healthiest and most balanced sandwich lunch? (tick one)

Which of these foods would be the healthiest choice for a pudding? (tick one)


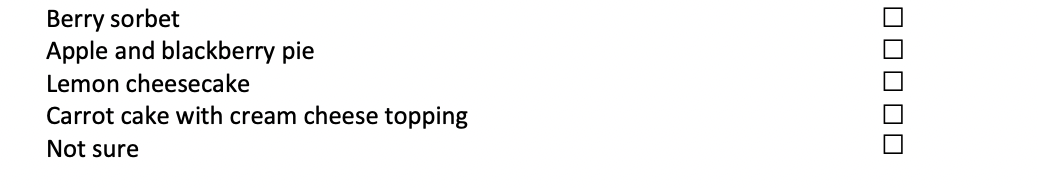


Which of these combinations of vegetables in a salad would give the greatest variety of vitamins and antioxidants? (tick one)


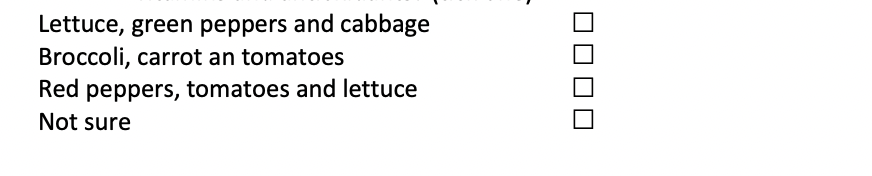


If a person wanted to reduce the amount of fat in their diet, but didn’t want to give up chips, which of the following foods would be the best choice? (tick one)


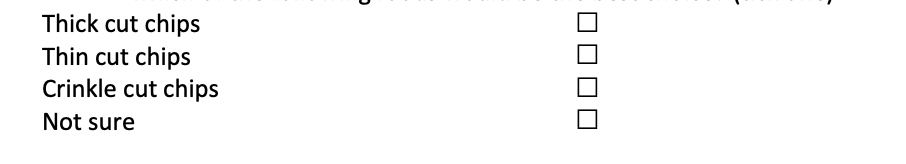


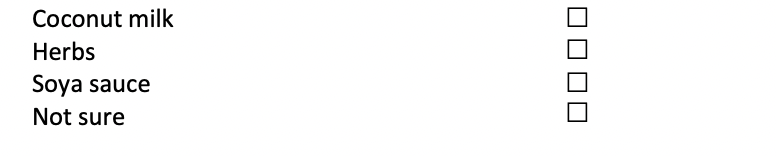
One healthy way to add flavour to food without adding extra fat or salt is to add: (tick one)


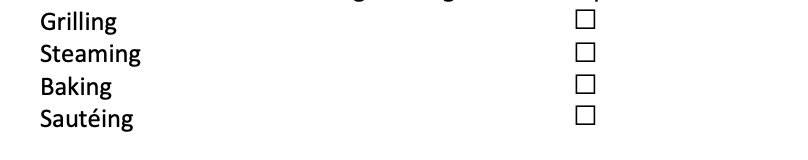
Which of the following cooking methods requires fat to be added? (tick one)

Traffic lights are often used on nutrition labelling, what would amber mean for the fat content of a food? (tick one)


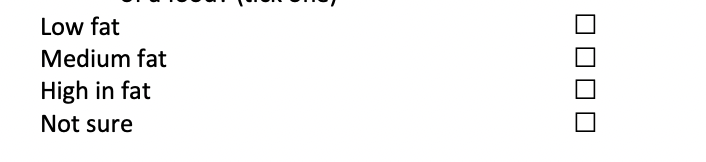


“Light” foods (or Diet foods) are always good options because they are low in calories. (tick one)


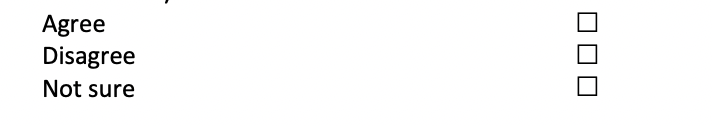


The following questions are related to food labels:


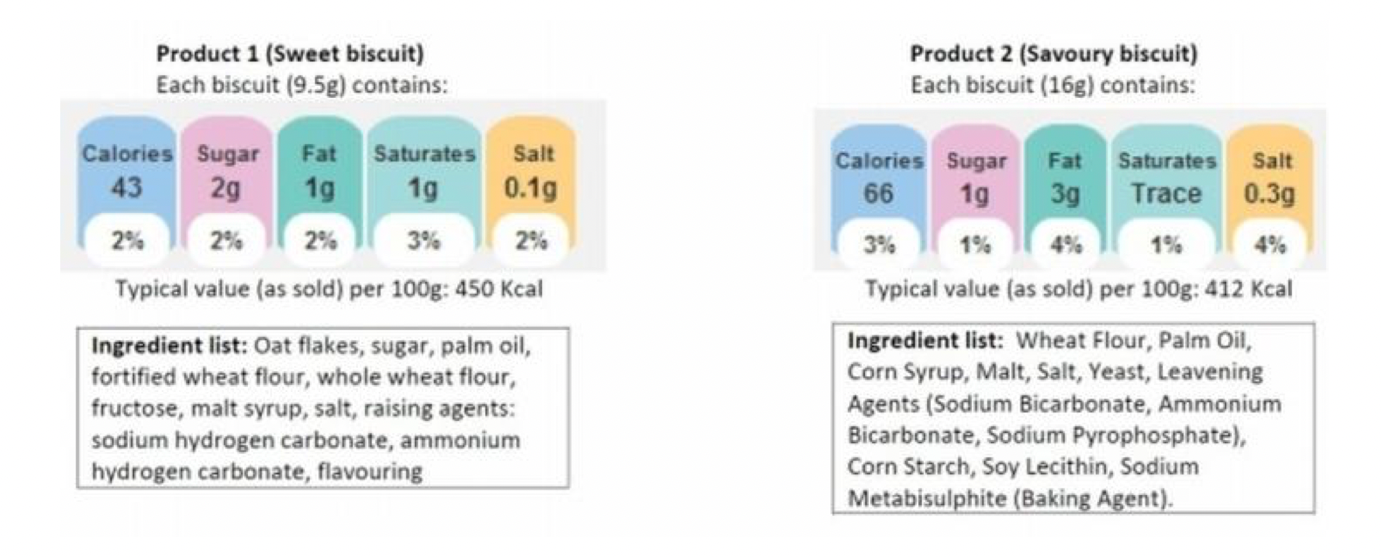


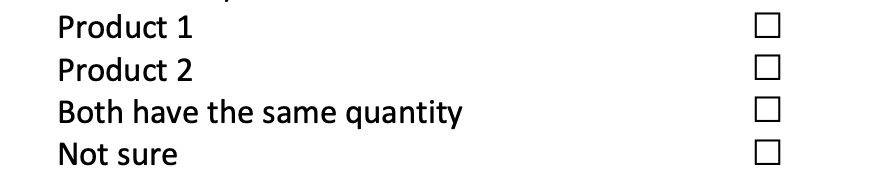
Looking at products 1 and 2, which one has the most calories (kcal) per 100 grams (tick one)


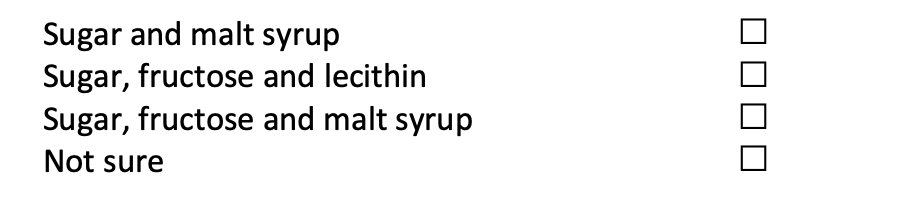
Looking at product 1, what are the sources of sugar in the ingredient list? (tick one)

Please read the following statements carefully and indicate your level of agreement with each. (tick one box in each row)

|  | Strongly disagree | Disagree | Somewhat disagree | Neither disagree or agree | Somewhat agree | Agree | Strongly agree |
| --- | --- | --- | --- | --- | --- | --- | --- |
| I would have more energy if I ate healthily. |  |  |  |  |  |  |  |
| I would be doing something good for my body if I ate healthily. |  |  |  |  |  |  |  |
| I would 'feel' good if I ate healthily. |  |  |  |  |  |  |  |
| Eating healthily would help me manage my weight. |  |  |  |  |  |  |  |
| I enjoy the taste of healthy foods. |  |  |  |  |  |  |  |
| It takes too much time to prepare healthy foods. |  |  |  |  |  |  |  |
| I would rather eat sweets or high fat snacks than healthy foods. |  |  |  |  |  |  |  |
| Healthy foods do not satisfy my hunger for very long. |  |  |  |  |  |  |  |
| Healthy foods are too expensive. |  |  |  |  |  |  |  |
| I don't like the taste of healthy foods. |  |  |  |  |  |  |  |

Think about the last four weeks and indicate your level of agreement with the following statements. During the last four weeks I have... (tick one box in each row)

|  | Strongly disagree | Disagree | Somewhat disagree | Neither disagree or agree | Somewhat agree | Agree | Strongly agree |
| --- | --- | --- | --- | --- | --- | --- | --- |
| Constantly monitored whether I am eating a healthy diet. |  |  |  |  |  |  |  |
| Watched carefully that I eat the recommended 5 servings of fruit and vegetables each day. |  |  |  |  |  |  |  |
| Had my healthy eating intention on my mind. |  |  |  |  |  |  |  |
| Always been aware of my plan to eat a healthy diet. |  |  |  |  |  |  |  |
| Really tried to eat healthily on most days of the week. |  |  |  |  |  |  |  |
| Tried my best to act in accordance with my standards (in relation to eating healthily). |  |  |  |  |  |  |  |

Think about the last four weeks and indicate the level to which the following statements are untrue/true for you. During the last four weeks I have made a detailed plan regarding... (tick one box in each row)

|  | Very untrue | Untrue | Somewhat untrue | Neutral | Somewhat true | True | Very true |
| --- | --- | --- | --- | --- | --- | --- | --- |
| When to do my healthy meal preparations for the week. |  |  |  |  |  |  |  |
| Where to shop for my healthy meal preparations. |  |  |  |  |  |  |  |
| How I will approach my meal preparation for the week (e.g., cookbook, download recipes from online website, download an app etc.). |  |  |  |  |  |  |  |
| How often to do my meal preparations (e.g., cook meals for next 2-3 days only OR cook and freeze pre-made meals for the entire week). |  |  |  |  |  |  |  |

Please indicate your level of agreement with the following statements. Healthy eating is something... (tick one box in each row)

|  | Strongly disagree | Disagree | Somewhat disagree | Neither disagree or agree | Somewhat agree | Agree | Strongly agree |
| --- | --- | --- | --- | --- | --- | --- | --- |
| I do frequently. |  |  |  |  |  |  |  |
| I do automatically. |  |  |  |  |  |  |  |
| I do without having to consciously remember. |  |  |  |  |  |  |  |
| that makes me feel guilty if I do not do it. |  |  |  |  |  |  |  |
| I do without thinking. |  |  |  |  |  |  |  |
| I find effortless to do (e.g., not eating healthy would require more effort than eating healthy for you). |  |  |  |  |  |  |  |
| that belongs to my (daily, weekly, monthly) routine (e.g., you eat a healthy diet every day). |  |  |  |  |  |  |  |
| that requires little thought. |  |  |  |  |  |  |  |
| I would find hard not to do. |  |  |  |  |  |  |  |
| I have no need to think about doing. |  |  |  |  |  |  |  |
| That’s typically “me.” |  |  |  |  |  |  |  |
| I have been doing for a long time. |  |  |  |  |  |  |  |

Please indicate which of the following statements most describes you. (tick one box in each row)

|  | Strongly disagree | Disagree | Somewhat disagree | Neither disagree or agree | Somewhat agree | Agree | Strongly agree |
| --- | --- | --- | --- | --- | --- | --- | --- |
| I can cook complicated multi-course meals. |  |  |  |  |  |  |  |
| I can prepare a lot of meals even without a recipe. |  |  |  |  |  |  |  |
| I can prepare gratin (basic culinary technique - potato topped with a browned crust). |  |  |  |  |  |  |  |
| I can prepare a soup. |  |  |  |  |  |  |  |
| I can prepare a sauce. |  |  |  |  |  |  |  |
| I can bake a cake. |  |  |  |  |  |  |  |
| I can bake bread. |  |  |  |  |  |  |  |

Please indicate your level of agreement with the following statements. (tick one box in each row)

|  | Strongly disagree | Disagree | Somewhat disagree | Neither disagree or agree | Somewhat agree | Agree | Strongly agree |
| --- | --- | --- | --- | --- | --- | --- | --- |
| At work there is a wide variety of healthy food options to choose from. |  |  |  |  |  |  |  |
| At work there is a wide variety of healthy drink options to choose from. |  |  |  |  |  |  |  |
| At work there is at least one option where I have healthy selections to choose from. |  |  |  |  |  |  |  |
| There is a wide variety of fresh fruits and vegetables where I shop. |  |  |  |  |  |  |  |
| The fruits and vegetables where I shop are at good prices. |  |  |  |  |  |  |  |
| The fruits and vegetables where I shop are of good quality. |  |  |  |  |  |  |  |
| At home there are healthy snacks available to eat. |  |  |  |  |  |  |  |
| At home there are healthy drinks available. |  |  |  |  |  |  |  |
| At home fruit is always available to eat. |  |  |  |  |  |  |  |
| At home vegetables are always available to eat. |  |  |  |  |  |  |  |

Please read the following statements carefully and indicate your level of agreement. (tick one box in each row)

|  | Strongly disagree | Disagree | Somewhat disagree | Neither disagree or agree | Somewhat agree | Agree | Strongly agree |
| --- | --- | --- | --- | --- | --- | --- | --- |
| Most people in my social network think I should eat a healthy diet in the next 2 weeks. |  |  |  |  |  |  |  |
| Most people in my social network want me to eat a healthy diet in the next 2 weeks. |  |  |  |  |  |  |  |
| Most people in my social network would approve if I ate a healthy diet in the next 2 weeks. |  |  |  |  |  |  |  |
| Most of my friends eat a healthy diet. |  |  |  |  |  |  |  |
| Most of my family eat a healthy diet. |  |  |  |  |  |  |  |
| Most of my co-workers eat a healthy diet. |  |  |  |  |  |  |  |

How often in the past month (30 days) has your family or friends done the following? (tick one box in each row)

|  | Almost never | Rarely | Sometimes | Usually | Almost always |
| --- | --- | --- | --- | --- | --- |
| Encouraged you to eat healthy foods. |  |  |  |  |  |
| Discussed the benefits of eating healthy foods with you. |  |  |  |  |  |
| Reminded you to eat healthy foods. |  |  |  |  |  |
| Complimented you on your healthy eating habits. |  |  |  |  |  |
| Commented if you went back to old “unhealthy” eating habits. |  |  |  |  |  |
| Ate unhealthy foods in front of you. |  |  |  |  |  |
| Refused to eat healthy foods with you. |  |  |  |  |  |
| Brought home foods you were trying not to eat. |  |  |  |  |  |
| Complained about eating healthy foods. |  |  |  |  |  |
| Offered me food I􏰀m trying not to eat. |  |  |  |  |  |

Please read the following statements and indicate your level of confidence, difficulty, or perceived ability. (tick one box in each row)

|  | Very unconfident | Unconfident | Somewhat unconfident | Neutral | Somewhat confident | Confident | Very confident |
| --- | --- | --- | --- | --- | --- | --- | --- |
| How confident are you that you will be able to eat healthily over the next 2 weeks? |  |  |  |  |  |  |  |

|  | Very difficult | Difficult | Somewhat difficult | Neutral | Somewhat easy | Easy | Very easy |
| --- | --- | --- | --- | --- | --- | --- | --- |
| For me to eat healthily over the next 2 weeks would be: |  |  |  |  |  |  |  |

|  | Very untrue | Untrue | Somewhat untrue | Neutral | Somewhat true | True | Very true |
| --- | --- | --- | --- | --- | --- | --- | --- |
| I believe I have the ability to eat healthily in the next 2 weeks. |  |  |  |  |  |  |  |

Please read the following statement and indicate your level of agreement. (tick one box in each row)

|  | Strongly disagree | Disagree | Somewhat disagree | Neither disagree or agree | Somewhat agree | Agree | Strongly agree |
| --- | --- | --- | --- | --- | --- | --- | --- |
| Whether or not I eat healthily in the next 2 weeks is entirely up to me. |  |  |  |  |  |  |  |

|  | No control | Almost no control | Little control | Some control | A lot of control | Almost complete control | Complete control |
| --- | --- | --- | --- | --- | --- | --- | --- |
| How much control do you feel you have overeating healthily in the next 2 weeks? |  |  |  |  |  |  |  |

|  | Completely beyond my control | Almost completely beyond my control | A little within my control | Somewhat within my control | Quite within my control | Almost very much within my control | Very much within my control |
| --- | --- | --- | --- | --- | --- | --- | --- |
| How much do you feel that eating healthily in the next 2 weeks is within your control? |  |  |  |  |  |  |  |

For me, eating a healthy diet over the next 2 weeks would be... (tick one box in each row)

|  | Good |  |  |  |  |  | Bad |
| --- | --- | --- | --- | --- | --- | --- | --- |
| For me, eating a healthy diet over the next 2 weeks would be... |  |  |  |  |  |  |  |
|  | Harmful |  |  |  |  |  | Beneficial |
| For me, eating a healthy diet over the next 2 weeks would be... |  |  |  |  |  |  |  |
|  | Foolish |  |  |  |  |  | Wise |
| For me, eating a healthy diet over the next 2 weeks would be... |  |  |  |  |  |  |  |
|  | Unenjoyable |  |  |  |  |  | Enjoyable |
| For me, eating a healthy diet over the next 2 weeks would be... |  |  |  |  |  |  |  |
|  | Unpleasant |  |  |  |  |  | Pleasant |
| For me, eating a healthy diet over the next 2 weeks would be... |  |  |  |  |  |  |  |
|  | Stressful |  |  |  |  |  | Relaxing |
| For me, eating a healthy diet over the next 2 weeks would be... |  |  |  |  |  |  |  |

In the past month, how often have you set a goal to... (tick one box in each row)

|  | Very untrue | Untrue | Somewhat untrue | Neutral | Somewhat true | True | Very true |
| --- | --- | --- | --- | --- | --- | --- | --- |
| Eat more fruits. |  |  |  |  |  |  |  |
| Eat more vegetables. |  |  |  |  |  |  |  |
| Eat less sweets. |  |  |  |  |  |  |  |
| Eat fewer fast food/take-out. |  |  |  |  |  |  |  |
| Drink more water. |  |  |  |  |  |  |  |
| Drink less sugar-sweetened drinks. |  |  |  |  |  |  |  |
| Drink less caffeine. |  |  |  |  |  |  |  |
| Drink less alcohol (e.g., beer, wine, hard liquor). |  |  |  |  |  |  |  |
| Eat a balanced diet. |  |  |  |  |  |  |  |
| Maintain healthy changes that you have already made to your diet. |  |  |  |  |  |  |  |

Over the next three months I... (tick one box in each row)

|  | Strongly disagree | Disagree | Somewhat disagree | Neither disagree or agree | Somewhat agree | Agree | Strongly agree |
| --- | --- | --- | --- | --- | --- | --- | --- |
| Intend to eat a healthy diet. |  |  |  |  |  |  |  |
| Will try to eat a healthy diet. |  |  |  |  |  |  |  |
| Want to eat a healthy diet. |  |  |  |  |  |  |  |
| Expect to eat a healthy diet. |  |  |  |  |  |  |  |
| Am likely to eat a healthy diet. |  |  |  |  |  |  |  |

Please indicate your level of agreement with the following statements. (tick one box in each row)

|  | Strongly disagree | Disagree | Somewhat disagree | Neither disagree or agree | Somewhat agree | Agree | Strongly agree |
| --- | --- | --- | --- | --- | --- | --- | --- |
| I think of myself as a healthy eater. |  |  |  |  |  |  |  |
| I think of myself as a person who is interested in eating healthy. |  |  |  |  |  |  |  |
| I think of myself as someone who is concerned about the health consequences of what I eat. |  |  |  |  |  |  |  |

Please indicate the extent to which you associate the following emotions with eating. (tick one box in each row)

|  | Not at all associated | A little associated | Somewhat associated | Associated | Very strongly associated |
| --- | --- | --- | --- | --- | --- |
| Feeling of anger (e.g., angry/annoyed). |  |  |  |  |  |
| Feeling of fear (e.g., anxious/fearful). |  |  |  |  |  |
| Feeling of sadness (e.g., sad/depressed). |  |  |  |  |  |
| Feeling of joy (e.g., joyful/happy). |  |  |  |  |  |
| Feeling of tension (e.g., tense/excited). |  |  |  |  |  |
| Feeling of relaxation (e.g., at ease/relaxed). |  |  |  |  |  |

Please indicate the extent to which the following statements are true for you. (tick one box in each row)

|  | Very untrue | Untrue | Somewhat untrue | Neutral | Somewhat true | True | Very true |
| --- | --- | --- | --- | --- | --- | --- | --- |
| I eat when I have feelings of hunger. |  |  |  |  |  |  |  |
| I eat when I have a desire to eat. |  |  |  |  |  |  |  |
| I eat when I have bodily symptoms of hunger (e.g., empty feeling in stomach, stomach rumbling). |  |  |  |  |  |  |  |
| I have a tendency to eat irregularly. |  |  |  |  |  |  |  |
| I have a tendency to eat sweet food (e.g., chocolate). |  |  |  |  |  |  |  |
| I have a tendency to eat good tasting food. |  |  |  |  |  |  |  |
| I have a tendency to enjoy food. |  |  |  |  |  |  |  |
| I have a tendency to eat to provide distraction. |  |  |  |  |  |  |  |
| I have a tendency to eat something to relax. |  |  |  |  |  |  |  |
| I have a tendency to eat to feel better. |  |  |  |  |  |  |  |

Please indicate your level of agreement with the following statements. (tick one box in each row)

|  | Strongly disagree | Disagree | Somewhat disagree | Neither disagree or agree | Somewhat agree | Agree | Strongly agree |
| --- | --- | --- | --- | --- | --- | --- | --- |
| I feel out of control in the presence of delicious food. |  |  |  |  |  |  |  |
| When I start eating, I just can’t seem to stop. |  |  |  |  |  |  |  |
| It is difficult for me to leave food on my plate. |  |  |  |  |  |  |  |
| When it comes to foods I love, I have no willpower. |  |  |  |  |  |  |  |
| I get so hungry that my stomach often seems like a bottomless pit. |  |  |  |  |  |  |  |
| I don’t get full easily. |  |  |  |  |  |  |  |
| It seems like most of my waking hours are preoccupied by thoughts about eating or not eating. |  |  |  |  |  |  |  |
| I have days when I can􏰀t seem to think about anything else but food. |  |  |  |  |  |  |  |
| Food is always on my mind. |  |  |  |  |  |  |  |
