# Supplementary material for: Development, content and planned evaluation of a behavioural support intervention to reduce ultraprocessed food intake and increase physical activity in UK healthcare workers: UPDATE trial stage 2 study protocol
Source: BMJ Open. 2025 Oct 29;15(10):e107435. doi: 10.1136/bmjopen-2025-107435 (PMC12574385; doi:10.1136/bmjopen-2025-107435)
Supplement: online supplemental file 2 [file bmjopen-15-10-s002.pdf]

UPDATE

UPDATE TRACKING JOURNAL

This journal is designed to be used along with the **UPDATE Behavioural calls.** You do not have to open this until you have your introductory call, when the behavioural scientist will explain to you what this is for and how to use each of the sections.

UCL

Welcome

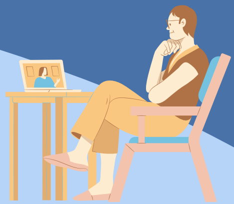

You are now coming to the end of the first part of the UPDATE trial and are about to move on to the Behavioural Support programme.

The overall study is led by [redacted]

This tracker is designed to be used with your UPDATE behavioural support calls. If you haven't booked your first call, please email [redacted] to arrange this

The **Behavioural Science** team are [redacted]

The Behavioural Support Programme is a combination of behavioural support calls, print materials, a website and optional group based sessions. You will get access to all of these in the coming months. However, right now we don't need to you do anything at all. Just hold on to this tracker and ideally have it with you for your **Introductory call.** The behavioural scientist will talk you through the next steps during the call.

SECTION 1: FOOD & MOOD DIARY

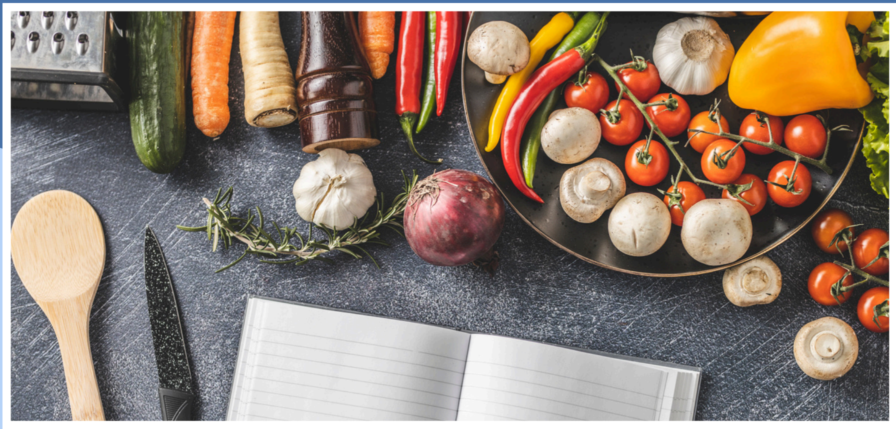

This section is designed to be completed **after your Introductory Call**. You do not need to do anything with it until then.

UPDATE

Please fill out the diary as accurately as possible. Use the example diary on the next page as a guide.

- Write down all the food and drinks you consume and any activity you do for at least 3 days (including a weekend)
- Include all snacks (and supplements if you take any)
- Include all alcoholic and all non alcoholic drinks
- Record the time that you consume the meal, snack, drink or do the activity
- Describe food and drink in as much detail as possible
- Rate your hunger before you eat using the hunger scale below
- Write down where you are and who you are with when eating and drinking
- Record your mood/feelings and/or any symptoms and the time they occur

Hunger scale

1	2	3	4	5	6	7	8	9	10
Physically faint	Ravenous	Fairly hungry	Slightly Hungry	Neutral	Pleasantly satisfied	Full	Stuffed	Bloated	Nauseous

UCL

An example of how to fill in your diary

[This is not a suggestion of what to eat]

Food/Drink/Activity	Quantity	Where/Who with	Feelings/Symptoms
Breakfast<br>***8.00am***<br>*Rice Krispies with semi-skimmed milk*	*Medium sized bowl, not much milk because didn't have much left!*	*At home, sitting at the table*<br>*Alone*	Hunger=3
Mid morning<br>***11.00am***<br>*Walkers salt and vinegar crisps*<br>*Ribena*	*1 packet*<br>*1 juice box*	*At work*<br>*With 1 colleague- Sue*	Hunger=5<br>Felt tired
Lunch<br>***12.30pm***<br>*Chicken wrap from Tesco*<br>*Apple*<br>*Kitkat*<br>*Diet Coke*<br><br>*Walked to a different building quite briskly*	*All of it (2 halves)*<br>*Whole thing*<br>*Two sticks*<br>*Most of the can*<br><br>*10 mins there and ten mins back*	*At work*<br>*With colleagues- Martin, Edward and Tina*	Hunger=9<br><br>Feeling bloated
Mid afternoon<br>***3.30pm***<br>*McVitie's milk chocolate digestive biscuits*<br>*Cappucino (machine in hospital) with sugar*	*3*<br>*I cup and I teaspoon sugar*	*At work*<br>*On my own*	Hunger=5
Evening meal<br>***7.30pm***<br>*Vegetarian mince meat burgers*<br>*Baked beans*<br>*Mashed potatoes with added butter and milk*<br>*Walls vanilla ice cream*	*2*<br>*2 tbsps*<br>*Big scoop*<br>*3 scoops*	At home<br>*With partner and kids*	Hunger=3<br>Felt full after I had eaten and sleepy
Bedtime<br>***9.00pm***<br><br>*Hot chocolate (Packet)*<br>*Rich tea biscuits*	*1 cup of milk*<br>*3*	*At home watching television*<br>*Alone*	Hunger=5<br><br>Habit, routine

Day 1

Food/drink/activity	Quantity	Where/who with	Feelings/symptoms
Breakfast __:__			
Mid-morning __:__			
Lunch __:__			
Mid-afternoon __:__			
Evening meal __:__			
Bedtime __:__			

Day 2

Food/drink/activity	Quantity	Where/who with	Feelings/symptoms
Breakfast<br>__:__			
Mid-morning<br>__:__			
Lunch<br>__:__			
Mid-afternoon<br>__:__			
Evening meal<br>__:__			
Bedtime<br>__:__			

Day 3

Food/drink/activity	Quantity	Where/who with	Feelings/symptoms
Breakfast<br>__:__			
Mid-morning<br>__:__			
Lunch<br>__:__			
Mid-afternoon<br>__:__			
Evening meal<br>__:__			
Bedtime<br>__:__			

UPDATE

SECTION 2: DIET TRACKING

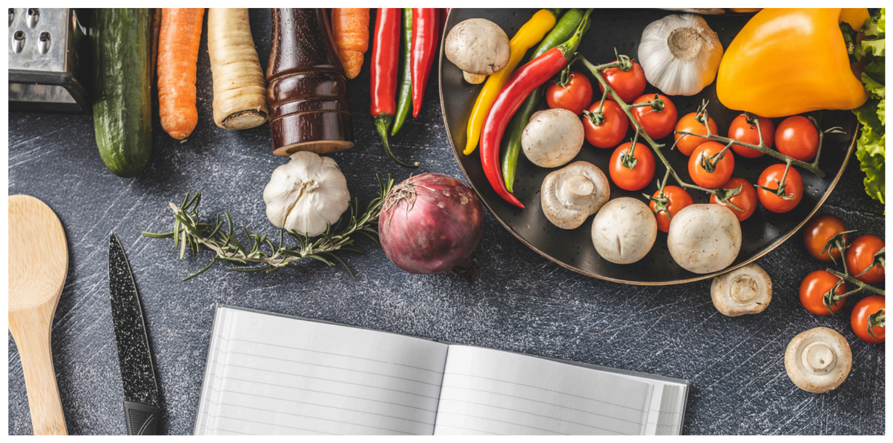

This section is designed to be used with **Behavioural Call 1**. You do not need to do anything with it until then.

UCL

Introduction to tracking

Why behavioural tracking?

There is lots of evidence in behavioural science that **people who set goals and track (or 'self-monitor') their behaviour are more successful in changing their diet, physical activity and weight.** Tracking is therefore a crucial part of any behavioural support programme.

However, believe it or not, we still don't know the best way to track behaviour, especially diet. There is unlikely to be a 'one size fits all' way, because we are all so different - the main thing is that **it is done in a way that works for you.**

In preparing for this study, we searched for apps and websites that we could recommend for setting dietary goals and tracking behaviour. However, there was no app that was freely available and did what we needed it to do! So, we are reverting back to good old pen and paper for now.

What we are asking you to do

We would like you to try using this diary to record your goals, track your behaviours and report anything that got in the way or really helped. This will be discussed in detail in **Behavioural Call 1**.

We are also asking you to tell us whether or not you like this way of tracking your behaviour and find it useful. If you don't like it, or don't find it useful, we are really interested in this too. We'd like to know what you might prefer instead (e.g. an app, a website, an online form).

If you already use a tracker that works for you we'd encourage you to keep using it and would also love to hear what it is.

Goal Setting and Action Planning

Now it's time to put what you've learned about goal setting into practice by setting some of your own. Think about your 'why' and remember to be specific and realistic, and make sure your goals are relevant to **you**.

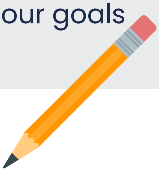

What is my goal for this month?

Why did I choose this goal?

I will start working towards my goal on this date:

Steps I will take towards my goal (action plan):

How confident I feel that I can do this 1=Not at all to 10=Very

Where I will go if I need help:

What I need to get started:

How will I measure my progress along the way?

Challenges I might face:

What I will do if I feel like quitting:

How will I know if I have achieved my goal?

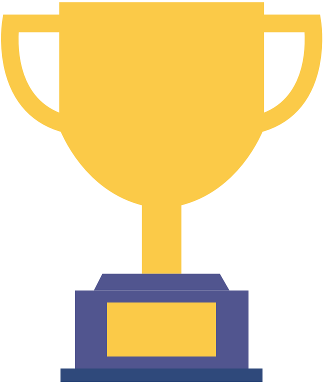

Goal tracking form : Month 1

**Goal 1:**				
**Why it is important:**				
**WEEK 1**	When do you **plan** to do this?	When **did** you do this?	What got in the way or what helped?	Did you troubleshoot?
Mon				
Tues				
Wed				
Thur				
Fri				
Sat				
Sun				
**WEEK 2**				
Mon				
Tues				
Wed				
Thur				
Fri				
Sat				
Sun				

Goal tracking form : Month 1

**Goal 1:**				
**Why it is important:**				
**WEEK 3**	When do you **plan** to do this?	When **did** you do this?	What got in the way or what helped?	Did you troubleshoot?
Mon				
Tues				
Wed				
Thur				
Fri				
Sat				
Sun				
**WEEK 4**				
Mon				
Tues				
Wed				
Thur				
Fri				
Sat				
Sun				

Goal tracking form : Month 1

**Goal 1:**				
**Why it is important:**				
**WEEK 5**	When do you **plan** to do this?	When **did** you do this?	What got in the way or what helped?	Did you troubleshoot?
Mon				
Tues				
Wed				
Thur				
Fri				
Sat				
Sun				

Notes and reflections: Month 1

Reflecting on goals

Take the time to reflect on your goals.

Don't worry if you didn't stick to your goals completely. The key is to remember that you are trying to make sustainable changes, and this means allowing yourself leeway when things get in the way.

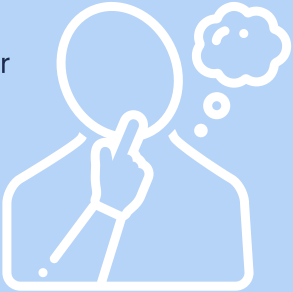

If you know that you have an event/social occasion coming up that will make it hard to achieve your goals, building it into your action plan can make you feel less like you have failed.

If you stuck to a goal, can you make it more challenging next month, or add a new goal?

Think about what made your goal harder or easier to achieve, what can you do more or less of next month.

Reflections on goals

How are you finding this way of tracking ?

Goal Setting and Action Planning

Now it's time to put what you've learned about goal setting into practice by setting some of your own. Think about your 'why' and remember to be specific and realistic, and make sure your goals are relevant to **you**.

What is my goal for this month?

Why did I choose this goal?

I will start working towards my goal on this date:

Steps I will take towards my goal (action plan):

How confident I feel that I can do this 1=Not at all to 10=Very

Where I will go if I need help:

What I need to get started:

How will I measure my progress along the way?

Challenges I might face:

What I will do if I feel like quitting:

How will I know if I have achieved my goal?

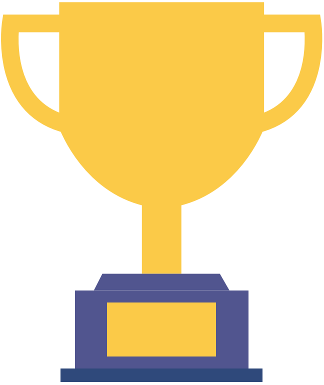

Goal tracking form : Month 2

**Goal 1:**				
**Why it is important:**				
**WEEK 1**	When do you **plan** to do this?	When **did** you do this?	What got in the way or what helped?	Did you troubleshoot?
Mon				
Tues				
Wed				
Thur				
Fri				
Sat				
Sun				
**WEEK 2**				
Mon				
Tues				
Wed				
Thur				
Fri				
Sat				
Sun				

Goal tracking form : Month 2

**Goal 1:**				
**Why it is important:**				
**WEEK 3**	When do you **plan** to do this?	When **did** you do this?	What got in the way or what helped?	Did you troubleshoot?
Mon				
Tues				
Wed				
Thur				
Fri				
Sat				
Sun				
**WEEK 4**				
Mon				
Tues				
Wed				
Thur				
Fri				
Sat				
Sun				

Goal tracking form : Month 2

**Goal 1:**				
**Why it is important:**				
**WEEK 5**	When do you **plan** to do this?	When **did** you do this?	What got in the way or what helped?	Did you troubleshoot?
Mon				
Tues				
Wed				
Thur				
Fri				
Sat				
Sun				

Goal tracking form : Month 2

**Goal 2:**				
**Why it is important:**				
**WEEK 1**	When do you **plan** to do this?	When **did** you do this?	What got in the way or what helped?	Did you troubleshoot?
Mon				
Tues				
Wed				
Thur				
Fri				
Sat				
Sun				
**WEEK 2**				
Mon				
Tues				
Wed				
Thur				
Fri				
Sat				
Sun				

Goal tracking form : Month 2

**Goal 2:**				
**Why it is important:**				
**WEEK 3**	When do you **plan** to do this?	When **did** you do this?	What got in the way or what helped?	Did you troubleshoot?
Mon				
Tues				
Wed				
Thur				
Fri				
Sat				
Sun				
**WEEK 4**				
Mon				
Tues				
Wed				
Thur				
Fri				
Sat				
Sun				

Goal tracking form : Month 2

**Goal 2:**				
**Why it is important:**				
**WEEK 5**	When do you **plan** to do this?	When **did** you do this?	What got in the way or what helped?	Did you troubleshoot?
Mon				
Tues				
Wed				
Thur				
Fri				
Sat				
Sun				

Notes and reflections: Month 2

Reflecting on goals

Take the time to reflect on your goals.

Don't worry if you didn't stick to your goals completely. The key is to remember that you are trying to make sustainable changes, and this means allowing yourself leeway when things get in the way.

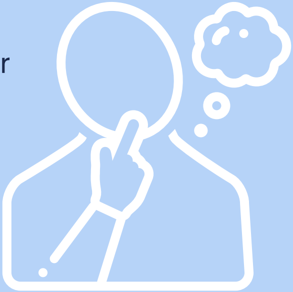

If you know that you have an event/social occasion coming up that will make it hard to achieve your goal, building it into your action plan can make you feel less like you have failed.

If you stuck to a goal, can you make it more challenging next month, or add a new goal?

Think about what made your goal harder or easier to achieve, what can you do more or less of next month?

Reflections on goals

How are you finding this way of tracking ?

Goal Setting and Action Planning

Now it's time to put what you've learned about goal setting into practice by setting some of your own. Think about your 'why' and remember to be specific and realistic, and make sure your goals are relevant to **you**.

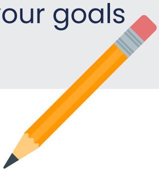

What is my goal for this month?

Why did I choose this goal?

I will start working towards my goal on this date:

Steps I will take towards my goal (action plan):

How confident I feel that I can do this 1=Not at all to 10=Very

Where I will go if I need help:

What I need to get started:

How will I measure my progress along the way?

Challenges I might face:

What I will do if I feel like quitting:

How will I know if I have achieved my goal?

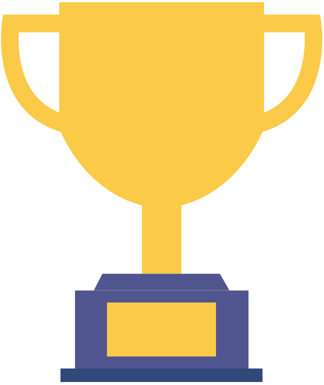

Goal tracking form : Month 3

**Goal 1:**				
**Why it is important:**				
**WEEK 1**	When do you **plan** to do this?	When **did** you do this?	What got in the way or what helped?	Did you troubleshoot?
Mon				
Tues				
Wed				
Thur				
Fri				
Sat				
Sun				
**WEEK 2**				
Mon				
Tues				
Wed				
Thur				
Fri				
Sat				
Sun				

Goal tracking form : Month 3

**Goal 1:**				
**Why it is important:**				
**WEEK 3**	When do you **plan** to do this?	When **did** you do this?	What got in the way or what helped?	Did you troubleshoot?
Mon				
Tues				
Wed				
Thur				
Fri				
Sat				
Sun				
**WEEK 4**				
Mon				
Tues				
Wed				
Thur				
Fri				
Sat				
Sun				

Goal tracking form : Month 3

**Goal 1:**				
**Why it is important:**				
**WEEK 5**	When do you **plan** to do this?	When **did** you do this?	What got in the way or what helped?	Did you troubleshoot?
Mon				
Tues				
Wed				
Thur				
Fri				
Sat				
Sun				

Goal tracking form : Month 3

**Goal 2:**				
**Why it is important:**				
**WEEK 1**	When do you **plan** to do this?	When **did** you do this?	What got in the way or what helped?	Did you troubleshoot?
Mon				
Tues				
Wed				
Thur				
Fri				
Sat				
Sun				
**WEEK 2**				
Mon				
Tues				
Wed				
Thur				
Fri				
Sat				
Sun				

Goal tracking form : Month 3

**Goal 2:**				
**Why it is important:**				
**WEEK 3**	When do you **plan** to do this?	When **did** you do this?	What got in the way or what helped?	Did you troubleshoot?
Mon				
Tues				
Wed				
Thur				
Fri				
Sat				
Sun				
**WEEK 4**				
Mon				
Tues				
Wed				
Thur				
Fri				
Sat				
Sun				

Goal tracking form : Month 3

**Goal 2:**				
**Why it is important:**				
**WEEK 5**	When do you **plan** to do this?	When **did** you do this?	What got in the way or what helped?	Did you troubleshoot?
Mon				
Tues				
Wed				
Thur				
Fri				
Sat				
Sun				

Goal tracking form : Month 3

**Goal 3:**				
**Why it is important:**				
**WEEK 1**	When do you **plan** to do this?	When **did** you do this?	What got in the way or what helped?	Did you troubleshoot?
Mon				
Tues				
Wed				
Thur				
Fri				
Sat				
Sun				
**WEEK 2**				
Mon				
Tues				
Wed				
Thur				
Fri				
Sat				
Sun				

Goal tracking form : Month 3

**Goal 3:**				
**Why it is important:**				
**WEEK 3**	When do you **plan** to do this?	When **did** you do this?	What got in the way or what helped?	Did you troubleshoot?
Mon				
Tues				
Wed				
Thur				
Fri				
Sat				
Sun				
**WEEK 4**				
Mon				
Tues				
Wed				
Thur				
Fri				
Sat				
Sun				

Goal tracking form : Month 3

**Goal 3:**				
**Why it is important:**				
**WEEK 5**	When do you **plan** to do this?	When **did** you do this?	What got in the way or what helped?	Did you troubleshoot?
Mon				
Tues				
Wed				
Thur				
Fri				
Sat				
Sun				

Notes and reflections: Month 3

Reflecting on goals

Take the time to reflect on your goals.

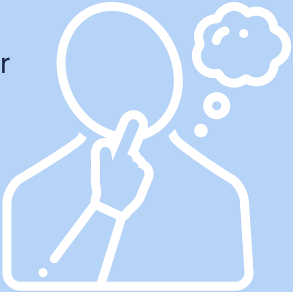

Don't worry if you didn't stick to your goals completely. The key is to remember that you are trying to make sustainable changes, and this means allowing yourself leeway when things get in the way.

If you know that you have an event/social occasion coming up that will make it hard to achieve your goals, building it into your action plan can make you feel less like you have failed.

If you stuck to a goal, can you make it more challenging next month, or add a new goal?

Think about what made your goal harder or easier to achieve, what can you do more or less of next month?

Reflections on goals

How are you finding this way of tracking ?

Goal Setting and Action Planning

Now it's time to put what you've learned about goal setting into practice by setting some of your own. Think about your 'why' and remember to be specific and realistic, and make sure your goals are relevant to **you**.

What is my goal for this month?

Why did I choose this goal?

I will start working towards my goal on this date:

Steps I will take towards my goal (action plan):

How confident I feel that I can do this 1=Not at all to 10=Very

Where I will go if I need help:

What I need to get started:

How will I measure my progress along the way?

Challenges I might face:

What I will do if I feel like quitting:

How will I know if I have achieved my goal?

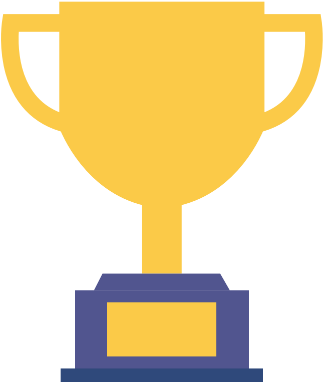

Goal tracking form : Month 4

**Goal 1:**				
**Why it is important:**				
**WEEK 1**	When do you **plan** to do this?	When **did** you do this?	What got in the way or what helped?	Did you troubleshoot?
Mon				
Tues				
Wed				
Thur				
Fri				
Sat				
Sun				
**WEEK 2**				
Mon				
Tues				
Wed				
Thur				
Fri				
Sat				
Sun				

Goal tracking form : Month 4

**Goal 1:**				
**Why it is important:**				
**WEEK 3**	When do you **plan** to do this?	When **did** you do this?	What got in the way or what helped?	Did you troubleshoot?
Mon				
Tues				
Wed				
Thur				
Fri				
Sat				
Sun				
**WEEK 4**				
Mon				
Tues				
Wed				
Thur				
Fri				
Sat				
Sun				

Goal tracking form : Month 4

**Goal 1:**				
**Why it is important:**				
**WEEK 5**	When do you **plan** to do this?	When **did** you do this?	What got in the way or what helped?	Did you troubleshoot?
Mon				
Tues				
Wed				
Thur				
Fri				
Sat				
Sun				

Goal tracking form : Month 4

**Goal 2:**				
**Why it is important:**				
**WEEK 1**	When do you **plan** to do this?	When **did** you do this?	What got in the way or what helped?	Did you troubleshoot?
Mon				
Tues				
Wed				
Thur				
Fri				
Sat				
Sun				
**WEEK 2**				
Mon				
Tues				
Wed				
Thur				
Fri				
Sat				
Sun				

Goal tracking form : Month 4

**Goal 2:**				
**Why it is important:**				
**WEEK 3**	When do you **plan** to do this?	When **did** you do this?	What got in the way or what helped?	Did you troubleshoot?
Mon				
Tues				
Wed				
Thur				
Fri				
Sat				
Sun				
**WEEK 4**				
Mon				
Tues				
Wed				
Thur				
Fri				
Sat				
Sun				

Goal tracking form : Month 4

**Goal 2:**				
**Why it is important:**				
**WEEK 5**	When do you **plan** to do this?	When **did** you do this?	What got in the way or what helped?	Did you troubleshoot?
Mon				
Tues				
Wed				
Thur				
Fri				
Sat				
Sun				

Goal tracking form : Month 4

**Goal 3:**				
**Why it is important:**				
**WEEK 1**	When do you **plan** to do this?	When **did** you do this?	What got in the way or what helped?	Did you troubleshoot?
Mon				
Tues				
Wed				
Thur				
Fri				
Sat				
Sun				
**WEEK 2**				
Mon				
Tues				
Wed				
Thur				
Fri				
Sat				
Sun				

Goal tracking form : Month 4

**Goal 3:**				
**Why it is important:**				
**WEEK 3**	When do you **plan** to do this?	When **did** you do this?	What got in the way or what helped?	Did you troubleshoot?
Mon				
Tues				
Wed				
Thur				
Fri				
Sat				
Sun				
**WEEK 4**				
Mon				
Tues				
Wed				
Thur				
Fri				
Sat				
Sun				

Goal tracking form : Month 4

**Goal 3:**				
**Why it is important:**				
**WEEK 5**	When do you **plan** to do this?	When **did** you do this?	What got in the way or what helped?	Did you troubleshoot?
Mon				
Tues				
Wed				
Thur				
Fri				
Sat				
Sun				

Goal tracking form : Month 4

**Goal 4:**				
**Why it is important:**				
**WEEK 1**	When do you **plan** to do this?	When **did** you do this?	What got in the way or what helped?	Did you troubleshoot?
Mon				
Tues				
Wed				
Thur				
Fri				
Sat				
Sun				
**WEEK 2**				
Mon				
Tues				
Wed				
Thur				
Fri				
Sat				
Sun				

Goal tracking form : Month 4

**Goal 4:**				
**Why it is important:**				
**WEEK 3**	When do you **plan** to do this?	When **did** you do this?	What got in the way or what helped?	Did you troubleshoot?
Mon				
Tues				
Wed				
Thur				
Fri				
Sat				
Sun				
**WEEK 4**				
Mon				
Tues				
Wed				
Thur				
Fri				
Sat				
Sun				

Goal tracking form : Month 4

**Goal 4:**				
**Why it is important:**				
**WEEK 5**	When do you **plan** to do this?	When **did** you do this?	What got in the way or what helped?	Did you troubleshoot?
Mon				
Tues				
Wed				
Thur				
Fri				
Sat				
Sun				

Notes and reflections: Month 4

Reflecting on goals

Take the time to reflect on your goals.

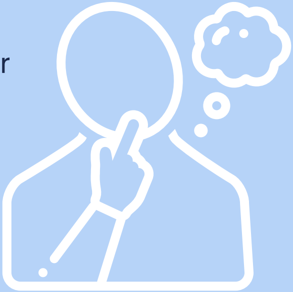

Don't worry if you didn't stick to your goals completely. The key is to remember that you are trying to make sustainable changes, and this means allowing yourself leeway when things get in the way.

If you know that you have an event/social occasion coming up that will make it hard to still achieve your goal, building it into your action plan can make you feel less like you have failed.

If you stuck to a goal, can you make it more challenging next month, or add a new goal?

Think about what made your goal harder or easier to achieve, what can you do more or less of next month?

Reflections on goals

How are you finding this way of tracking ?

Goal Setting and Action Planning

Now it's time to put what you've learned about goal setting into practice by setting some of your own. Think about your 'why' and remember to be specific and realistic, and make sure your goals are relevant to **you**.

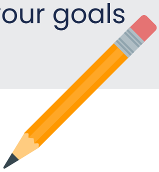

What is my goal for this month?

Why did I choose this goal?

I will start working towards my goal on this date:

Steps I will take towards my goal (action plan):

How confident I feel that I can do this 1=Not at all to 10=Very

Where I will go if I need help:

What I need to get started:

How will I measure my progress along the way?

Challenges I might face:

What I will do if I feel like quitting:

How will I know if I have achieved my goal?

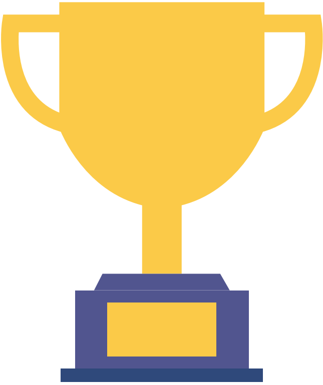

Goal tracking form : Month 5

**Goal 1:**				
**Why it is important:**				
**WEEK 1**	When do you **plan** to do this?	When **did** you do this?	What got in the way or what helped?	Did you troubleshoot?
Mon				
Tues				
Wed				
Thur				
Fri				
Sat				
Sun				
**WEEK 2**				
Mon				
Tues				
Wed				
Thur				
Fri				
Sat				
Sun				

Goal tracking form : Month 5

**Goal 1:**				
**Why it is important:**				
**WEEK 3**	When do you **plan** to do this?	When **did** you do this?	What got in the way or what helped?	Did you troubleshoot?
Mon				
Tues				
Wed				
Thur				
Fri				
Sat				
Sun				
**WEEK 4**				
Mon				
Tues				
Wed				
Thur				
Fri				
Sat				
Sun				

Goal tracking form : Month 5

**Goal 1:**				
**Why it is important:**				
**WEEK 5**	When do you **plan** to do this?	When **did** you do this?	What got in the way or what helped?	Did you troubleshoot?
Mon				
Tues				
Wed				
Thur				
Fri				
Sat				
Sun				

Goal tracking form : Month 5

**Goal 2:**				
**Why it is important:**				
**WEEK 1**	When do you **plan** to do this?	When **did** you do this?	What got in the way or what helped?	Did you troubleshoot?
Mon				
Tues				
Wed				
Thur				
Fri				
Sat				
Sun				
**WEEK 2**				
Mon				
Tues				
Wed				
Thur				
Fri				
Sat				
Sun				

Goal tracking form : Month 5

**Goal 1:**				
**Why it is important:**				
**WEEK 3**	When do you **plan** to do this?	When **did** you do this?	What got in the way or what helped?	Did you troubleshoot?
Mon				
Tues				
Wed				
Thur				
Fri				
Sat				
Sun				
**WEEK 4**				
Mon				
Tues				
Wed				
Thur				
Fri				
Sat				
Sun				

Goal tracking form : Month 5

**Goal 2:**				
**Why it is important:**				
**WEEK 5**	When do you **plan** to do this?	When **did** you do this?	What got in the way or what helped?	Did you troubleshoot?
Mon				
Tues				
Wed				
Thur				
Fri				
Sat				
Sun				

Goal tracking form : Month 5

**Goal 3:**				
**Why it is important:**				
**WEEK 1**	When do you **plan** to do this?	When **did** you do this?	What got in the way or what helped?	Did you troubleshoot?
Mon				
Tues				
Wed				
Thur				
Fri				
Sat				
Sun				
**WEEK 2**				
Mon				
Tues				
Wed				
Thur				
Fri				
Sat				
Sun				

Goal tracking form : Month 5

**Goal 3:**				
**Why it is important:**				
**WEEK 3**	When do you **plan** to do this?	When **did** you do this?	What got in the way or what helped?	Did you troubleshoot?
Mon				
Tues				
Wed				
Thur				
Fri				
Sat				
Sun				
**WEEK 4**				
Mon				
Tues				
Wed				
Thur				
Fri				
Sat				
Sun				

Goal tracking form : Month 5

**Goal 3:**				
**Why it is important:**				
**WEEK 5**	When do you **plan** to do this?	When **did** you do this?	What got in the way or what helped?	Did you troubleshoot?
Mon				
Tues				
Wed				
Thur				
Fri				
Sat				
Sun				

Goal tracking form : Month 5

**Goal 4:**				
**Why it is important:**				
**WEEK 1**	When do you **plan** to do this?	When **did** you do this?	What got in the way or what helped?	Did you troubleshoot?
Mon				
Tues				
Wed				
Thur				
Fri				
Sat				
Sun				
**WEEK 2**				
Mon				
Tues				
Wed				
Thur				
Fri				
Sat				
Sun				

Goal tracking form : Month 5

**Goal 4:**				
**Why it is important:**				
**WEEK 3**	When do you **plan** to do this?	When **did** you do this?	What got in the way or what helped?	Did you troubleshoot?
Mon				
Tues				
Wed				
Thur				
Fri				
Sat				
Sun				
**WEEK 4**				
Mon				
Tues				
Wed				
Thur				
Fri				
Sat				
Sun				

Goal tracking form : Month 5

**Goal 4:**				
**Why it is important:**				
**WEEK 5**	When do you **plan** to do this?	When **did** you do this?	What got in the way or what helped?	Did you troubleshoot?
Mon				
Tues				
Wed				
Thur				
Fri				
Sat				
Sun				

Goal tracking form : Month 5

**Goal 5:**				
**Why it is important:**				
**WEEK 1**	When do you **plan** to do this?	When **did** you do this?	What got in the way or what helped?	Did you troubleshoot?
Mon				
Tues				
Wed				
Thur				
Fri				
Sat				
Sun				
**WEEK 2**				
Mon				
Tues				
Wed				
Thur				
Fri				
Sat				
Sun				

Goal tracking form : Month 5

**Goal 5:**				
**Why it is important:**				
**WEEK 3**	When do you **plan** to do this?	When **did** you do this?	What got in the way or what helped?	Did you troubleshoot?
Mon				
Tues				
Wed				
Thur				
Fri				
Sat				
Sun				
**WEEK 4**				
Mon				
Tues				
Wed				
Thur				
Fri				
Sat				
Sun				

Goal tracking form : Month 5

**Goal 5:**				
**Why it is important:**				
**WEEK 5**	When do you **plan** to do this?	When **did** you do this?	What got in the way or what helped?	Did you troubleshoot?
Mon				
Tues				
Wed				
Thur				
Fri				
Sat				
Sun				

Notes and reflections: Month 5

Reflecting on goals

Take the time to reflect on your goals.

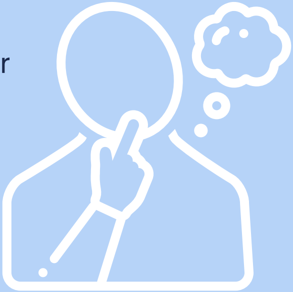

Don't worry if you didn't stick to your goals completely. The key is to remember that you are trying to make sustainable changes, and this means allowing yourself leeway when things get in the way.

If you know that you have an event/social occasion coming up that will make it hard to achieve your goals, building it into your action plan can make you feel less like you have failed.

If you stuck to your goal, can you make it more challenging next month, or add a new goal?

Think about what made your goal harder or easier to achieve, what can you do more or less of next month?

Reflections on goals

How are you finding this way of tracking ?

SECTION 3: PHYSICAL ACTIVITY TRACKING

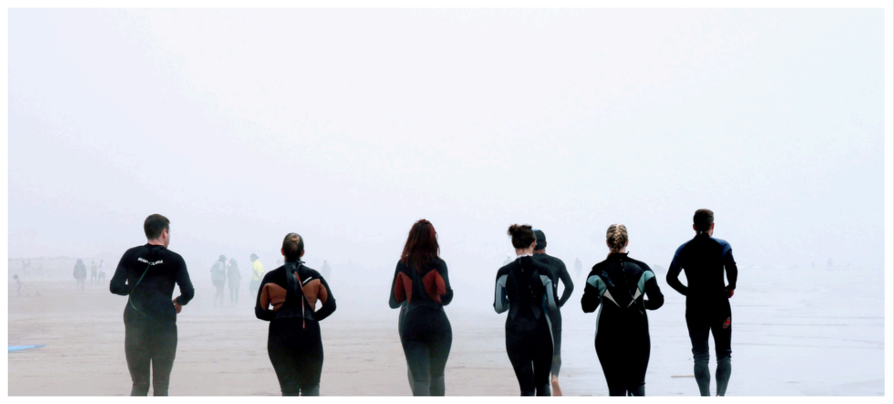

This section is designed to be used with **Behavioural Call 3**. You do not need to do anything with this section until you have had this call with the behavioural scientist.

Goal Setting and Action Planning

Now it's time to put what you've learned about goal setting into practice by setting some of your own. Think about your 'why' and remember to be specific and realistic, and make sure your goals are relevant to **you**.

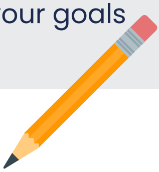

What is my physical activity goal for this month?

Why did I choose this goal?

I will start working towards my goal on this date:

Steps I will take towards my goal (action plan):

How confident I feel that I can do this 1=Not at all to 10=Very

Where I will go if I need help:

What I need to get started:

How will I measure my progress along the way?

....
....
....
....
....
....
....

Challenges I might face:

....
....
....
....
....
....

What I will do if I feel like quitting:

....
....
....
....
....
....

How will I know if I have achieved my goal?

....
....

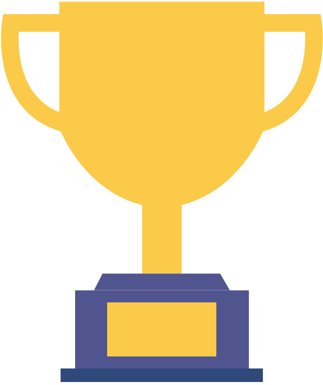

Goal tracking form : Month 3

**Goal 1:**				
**Why it is important:**				
**WEEK 1**	**Where and when** do you plan to do this?	When **did** you do this?	What got in the way or what helped?	Did you troubleshoot?
Mon				
Tues				
Wed				
Thur				
Fri				
Sat				
Sun				
**WEEK 2**				
Mon				
Tues				
Wed				
Thur				
Fri				
Sat				
Sun				

Goal tracking form : Month 3

**Goal 1:**				
**Why it is important:**				
**WEEK 3**	**Where and when** do you plan to do this?	When **did** you do this?	What got in the way or what helped?	Did you troubleshoot?
Mon				
Tues				
Wed				
Thur				
Fri				
Sat				
Sun				
**WEEK 4**				
Mon				
Tues				
Wed				
Thur				
Fri				
Sat				
Sun				

Goal tracking form : Month 3

**Goal 1:**				
**Why it is important:**				
**WEEK 5**	**Where and when** do you plan to do this?	When **did** you do this?	What got in the way or what helped?	Did you troubleshoot?
Mon				
Tues				
Wed				
Thur				
Fri				
Sat				
Sun				

Notes and reflections: Month 3

Reflecting on physical activity goals

Take the time to reflect on your goals.

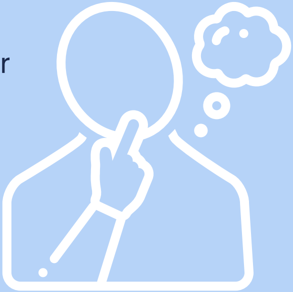

Don't worry if you didn't stick to your goals completely. The key is to remember that you are trying to make sustainable changes, and this means allowing yourself leeway when things get in the way.

If you know that you have an event/social occasion coming up that will make it hard to achieve your goals, building it into your action plan can make you feel less like you have failed.

If you stuck to a goal, can you make it more challenging next month, or add a new goal?

Think about what made your goal harder or easier to achieve, what can you do more or less of next month?

Reflections on physical activity goals

How are you finding this way of tracking ?

Goal Setting and Action Planning

Now it's time to put what you've learned about goal setting into practice by setting some of your own. Think about your 'why' and remember to be specific and realistic, and make sure your goals are relevant to **you**.

What is my physical activity goal for this month?

Why did I choose this goal?

I will start working towards my goal on this date:

Steps I will take towards my goal (action plan):

How confident I feel that I can do this 1=Not at all to 10=Very

Where I will go if I need help:

What I need to get started:

How will I measure my progress along the way?

Challenges I might face:

What I will do if I feel like quitting:

How will I know if I have achieved my goal?

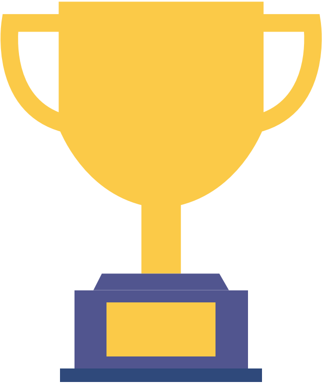

Goal tracking form : Month 4

**Goal 1:**				
**Why it is important:**				
**WEEK 1**	**Where and when** do you plan to do this?	When **did** you do this?	What got in the way or what helped?	Did you troubleshoot?
Mon				
Tues				
Wed				
Thur				
Fri				
Sat				
Sun				
**WEEK 2**				
Mon				
Tues				
Wed				
Thur				
Fri				
Sat				
Sun				

Goal tracking form : Month 3

**Goal 1:**				
**Why it is important:**				
**WEEK 3**	**Where & when** do you plan to do this?	When **did** you do this?	What got in the way or what helped?	Did you troubleshoot?
Mon				
Tues				
Wed				
Thur				
Fri				
Sat				
Sun				
**WEEK 4**				
Mon				
Tues				
Wed				
Thur				
Fri				
Sat				
Sun				

Goal tracking form : Month 4

**Goal 1:**				
**Why it is important:**				
**WEEK 5**	**Where and when** do you plan to do this?	When **did** you do this?	What got in the way or what helped?	Did you troubleshoot?
Mon				
Tues				
Wed				
Thur				
Fri				
Sat				
Sun				

Goal tracking form : Month 4

**Goal 2:**				
**Why it is important:**				
**WEEK 1**	**Where and when** do you plan to do this?	When **did** you do this?	What got in the way or what helped?	Did you troubleshoot?
Mon				
Tues				
Wed				
Thur				
Fri				
Sat				
Sun				
**WEEK 2**				
Mon				
Tues				
Wed				
Thur				
Fri				
Sat				
Sun				

Goal tracking form : Month 3

**Goal 2:**				
**Why it is important:**				
**WEEK 3**	**Where & when** do you plan to do this?	When **did** you do this?	What got in the way or what helped?	Did you troubleshoot?
Mon				
Tues				
Wed				
Thur				
Fri				
Sat				
Sun				
**WEEK 4**				
Mon				
Tues				
Wed				
Thur				
Fri				
Sat				
Sun				

Goal tracking form : Month 4

**Goal 2:**				
**Why it is important:**				
**WEEK 5**	**Where and when** do you plan to do this?	When **did** you do this?	What got in the way or what helped?	Did you troubleshoot?
Mon				
Tues				
Wed				
Thur				
Fri				
Sat				
Sun				

Notes and reflections: Month 4

Reflecting on physical activity goals

Take the time to reflect on your goals.

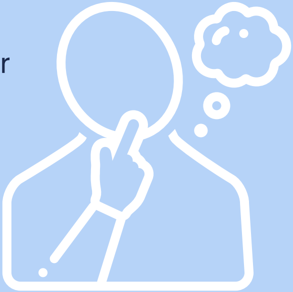

Don't worry if you didn't stick to your goals completely. The key is to remember that you are trying to make sustainable changes, and this means allowing yourself leeway when things get in the way.

If you know that you have an event/social occasion coming up that will make it hard to achieve your goals, building it into your action plan can make you feel less like you have failed.

If you stuck to a goal, can you make it more challenging next month, or add a new goal?

Think about what made your goal harder or easier to achieve, what can you do more or less of next month?

Reflections on physical activity goals

How are you finding this way of tracking ?

Goal Setting and Action Planning

Now it's time to put what you've learned about goal setting into practice by setting some of your own. Think about your 'why' and remember to be specific and realistic, and make sure your goals are relevant to **you**.

What is my physical activity goal for this month?

Why did I choose this goal?

I will start working towards my goal on this date:

Steps I will take towards my goal (action plan):

How confident I feel that I can do this 1=Not at all to 10=Very

Where I will go if I need help:

What I need to get started:

How will I measure my progress along the way?

Challenges I might face:

What I will do if I feel like quitting:

How will I know if I have achieved my goal?

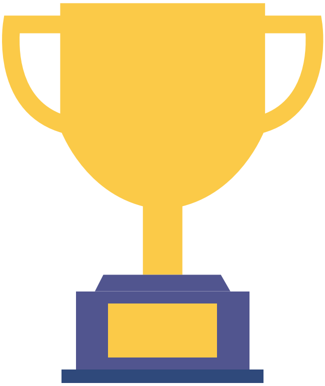

Goal tracking form : Month 5

**Goal 1:**				
**Why it is important:**				
**WEEK 1**	**Where and when** do you plan to do this?	When **did** you do this?	What got in the way or what helped?	Did you troubleshoot?
Mon				
Tues				
Wed				
Thur				
Fri				
Sat				
Sun				
**WEEK 2**				
Mon				
Tues				
Wed				
Thur				
Fri				
Sat				
Sun				

Goal tracking form : Month 4

**Goal 1:**				
**Why it is important:**				
**WEEK 3**	**Where & when** do you plan to do this?	When **did** you do this?	What got in the way or what helped?	Did you troubleshoot?
Mon				
Tues				
Wed				
Thur				
Fri				
Sat				
Sun				
**WEEK 4**				
Mon				
Tues				
Wed				
Thur				
Fri				
Sat				
Sun				

Goal tracking form : Month 5

**Goal 1:**				
**Why it is important:**				
**WEEK 5**	**Where and when** do you plan to do this?	When **did** you do this?	What got in the way or what helped?	Did you troubleshoot?
Mon				
Tues				
Wed				
Thur				
Fri				
Sat				
Sun				

Goal tracking form : Month 5

**Goal 2:**				
**Why it is important:**				
**WEEK 1**	**Where and when** do you plan to do this?	When **did** you do this?	What got in the way or what helped?	Did you troubleshoot?
Mon				
Tues				
Wed				
Thur				
Fri				
Sat				
Sun				
**WEEK 2**				
Mon				
Tues				
Wed				
Thur				
Fri				
Sat				
Sun				

Goal tracking form : Month 5

**Goal 2:**				
**Why it is important:**				
**WEEK 3**	**Where & when** do you plan to do this?	When **did** you do this?	What got in the way or what helped?	Did you troubleshoot?
Mon				
Tues				
Wed				
Thur				
Fri				
Sat				
Sun				
**WEEK 4**				
Mon				
Tues				
Wed				
Thur				
Fri				
Sat				
Sun				

Goal tracking form : Month 5

**Goal 2:**				
**Why it is important:**				
**WEEK 5**	**Where and when** do you plan to do this?	When **did** you do this?	What got in the way or what helped?	Did you troubleshoot?
Mon				
Tues				
Wed				
Thur				
Fri				
Sat				
Sun				

Goal tracking form : Month 5

**Goal 3:**				
**Why it is important:**				
**WEEK 1**	**Where and when** do you plan to do this?	When **did** you do this?	What got in the way or what helped?	Did you troubleshoot?
Mon				
Tues				
Wed				
Thur				
Fri				
Sat				
Sun				
**WEEK 2**				
Mon				
Tues				
Wed				
Thur				
Fri				
Sat				
Sun				

Goal tracking form : Month 5

**Goal 3:**				
**Why it is important:**				
**WEEK 3**	**Where & when** do you plan to do this?	When **did** you do this?	What got in the way or what helped?	Did you troubleshoot?
Mon				
Tues				
Wed				
Thur				
Fri				
Sat				
Sun				
**WEEK 4**				
Mon				
Tues				
Wed				
Thur				
Fri				
Sat				
Sun				

Goal tracking form : Month 5

**Goal 3:**				
**Why it is important:**				
**WEEK 5**	**Where and when** do you plan to do this?	When **did** you do this?	What got in the way or what helped?	Did you troubleshoot?
Mon				
Tues				
Wed				
Thur				
Fri				
Sat				
Sun				

Notes and reflections: Month 5

Reflecting on physical activity goals

Take the time to reflect on your goals.

Don't worry if you didn't stick to your goals completely. The key is to remember that you are trying to make sustainable changes, and this means allowing yourself leeway when things get in the way.

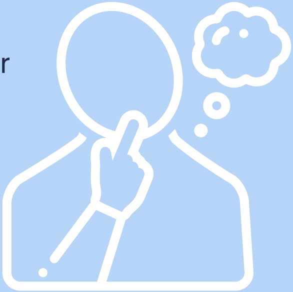

If you know that you have an event/social occasion coming up that will make it hard to achieve your goals, building it into your action plan can make you feel less like you have failed.

If you stuck to your goal, can you make it more challenging next month, or add a new goal?

Think about what made your goal harder or easier to achieve, what can you do more or less of next month?

Reflections on physical activity goals

How are you finding this way of tracking ?

UPDATE

CONTACTS

If you need to book or rearrange any of your calls, please email [redacted] on [redacted]

If you would like to talk about any other aspect of the Behavioural Support Programme, please contact [redacted] on [redacted]

UCL
