# Supplementary material for: Development, content and planned evaluation of a behavioural support intervention to reduce ultraprocessed food intake and increase physical activity in UK healthcare workers: UPDATE trial stage 2 study protocol
Source: BMJ Open. 2025 Oct 29;15(10):e107435. doi: 10.1136/bmjopen-2025-107435 (PMC12574385; doi:10.1136/bmjopen-2025-107435)
Supplement: online supplemental file 3 [file bmjopen-15-10-s003.pdf]

UPDATE

UPDATE BEHAVIOURAL SUPPORT PROGRAMME

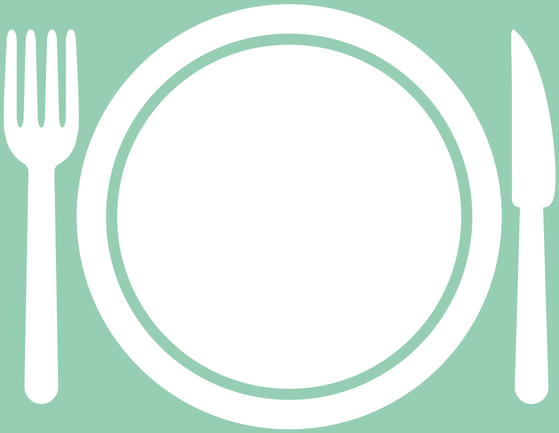

This booklet provides tools to support you to reduce how much ultra-processed food (UPF) you eat, increase your intake of minimally processed foods (MPF), and help you to move more.

It is designed to be used alongside the first **UPDATE Behavioural Support call**, so you don't need to read it before you've had the call.

UCL

Please contact us if you have any accessibility requirements or would like a large-print version of this booklet

Welcome

Who we are

The study is led by [redacted]

The **Behavioural Science** team are [redacted]

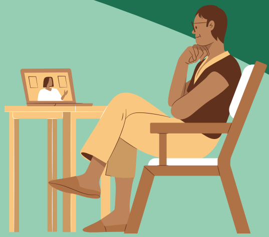

This guide is designed to be used with your UPDATE behavioural support calls. If you haven't booked this call please email [redacted]

Our promise to you

The tips and tools in the call and this guide are based on what we know from decades of behavioural science research, but are also often things we have tried and found useful ourselves and with our clients. We might suggest products, recipes or resources because we have reviewed them and found them to be high quality and useful.

The behavioural science team who designed this guide have no commercial links that would lead us to recommend one brand or product over another. Our information is based on scientific research and, most importantly, works. It was not possible to add all the references and sources into this short booklet, but these are provided on our website: **findmempf.com**

SCAN ME!

Section 1. Ultra-processed food (UPF)

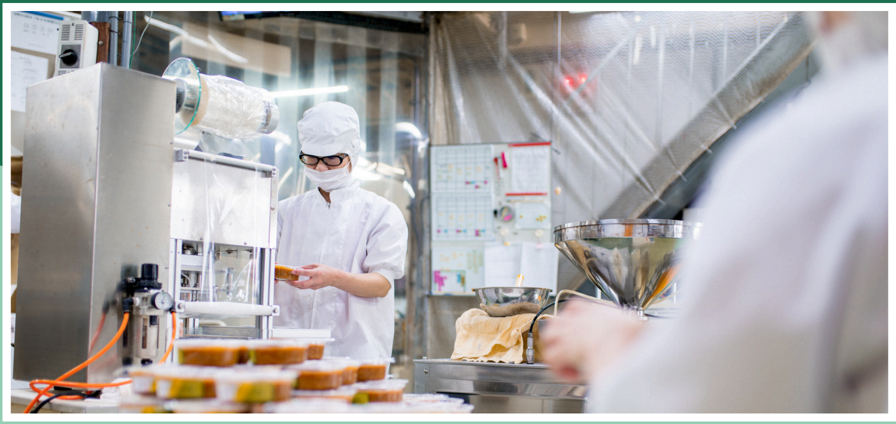

This section explains what ultra-processed foods are, how to recognise them, and the impact they have on our health.

What are ultra-processed foods (UPF)?

NOVA (not an acronym) is a system that splits foods and drinks into four groups:

1. Unprocessed or minimally processed foods (MPF)
2. Processed culinary ingredients
3. Processed foods
4. Ultra-processed foods (UPF)

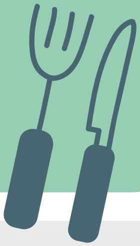

The fourth group, **ultra-processed foods**, refers to products made using **industrial equipment** to break down ingredients, **extract** parts and then recombine them – often with **preservatives**, **flavourings**, and other ingredients not found in most domestic kitchens. UPFs are designed to maximise taste, shelf-life, and profit.

The list of foods in this category is long and includes most:

- Pre-packaged meals (e.g. microwave dinners)
- Packaged bread
- Breakfast cereals
- Sweets, chocolate, biscuits and cakes
- Sausages, chicken nuggets, veggie burgers
- Soft drinks (e.g. cola, fruit flavoured drinks)
- Sweetened yogurt and fromage frais

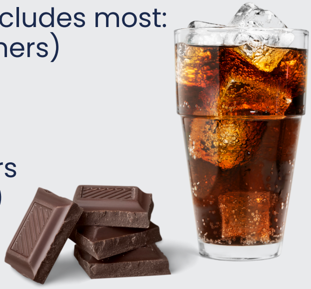

Evidence suggests that UPF might cause us to overeat, and increase our risk of developing health problems.

As part of the 6-month behavioural support programme, we will encourage you to:

- **Reduce how much ultra-processed food you eat**
- **Increase how much unprocessed food you eat.**

The World Cancer Research Fund have produced a very useful guide to help you work out what is ultra-processed [1]. With their permission, we have summarised the key points in this booklet.

NOVA groups 1-3

Now that you know what ultra-processed foods are, you need an introduction to the other three NOVA groups.

You'll be replacing UPF in your diet over the coming months, mainly with items in group 1. Several studies have found replacing UPF with these foods reduces risk of several diseases.

NOVA groups 1-3 also made up one of the diets you've just had as part of the trial.

Later in this booklet you'll find some recipes to help you get started with increasing how much minimally processed food you eat.

1 Unprocessed and minimally processed

Unprocessed and minimally-processed foods (MPF) are foods from nature, including those that have had inedible parts removed and have been dried, crushed, filtered, roasted, or boiled, etc. They contain no added fats, salt, sugar, or other additives, but may be fortified (have added vitamins).

Examples include:

- fruit and vegetables
- eggs, fish and meat
- nuts, seeds and grains
- 100% fruit juice
- pasteurised milk
- natural yoghurt
- dried pulses
- whole cuts of meat

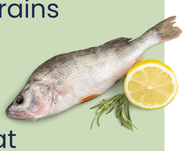

2 Processed culinary ingredients

These are ingredients that are created or extracted from unprocessed and minimally processed foods. They are added to minimally processed foods to make tasty meals.

Examples include:

- fats such as butter or oil
- vinegars
- sugars
- salt
- starches extracted from corn and other plants
- maple syrup

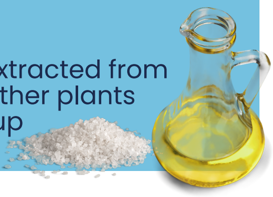

3 Processed foods

This is where a **manufacturer** takes a product from NOVA group 2 – like salt, sugar, oil or vinegar – and adds it to a NOVA group 1 food.

Examples include:

- many tinned goods in oil, brine or syrup, such as fish, fruit and some veg
- foods kept in brine, like olives
- salted nuts
- most cheese, like cheddar and mozzarella
- freshly-made bakery bread

It also includes smoked and cured meats and fish (that haven't been made with reconstituted meat).

What does the science say?

UPF, appetite and weight

One of the first studies to find a link between UPF, appetite and weight gain was conducted in 2019 [2]. Researchers in the United States admitted 20 volunteers to a research centre where they stayed for four weeks. The participants were given a UPF diet for two weeks followed by a minimally processed diet for two weeks (or *vice versa*).

The two diets were matched for calories, protein, fat, carbohydrate, sugar, salt, and fibre, and participants could eat as much or little as they liked.

The results from the study surprised the researchers. They found that:

- People ate 500 calories more per day on the UPF diet than the minimally processed diet (equal to around an extra quarter of an adult's total daily requirements), despite people finding both diets equally pleasant and familiar
- People gained around 1 kg (~2.2 lbs) in weight on the UPF diet and lost 1 kg on the minimally processed diet
- Levels of hormones that reduce appetite were higher during the minimally processed diet compared to before the trial and during the UPF diet.

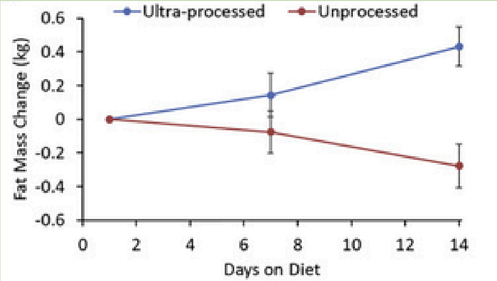

The findings of this study highlighted the need for further research. You've just completed the 'free-living', extended version of the study!

There is emerging evidence that UPFs disrupt the biology and brain processes that control our appetite, so we don't feel as full when we eat these foods, and our brains find them more rewarding and tell us to eat more of them. We think this may be one of the reasons why eating lots of UPF leads to weight gain over time.

What does the science say?

UPF and overall health

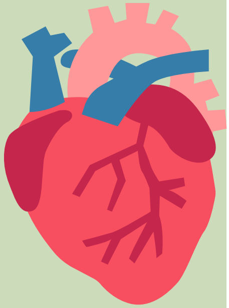

The effects of UPF on health go beyond just weight.

For example, high UPF consumption has been found to increase risk of **heart disease** even when taking body mass index (BMI), physical activity level, alcohol intake, family history of cardiovascular disease, total calorie intake and smoking status into account [3].

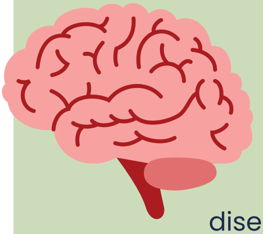

Similarly, higher UPF consumption has been found to be linked with a greater risk of **dementia**, even when accounting for smoking and drinking status, physical activity, BMI, family history of dementia, history of cardiovascular disease, total calorie intake, and healthy diet score (calculated from how much fruit, vegetables, fish, unprocessed red meat, and processed meat people ate) [4].

Additionally, studies have found that people with the highest intake of UPF have increased risk of **early death** compared to those with lower intake of UPF [5].

To note, the average UPF intake of the participants in all of these studies was far lower than the average intake in the UK, which is **over 50% of all calories consumed**.

It isn't all bad news though! Studies have also found that ***replacing UPF in the diet with minimally processed foods is associated with a reduced risk of disease and early death, as well as weight loss***.

You don't have to cut out all UPF, but any reduction you make can help lower your risk, as well as helping you to regulate your weight and appetite.

What are ultra-processed foods (UPF)?

Identifying ultra-processed food

A food is ultra-processed if any of the following common ingredients have been added to it. This is not an exhaustive list, as there are currently **over 10,000 food additives in use globally.**

**Varieties of sugars**	• Fructose<br>• High-fructose corn syrup<br>• Fruit juice concentrates<br>• Invert sugar<br>• Maltodextrin<br>• Dextrose<br>• Lactose (as an added ingredient)
**Modified oils**	• Hydrogenated fats<br>• Interesterified oils
**Proteins**	• Hydrolysed proteins<br>• Soy protein isolate<br>• Gluten (as an added ingredient)<br>• Casein<br>• Whey<br>• Mechanically separated meat
**Additives**	• Flavours and flavour enhancers, **including 'natural flavouring'**<br>• Colours<br>• Emulsifiers and emulsifying agents *e.g. mono- and diglycerides of fatty acids, soy and sunflower lecithin*<br>• Artificial sweeteners<br>• Thickeners and stabilisers *e.g. xantham gum, locust bean gum, cellulose*<br>• Foaming, anti-caking, gelling and glazing agents *e.g. beeswax, carnauba wax*

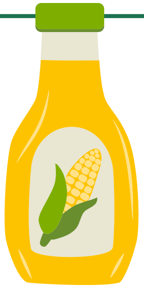

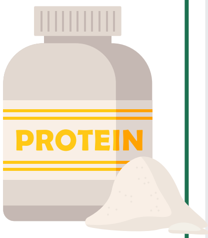

! A food is only UPF if these are listed as separate ingredients on the label. E.g. Milk naturally contains lactose and wheat flour naturally contains gluten but this doesn't make them UPF.

A product might be UPF if...

1. *It is **ready-made** and has more than **5 ingredients***

2. ***It has ingredients you wouldn't find in a domestic kitchen** e.g. emulsifiers, acidity regulators*

3. ***There is a health claim on the package** like 'sugar free', 'reduced fat/sugar', or 'high in protein'*

4. ***The traffic light labels on the packaging are mostly red** (meaning high sugar, salt and fat)*

5. ***It has a long shelf life, but is a supposedly 'fresh' food** – usually means that preservatives have been added*

6. *It is heavily **marketed** or **branded***

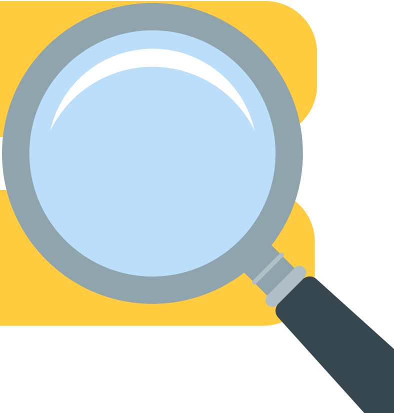

7. ***You are in any doubt!***

Test yourself

Answers at the bottom of the page!

Can you work out which of these products are UPF?

1
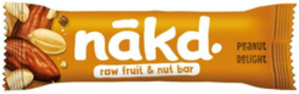

Dates, Peanuts, Sea Salt, a hint of Natural Flavouring

2
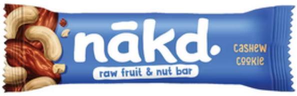

Cashews, dates

3
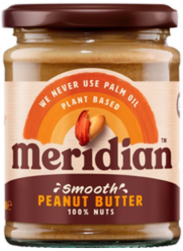

Roasted Peanuts Skins on (100%)

4
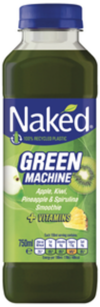

Apple Juice, Water, Banana Puree, Kiwi Puree, Peach Puree, Apple Puree, Pineapple Juice, Mango Puree, Chlorella Powder, Spirulina Powder, Spinach Powder, Natural Flavourings, Vitamins C, E, B6, Thiamine, Riboflavin

5
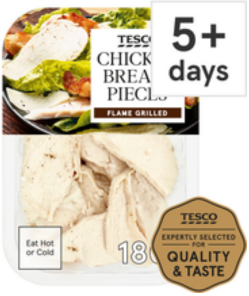

Chicken Breast, Rice Flour, Potato Starch, Cornflour, Stabiliser (Pentasodium Triphosphate), Salt, Rapeseed Oil

6
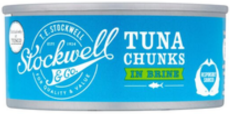

Skipjack Tuna (Fish), Water, Salt

How about these?

7
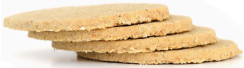

Oatcakes

8
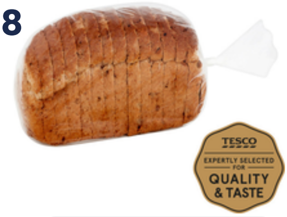

Tesco Bakery Hi Fibre Malted Loaf

9
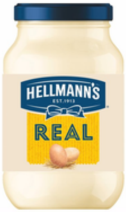

Hellmann's mayonnaise

10
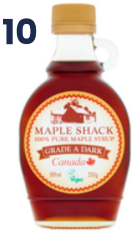

Maple syrup

11
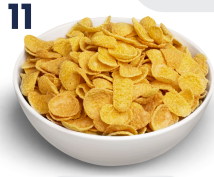

Cornflakes

12
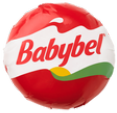

Babybel

13
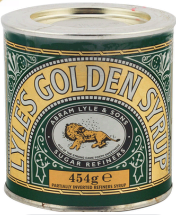

Golden syrup

UPF: 1, 4, 5, 8, 9, 11, 13. Non-UPF: 2, 3, 6, 7, 10, 12.

The Eatwell Guide

The UK government's **Eatwell Guide** shows how much of what we eat should come from each food group to achieve a healthy, balanced diet. The guide gives recommendations on types and amounts of foods and nutrients to eat, but does not give any recommendations on UPF.

Both of the diets you had during the first part of the trial were designed around the Eatwell Guide. Research has shown a link between UPF and worse health outcomes, even when taking overall diet quality or pattern into account. Therefore, this part of the trial set out to test whether two diets that follow the UK government's advice, but with different levels of processing, would result in similar or different effects.

As you reduce your UPF intake over the coming months, it's important to remember to follow the Eatwell Guide. These recommendations are evidence-based and designed to promote health and avoid nutrient deficiencies. **Not every meal we eat needs to be in line with the Eatwell Guide, as long as the balance of your diet is right over the course of the day or week.**

The next few pages will give you some key information on the Eatwell Guide to help you plan what your overall diet might look like.

If you'd like to learn about the Eatwell Guide in more detail, scan the QR code below to visit the NHS Eatwell Guide website.

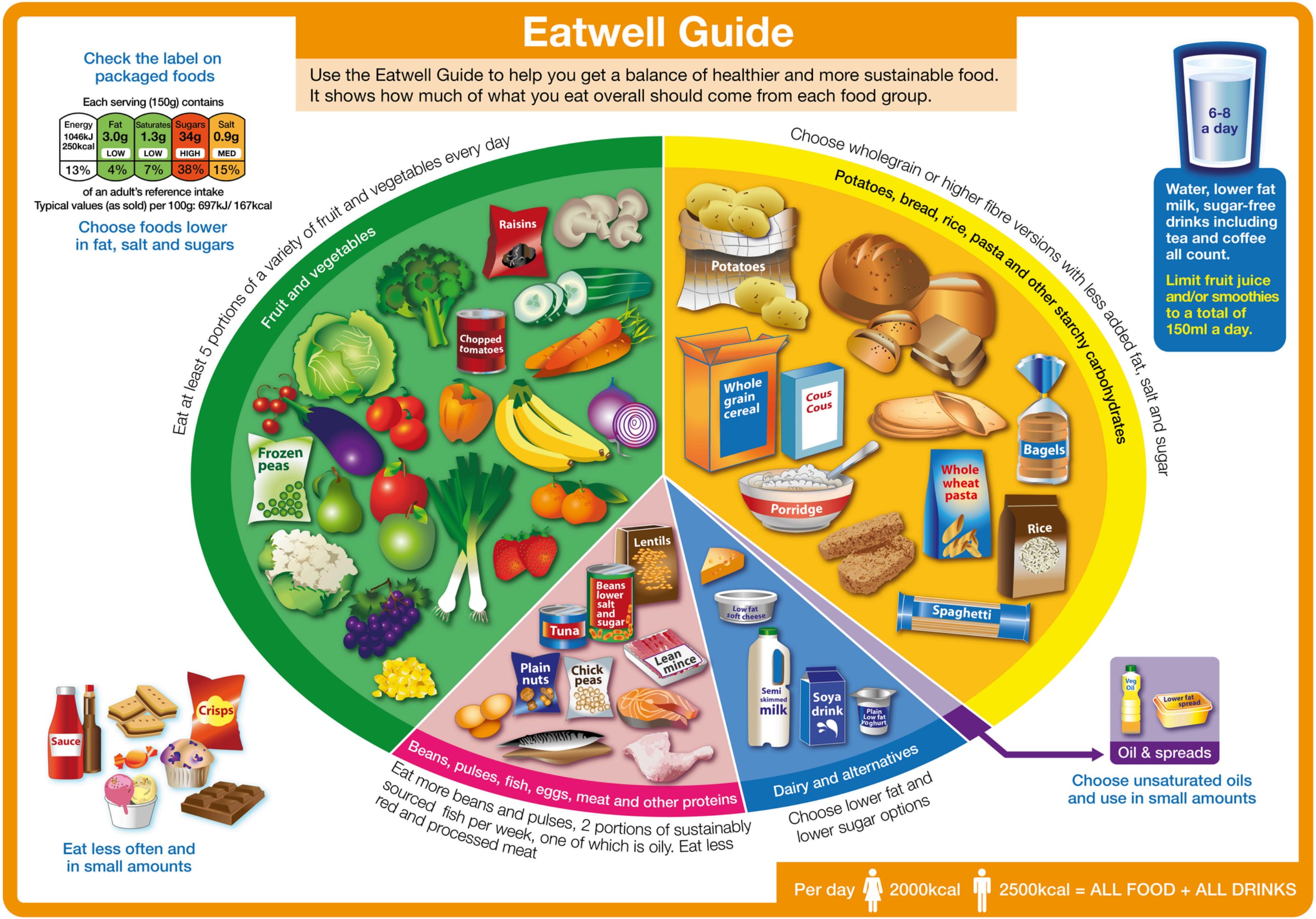

Source: Public Health England in association with the Welsh Government, Food Standards Scotland and the Food Standards Agency in Northern Ireland

© Crown copyright 2016

The Eatwell Guide & foods that aren't UPF

Here's a bit more information from the Eatwell Guide on different food groups, and examples of the team's favourite non-UPF items from these groups. Hopefully you'll see that there are lots of tasty and familiar foods you can enjoy that aren't UPF, whether you decide to try out some exciting new recipes or keep things simple. Feel free to tick any you'd like to try having more of and bring along for your next shop.

Fruit and vegetables

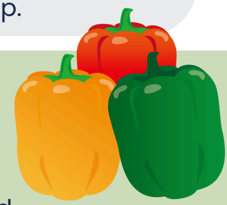

Fruit and vegetables should make up over a third of the food we eat each day. Aim to eat at least 5 portions of a variety of fruit and veg, including fresh, frozen, tinned and dried. Fruit juice and smoothies do count but should be limited to no more than a combined total of 150ml a day.

- ○ aubergine
- ○ avocado
- ○ bananas
- ○ broccoli (fresh/frozen)
- ○ butternut squash
- ○ carrot sticks
- ○ cucumber stick
- ○ frozen mixed berries
- ○ frozen mixed veg
- ○ garlic
- ○ grapes
- ○ green beans
- ○ jackfruit
- ○ kale
- ○ kiwi
- ○ lettuce
- ○ mushrooms
- ○ okra
- ○ onion
- ○ peas
- ○ peppers
- ○ plantain
- ○ rocket
- ○ satsumas
- ○ spinach
- ○ tinned tomatoes
- ○ watermelon

Starchy carbohydrates

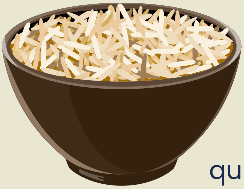

Starchy food should make up just over a third of the food we eat. Healthier examples of starchy foods include wholewheat pasta, brown rice, potatoes with the skin on, oats and whole grains like quinoa, pearl barley, and bulgur wheat.

- ○ bakery bread (not supermarket bakery)
- ○ bulgur wheat
- ○ cassava
- ○ corn
- ○ couscous
- ○ flour
- ○ homemade chips
- ○ homemade flatbread
- ○ homemade popcorn
- ○ homemade wedges
- ○ jacket potato
- ○ mashed potato
- ○ muesli
- ○ oatcakes
- ○ oats
- ○ pasta
- ○ pearl barley
- ○ porridge
- ○ quinoa
- ○ rice
- ○ rice cakes
- ○ rice noodles
- ○ rye bread
- ○ shredded wheat
- ○ spaghetti
- ○ sweet potato

Protein

Beans, pulses, fish, eggs, and meat are good sources of protein, as well as vitamins and minerals. The NHS recommend eating two portions of fish per week, one of which should be oily fish, like salmon, mackerel and sardine, as these also provide important omega-3 fatty acids. Red and processed meat should be limited to 70g per day (around 5 tbsp cooked mince or half a large burger patty), but processed meat should generally be avoided.

Milk, cheese, natural and Greek yoghurt and natural fromage frais are also good sources of protein and some vitamins.

- ○ almond butter
- ○ almonds
- ○ black beans
- ○ chia seeds
- ○ chickpeas
- ○ dried split peas
- ○ edamame
- ○ eggs
- ○ Greek yogurt
- ○ homemade houmous
- ○ kidney beans
- ○ mussels
- ○ peanut butter
- ○ peanuts
- ○ pistachios
- ○ prawns
- ○ quinoa
- ○ roast chicken
- ○ sesame seeds
- ○ sunflower seeds
- ○ tempeh
- ○ tinned lentils
- ○ tinned salmon
- ○ tinned sardines
- ○ tinned tuna
- ○ tofu
- ○ turkey

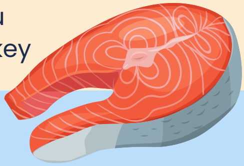

Fats

Choose oils high in unsaturated fats, like rapeseed, sunflower and olive oil for use in cooking and making dressings and marinades.

Generally, we eat too much saturated fat, which is found in some meat and dairy products, coconut oil, and ghee. Swapping saturated fats in the diet for unsaturated fat is recommended to reduce risk of high cholesterol and heart disease, but all types of fat are high in calories, and the Eatwell Guide recommends eating limited amounts.

- ○ extra virgin olive oil
- ○ rapeseed oil
- ○ sunflower oil

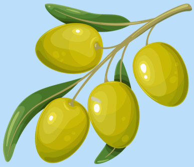

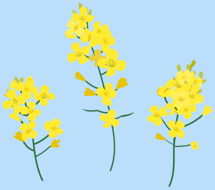

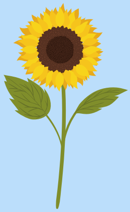

Resources

Podcasts

Episodes

- **Dr Giles Yeo Chews the Fat:** Ultra-processed Food with Chris and Xand Van Tulleken
- **The Psychology Podcast:** Katy Milkman || How to change
- **The Studies Show:** Episode 6: Ultra-processed foods

Series

- **A Thorough Examination with Drs Chris and Xand**
- **Sigma Nutrition**

Programmes & videos

- **What are we feeding our kids?** - BBC documentary, clips can be found on YouTube
- **Panorama: Ultra-Processed Food: A Recipe for Ill Health?** - BBC iPlayer
- **This is an Emergency** - youtu.be/I3U_xd5-SA8
- **Ultra-Processed People: The Science Behind Food That Isn't Food** - youtu.be/FRRGyy5nNlk
- **How to check if food is ultra-processed with Dr. Xand** - bbc.co.uk/programmes/p0g9fp2p

Books

- **Ultra-Processed People,** Chris Van Tulleken
- **Atomic Habits,** James Clear
- **How to Change,** Katy Milkman
- **The Power of Habit,** Charles Duhigg
- **How to Retrain Your Appetite,** Helen McCarthy
- **The Hungry Brain,** Stephan J. Guyenet
- **You are a Badass,** Jen Sincero
- **The Gifts of Imperfection,** Brené Brown

Have a look at the 'Resources' tab on our website for more ideas and links to podcast episodes and videos.

Resources

Website

We have designed a website to help you find non-UPF food options around UCLH and in your local supermarket.

Of course, the best way to reduce how much UPF you eat is preparing your meals and snacks from scratch, but we understand that this isn't always possible, and sometimes you'll need to grab something on the go.

The website also contains some recipes recommended by members of the UPDATE team, and a bit more information about how to spot ultra-processed food.

The website is optimised for mobile use.

Scan the QR code below, or visit **findmempf.com**

Support Group

We plan to introduce an additional element to our behavioural support program - optional monthly live support meetings!

These sessions will take place on Microsoft Teams and will provide the opportunity to share experiences with the team and other people going through the UPDATE programme. This will allow you the chance to share what's working for you, discuss anything you are struggling with, and hear ideas from others going through the same programme.

Although group sessions aren't for everyone (which is why these are entirely optional), there is evidence that support from peers can lead to more effective behaviour change.

We also plan to invite expert speakers to discuss relevant topics such as the importance of reducing ultra-processed foods in your diet and insights from exercise specialists. We will be led by you too - if there are topics that you'd like to hear discussed by experts let us know and we will try and find the right person!

During these meetings, you'll have the opportunity to engage in Q&A sessions, problem-solving discussions, and lively conversations, all hosted by an UPDATE behavioural scientist.

We believe that these live meetings will provide valuable insights and support to enhance your journey to reducing UPF intake.

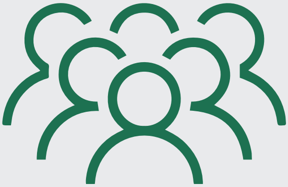

Notes and reflections

How to reduce UPFs

	Already do	Could try
Read ingredients lists/labels to identify UPFs	☐	☐
Cook at home from scratch the majority of the time	☐	☐
Swap fizzy or sugary drinks for unsweetened tea, coffee or water	☐	☐
Swap out UPF snacks for minimally processed options (check out our ideas on page 30)	☐	☐
Bring a packed lunch of minimally processed homemade food into work	☐	☐
Try one new recipe a week using minimally processed ingredients	☐	☐
Anything else you want to add? _________________________________	☐	☐

Section 2. Goal Setting & Action Planning

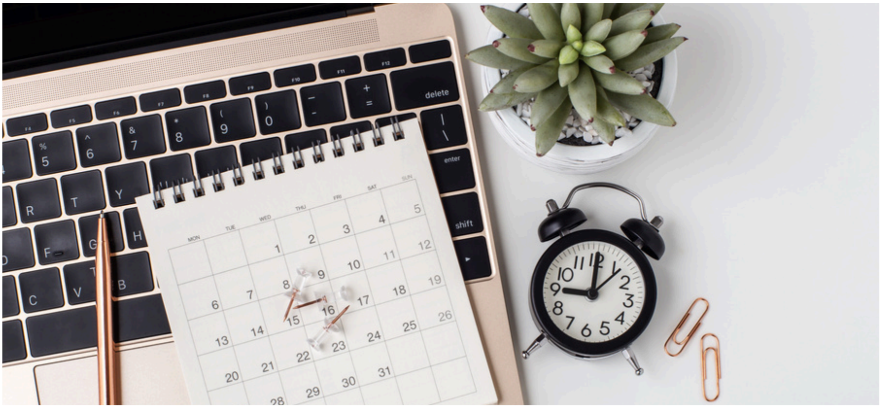

This section introduces some techniques that scientific evidence shows are really important in supporting positive behaviour change.

Goal setting

Why goal setting is important

Research shows that identifying **specific and actionable goals** is important when changing a health behaviour [6, 7].

These goals need to be made into actionable steps to trigger new behaviours and build habits.

Setting **clear**, **specific** and **realistic** goals and working towards them is like creating a roadmap that gives people direction and motivation to make positive changes in their lives.

Setting action plans

Where, when and how will you achieve each goal?

Before the start of each month, we will ask you to write down what you need to do weekly to achieve this goal. Think about where and when you will do these tasks - e.g. you will write a shopping list at home on your day off before you go food shopping.

Can you delegate any of these tasks to someone else?

Sharing your action plans with others will help to keep you accountable.

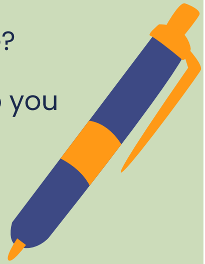

How to set effective goals

Think about what will motivate you to achieve your goals and why. You have a higher chance of achieving a goal if it is **personally motivating**.

Think about how you can enhance your existing skills and what new skills you want to learn - e.g. are you good at cooking and want to learn how to cook more nutritious meals?

Set yourself realistic goals that'll you'll feel proud of achieving. Make sure you're confident that you can achieve this goal. Don't hesitate to modify the goal if you need to. Being flexible is key.

Action plan examples

Here are a few examples of how the team have used action plans to reduce our UPF consumption.

To help us not buy UPF when we go shopping, my partner and I have set up a shared notes page on our phones to keep a constant shopping list using the tick list function. At home, whenever we run out of an ingredient, we untick it, and it goes to the top of the list. Then, when we go shopping we can easily see what we need to buy and what we already have at home. This saves us time and money, and means we don't get distracted by foods we don't want or need.

[redacted]

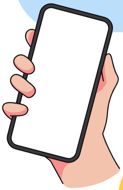

Planning is crucial for me because my work pattern is erratic.

My partner and I make time on a Sunday morning to write a meal plan for the week, thinking about when we both have time to cook and picking recipes that suit batch cooking to take into work.

[redacted]

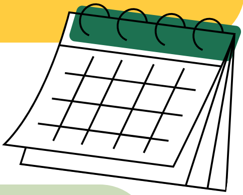

My family is 'flexitarian' and we used to eat a lot of meat substitutes, like veggie sausages and mince. To reduce UPF we wanted to set some sustainable, positive goals. Our initial goals were to 1) cook more from scratch as a family, 2) use more minimally processed ingredients, and 3) read food labels and become more aware of what's inside the package!

On our family WhatsApp group we share recipes, and each week one of us decides what we'll have – and we take it in turns to cook. Now this has become our 'new normal', we have started to set other, more challenging goals, like choosing less well-known vegetables (okra was our recent discovery), herbs, and spices. Being organised is key, and having lots of easy-to-use non-UPF ingredients at home helps us stick to our goals.

[redacted]

Goal Setting and Action Planning

Now it's time to put what you've learned about goal setting into practice by setting some of your own. Think about your 'why' and remember to be specific and realistic, and make sure your goals are relevant to **you**.

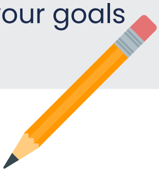

What is my goal for this month?

Why did I choose this goal?

I will start working towards my goal on this date:

Steps I will take towards my goal (action plan):

How confident I feel that I can do this 1=Not at all to 10=Very

Where I will go if I need help:

What I need to get started:

How will I measure my progress along the way?

....
....
....
....
....
....
....

Challenges I might face:

....
....
....
....
....
....

What I will do if I feel like quitting:

....
....
....
....
....
....

How will I know if I have achieved my goal?

....
....

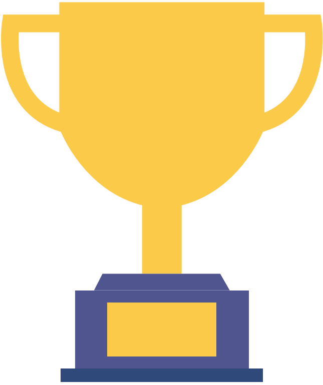

Goal tracking form : example

**Goal 1:** *e.g. I will take a pre-prepared lunch to work when I'm in UCLH*				
**Why it is important:** *e.g. I want to have a healthier diet and cut down UPF*				
**WEEK 1**	When do you **plan** to do this?	When **did** you do this?	What got in the way or what helped?	Did you troubleshoot?
Mon	✓	✓	batch cooked on Sun	
Tues	✓	✓	batch cooked on Sun	
Wed	✓		forgot my lunch!	Used online food mapper to choose a non-UPF option
Thur				
Fri				
Sat				
Sun				

Goal outcome map : example

Why did I take part in the trial?

I don't feel particularly healthy. I really want to improve my wellbeing through paying more attention to nutrition. I understand that UPF is contributing to these issues.

What do I see for myself in the future as a result of taking part in the UPDATE trial?

I feel healthier, pay more attention to my health and take it seriously

eat more unprocessed food

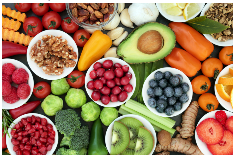

improve my gut health

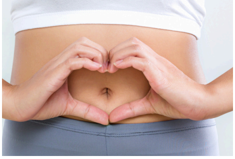

I am accountable

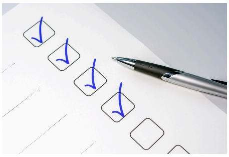

look at food labels

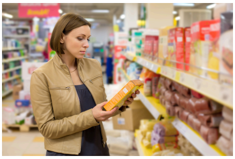

I am more confident and feel better in my skin (and clothes!)

Goal outcome map

Why did I take part in the trial?

What do I see for myself in the future as a result of taking part in the UPDATE trial?

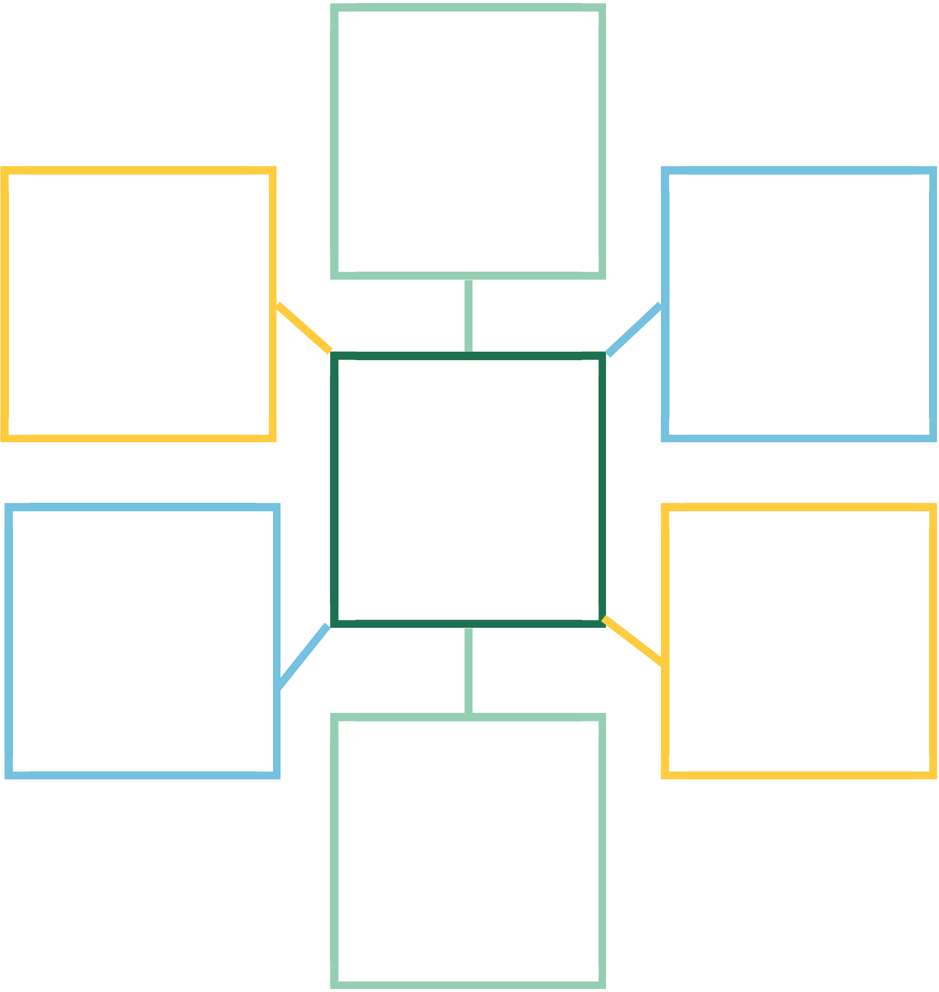

Notes and reflections

Snack and product ideas

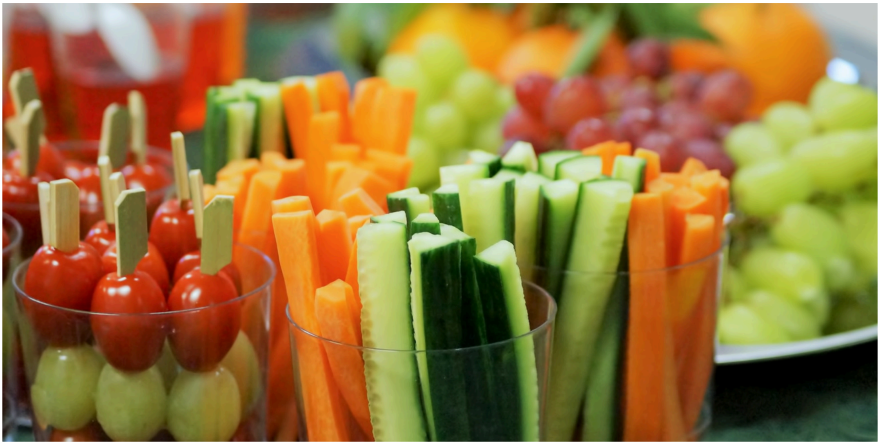

Here are some non-UPF snack ideas and some examples of non-UPF products to help you get started.

Snack ideas

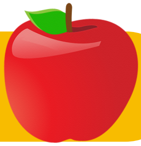

A piece of fresh fruit - you can pick up a banana for 10-20p in most shops

Small bowl of fruit salad - chop up your favourites. Add some natural yoghurt if you like.

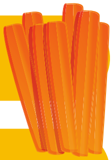

Carrot sticks and 2 tablepoons of houmous or guacamole

Veg sticks (e.g. carrot, cucumber, pepper) and cottage cheese

Unsalted nuts and seeds (e.g. a small palmful of monkey nuts, brazil nuts, or cashews)

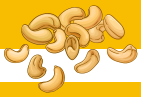

Apple and two teaspoon of unsweetened peanut butter (most supermarkets sell 100% peanut versions)

Small bowl of natural yogurt with fruit. Frozen berries and sliced banana work really well. Add a swirl of honey, cinnamon or chopped seeds on top if you like!

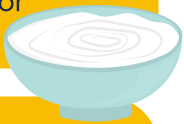

Two plain **crackers** (see page 31 for non-UPF options), a matchbox size block of **cheese** and cherry tomatoes (or other veg)

Two plain rice cakes with cream cheese (see page 31 for non-UPF cream cheeses)

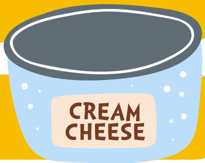

A mug of home made soup

What about ready-made products?

Ready-made products can be tricky, as many contain cosmetic additives to increase profit. However, we've done our research and found some non-UPF options of handy products like cream cheese, crackers and yogurt. **Check out our website for even more options!**

Product	Common UPF ingredients	Non-UPF examples	UPF examples
Muesli	Whey powder, flavouring	Sainsbury's Nutty Muesli, Dorset Cereals Simply Nutty and Simply Delicious Mueslis	Alpen, Dorset Cereals Simply Fruity Muesli
Canned coconut milk	Guar gum, carboxymethyl cellulose, sucrose esters of fatty acids	Tropical Sun 100% canned coconut milk, Biona, Clearspring	Tesco, Pride, Blue Dragon, Dunn's River, Kingfisher
Soy sauce	Caramelised sugar syrup, colours, flavour enhancers	Kikkoman Soy Sauce and Tamari	Tesco, Lee Kum Kee, Amoy, Asda
Yogurt	Gelling agent (pectin), milk protein, flavouring	Most natural and Greek yogurt containing only milk and live cultures	Flavoured yogurt (including fruit flavour), Activia
Cream cheese	Stabiliser (guar gum), citrus fibre	Most supermarket versions, including some garlic & herb varieties	Philadelphia (all varieties), Creamfields Soft Cheese, Dairylea
Crackers	Glucose-fructose syrup, flavourings	Matzo, most cream crackers	Flavoured crackers e.g. garlic flavour, Ritz
Nut butters	Hydrogenated vegetable oils, mono and diglycerides	Most supermarkets sell own-brand 100% nut versions, Pip and Nut, ManiLife, Meridian, Nuts about Nature	Skippy, Jif, KP, we recommend avoiding versions with added oil and sugar

Correct as of September 2023

Ready-made products

Product	Common UPF ingredients	Non-UPF examples	UPF examples
Pesto	Glucose syrup, pea fibre, bamboo fibre, antioxidants, preservatives, acidity regulators (lactic acid, glucono delta lactone)	Most fresh pesto found in the fridge in the supermarket	Most pesto in a jar
Quinoa, couscous & pulses	Maltodextrin, citric acid, glucose syrup, stabaliser (guar gum), spice extracts, herb extracts, colours	Merchant Gourmet Glorious Grains and Simply Cooked Red & White Quinoa, all plain grains that require boiling	Most pre-cooked pouches
Breads	Dextrose, preservatives, emulsifiers (mono- and diacetyl tartaric acid esters of mono- and diglycerides of fatty acids)	Jason's Sourdough Straight Up Great White, Superb Sprouted Grains, and Majestic Malted, Bertinet Bakery White Sourdough and Seeded Sourdough, Crosta & Moillica flatbreads, Fitzgeralds Round Wholemeal Pitta	All supermarket bread, including from the bakery section
Baked beans	Modified maize starch, maltodextrin, colours, spice extracts, flavourings	Heinz organic, Mr Organic plain, Clearspring, Biona tinned baked beans	Tesco, Waitrose, Branston, Heinz original, Weight Watchers
Mayonnaise	Stabilisers (xanthan gum, propylene glycol alginate, guar gum), preservative (potassium sorbate)	Dr. Will’s all natural classic mayo Hunter & Gather, Delouis, Hillfarm Garlic Mayonnaise, Waitrose Duchy	Heinz, Hellman’s, Stokes, supermarket own-brand
Chocolate	Emulsifier (soya lecithins), flavouring	Montezuma's 73%, Raw Halo 85% and 76%, Hu	Almost all other brands

Recipe Ideas

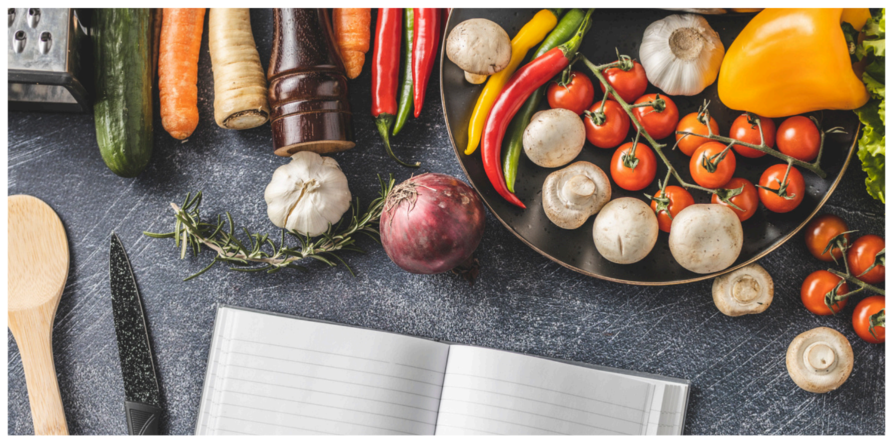

We asked our team to recommend a few of their favourite recipes, and have given some examples here. These are taken or adapted from cookbooks that we use, and we have linked to these on our website.

There are more recipes, cookbooks and other resources on our website: **findmempf.com**

Quick breakfast ideas

Boiled egg, asparagus

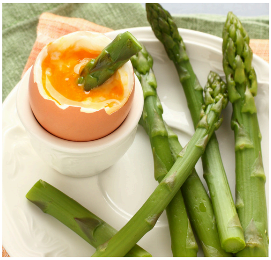

INGREDIENTS

4 eggs
8 asparagus spears

METHOD

1) Boil the kettle then fill a pan with boiling water.

2) Gently lower the eggs in, and cook for 5 minutes, 30 seconds (for a slightly runny yolk).

3) At the same time cook the asparagus for 3-5 mins in salted boiling water.

4) Drain the asparagus, season with salt/pepper.

5) Serve on plate with eggs.

PREP: 15 mins
SERVES: 2

Overnight oats

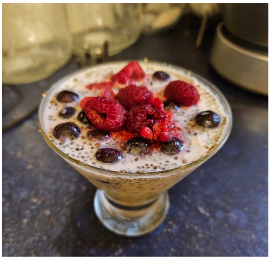

INGREDIENTS

80 g rolled oats
200 ml milk or water (or a mix)
4 tsp chia seeds
120 g frozen berries
1 tsp honey

METHOD

1) Get two small bowls, glasses or jam jars.

2) Add 40 g oats, 100 mls milk/water, and 2 tsp chia seeds to each of two bowls (or glasses/jam jars), stirring as you combine to remove any lumps.

3) Add half tsp of honey to each and stir.

4) Top with frozen berries and leave in the fridge overnight.

PREP: 5 mins
SERVES: 2

Buy large bags of frozen berries - they last far longer and are cheaper.

Lunch ideas

Egg & mango chutney flatbread

INGREDIENTS

100 g self-raising flour, plus extra for dusting
6 tbsp natural yoghurt
Dash of olive oil
4 large eggs
2 tbsp non-UPF mango chutney (I like the brand Geeta's)
1 fresh red chilli (chopped)

METHOD

1) In a bowl, mix the flour, a pinch of sea salt, 4 tbsp yoghurt and 1 tbsp olive oil until you have a dough.

2) Tip out onto a clean, floured surface, and stick together with your hands.

3) Split into two, and gently roll out into a flatbread shape.

4) Heat a dash of olive oil in a non-stick pan.

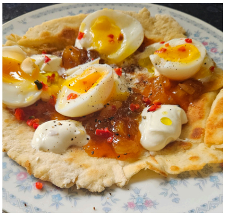

PREP: 12 mins
SERVES: 2

5) At the same time, put a pan of boiling water onto the heat and gently lower in the eggs - time for 5 minutes 30 seconds. Once done empty the pan and fill with cold water. Peel the eggs when cool enough to handle.

6) Cook flatbreads one at a time in the frying pan until golden brown, turning halfway (around 3 mins).

7) Put the flatbreads on plates, dollop with natural yogurt and mango chutney, peeled eggs (cut in two) then sprinkle with salt, pepper, olive oil and the chilli.

These flatbreads are so easy to make and you can top them with anything!

Veg, cheese and egg bake

INGREDIENTS

2 red or yellow peppers, chopped
4 spring onions, sliced
160g spinach, chopped
8 eggs
2 tsp harissa (optional)
120g ricotta cheese
80g feta cheese

METHOD

1) Preheat oven 200°C/180°C fan/Gas mark 6. Line baking tin with baking parchment.

2) Put the peppers, spring onions and spinach in a heat-proof bowl. Add 1 tbsp water and microwave for 3 mins. Use a colander to drain excess water.

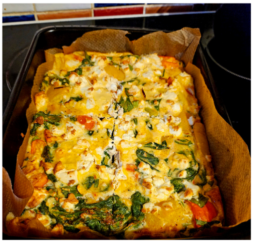

PREP: 10 min
COOK: 25
SERVES: 4

3) Stir eggs, harissa, and ricotta in a bowl to combine. Add salt and black pepper. Add the veg to this bowl and stir.

4) Add the mixture to the baking tin and crumble feta on top. Bake for 25 mins.

Perfect to make in advance and have as work lunches for with a side salad

Salmon, beetroot, aubergine salad

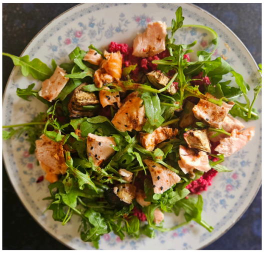

PREP: 15 mins
COOK: 30
SERVES: 2

INGREDIENTS

1 aubergine cut into 2cm pieces
1/2 tsp cumin seeds
1/2 small cauliflower, broken into florets
150g cooked beetroot, drained and chopped
3 tbsp full fat Greek-style yoghurt
1 tbsp cider vinegar
10g fresh mint, chopped
50g rocket
2 **cooked** salmon fillets
1/2 tsp ras el hanout (optional)
1 tsp mixed seeds (optional)
1/2 a lemon

METHOD

1) Preheat oven to 200°C/180°C fan/Gas mark 6.
2) Spread the aubergine on one side of a large baking tray and drizzle with oil. Add the cumin seeds, salt and pepper, mix together and bake for 10 mins.
3) Meanwhile, toss the cauliflower with remaining oil, salt and pepper.

4) Remove tray from oven, add cauliflower to the tray with the aubergine and bake for another 20 mins until veg is soft and browned.

5) Remove veg tray from the oven, cover the aubergine to keep warm. Transfer the cauliflower to a large bowl or jug, along with beetroot, yoghurt and vinegar. Blitz with a stick blender (or put the whole lot in a blender), stir in the mint (saving a few leaves to garnish).

6) Spread the cauliflower-beetroot mixture on 2 plates, top with the salmon, aubergine and rocket. Sprinkle with ras el hanout and seeds if using. Squeeze with lemon juice. Garnish with mint leaves.

Double this for 4 lunches. If your tray isn't big enough use separate trays for cauliflower and aubergine

Mackerel super salad

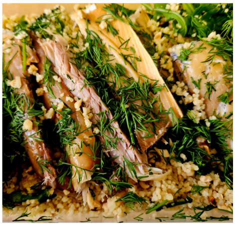

PREP: 15 min
COOK: 0
SERVES: 4

INGREDIENTS

200g dry coucous
2 1/2 tbsp olive oil
300 ml boiling water
1 tsp cumin
2 lemons
1/2 head of broccoli cut into florets
4 filets smoked mackerel
80g chopped toasted hazelnuts
75g dried apricots, chopped
2 tbsp toasted seeds
2 tbsp dill
2 handfuls of rocket
Salt and pepper

METHOD

1) Put dry couscous in a bowl. Add the oil, boiling water, and cumin, zest and juice of half a lemon, cover and leave for 10 min.

2) Cook the broccoli in boiling water for 3 min then run under tap until cold.

3) Break up the couscous, peel the skin off the mackerel and flake it in.

4) Mix in the nuts, apricots, broccoli, dill and rocket.

Dinner ideas

Jerk chicken drumsticks and slaw

INGREDIENTS
1-2 tsp ground cayenne pepper
2 tsp dried thyme
2 tsp dried parsley
1 tbsp ground paprika
1 veg stock cube, crumbled
1/2 tsp ground cinnamon
2cm fresh ginger
1/2 lemon, zested then sliced
2 tbsp olive oil
8 chicken thighs or drumsticks
SLAW
100g natural yoghurt
1 large handful parsley or coriander, chopped
1/2 lemon, juiced
2 tsp wholegrain mustard
1/2 white cabbage, chopped
5 spring onions, chopped

METHOD

1) Pre-heat oven to 200°C/180°C fan.

2) Mix cayenne pepper, thyme, parsley, paprika, stock cube, cinnamon, ginger and lemon zest in a bowl (jerk seasoning).

3) Put chicken in large baking dish, coat with 2 tbsp oil.

4) Rub jerk seasoning into chicken with hands or a wooden spoon, cover and marinate in fridge for at least 15 mins.

5)Take chicken out of fridge. Slice half of the lemon that you zested. Place lemon slices on top of the chicken, then bake for 45 mins.

6)To make slaw add yoghurt, parsley, juice of 1/2 lemon, mustard, salt and pepper in a bowl. Add carrots, cabbage and spring onion and mix well.

7) Check the chicken is cooked through and serve with generous helping of slaw.

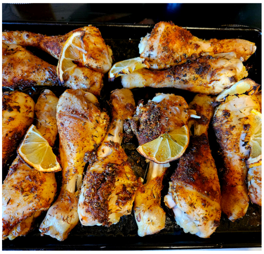

PREP: 15 mins
COOK: 45
SERVES: 4

Works really well cold the next day as a lunch

Mozzarella chicken

INGREDIENTS
4 tbsp olive oil
1 onion
2 garlic cloves peeled and sliced (optional)
2 x 400g tin chopped tomatoes
Small handful fresh basil
200 mls vegetable or chicken stock
4 x skinless chicken breast
2 x 60g balls of mozzarella sliced
Any salad or veg to serve on the side

METHOD

1) Pre-heat oven to 190°C/170°C Fan/Gas mark 5.

2) Put oil, onion, garlic (if using), in a frying pan over a medium heat for 5-8 mins, until onion softens.

3) Add chopped tomatoes, basil and stock and bring to the boil. Simmer for at least 5 mins (longer if possible), stirring occasionally.

4) Meanwhile place chicken breasts between two sheets of baking parchment and use a rolling pin/something heavy to pound and flatten a bit. Place chicken in ovenproof dish or baking tray, cover with the tomato sauce and place sliced mozzarella on top. Roast for about 20 mins, or until chicken is completely cooked through and cheese is melted and golden.

5) Serve with side salad or veg.

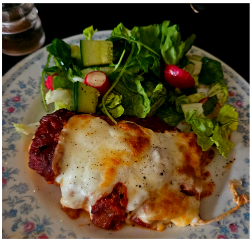

PREP: 10 min
COOK: 40 min
SERVES: 4

Try making some potato wedges to go with it!

Section 2: Physical activity

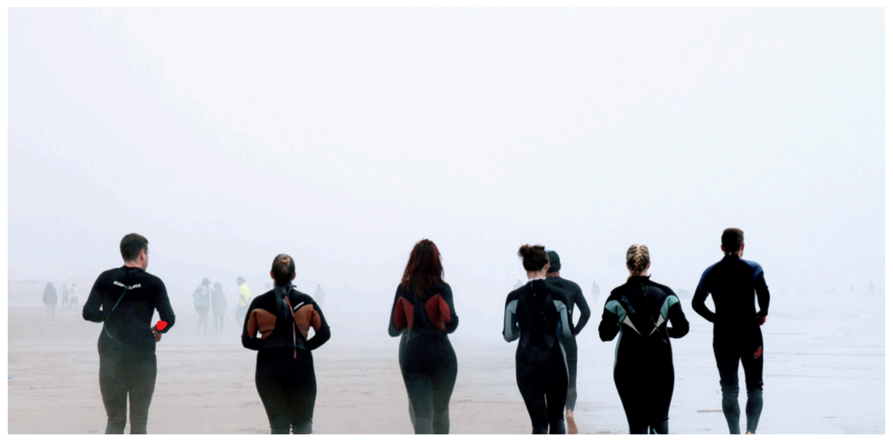

This section of the booklet is designed to be used with Behavioural call 3.

Physical activity and health

Decades of research provides very strong evidence that physical activity is important for both physical and mental health [8]. For example, people who are active have reduced risk of heart disease, some cancers and type 2 diabetes. Physical activity can also improve mental health and may reduce the risk of the cognitive decline as we age. Physical activity can also support our immune function and reduce our risk of infection.

In short, doing enough physical activity makes us **feel and function better,** and **reduces our risk of illness** both now and in the future. The term physical activity covers both exercise (which is structured), and the movement we accumulate throughout the day.

Most people know that physical activity is good for health, but still the majority of people struggle to be active enough. Translating that knowledge into action is not always easy. In the third behavioural call we will give you some evidence-based tools that might help.

If exercise were a pill, it would be one of the most cost-effective drugs ever invented!

Dr Nick Cavill, NHS health promotion consultant

Whilst we still have a lot to learn, there are some key things that are important to successfully increasing physical activity. Similarly to dietary change, these are **setting goals**, findings ways to **self-monitor** your behaviour [7], and establishing physical activity **habits**.

It is also important that the type of activity you choose is something you enjoy (or can learn to enjoy) and something that will be possible for you to integrate into your life in the long-term.

How much physical activity should I be doing?

The benefits of physical activity

- benefits health
- improves sleep
- maintains healthy weight
- manages stress
- improves quality of life

Reduces your chance of:

type 2 diabetes	cardiovascular disease	falls and depression	joint and back pain	colon and breast cancer
up to 40%	up to 35%	up to 30%	up to 25%	up to 20%

How much should I do?

Some is good, more is better → Make a start today it's never too late → Every minute counts

At least 150 minutes of moderate intensity per week
(increased breathing and able to talk)

Examples of moderate intensity activity are:
- swimming
- brisk walking
- cycling

OR

At least 75 minutes of vigorous intensity per week
(breathing fast and difficulty talking)

Examples of vigorous intensity activity are:
- running
- climbing stairs
- sport

or a combination of both

Build strength

To keep muscles, bones and joints strong, build strength on at least 2 days a week.

Examples of bone and muscle strengthening activities are:
- carrying heavy bags
- gym
- yoga

Balance activities for older adults

For older adults, to reduce the chance of frailty and falls, improve balance 2 days a week.

Examples of balance activities are:
- dancing
- bowls
- Tai Chi

UK Chief Medical Officer Physical Activity Guidelines, 2019

Goal outcome map

Why did I take part in the trial?

What do I see for myself in the future as a result of taking part in the UPDATE trial?

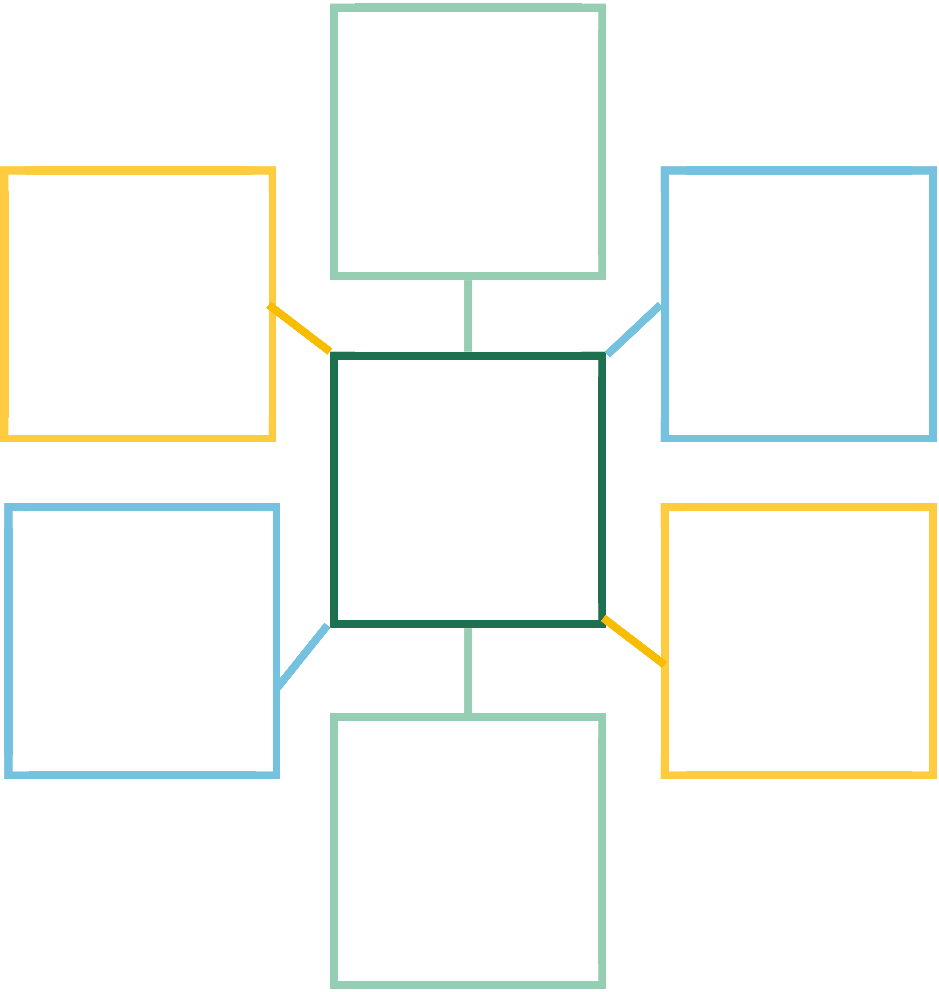

Goal Setting and Action Planning

Now it's time to put what you've learned about goal setting into practice by setting some of your own. Think about your 'why' and remember to be specific and realistic, and make sure your goals are relevant to **you**.

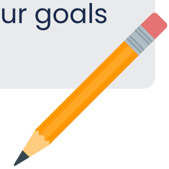

What is my physical activity goal for this month?

....

Why did I choose this goal?

....

....

I will start working towards my goal on this date:

....

Steps I will take towards my goal (action plan):	*How confident I feel that I can do this 1=Not at all to 10=Very*
....	....
....	....
....	....
....	....
....	....
....	....
....	....

Where I will go if I need help:

....

....

....

....

....

....

What I need to get started:

....

....

....

....

....

....

How will I measure my progress along the way?

Challenges I might face:

What I will do if I feel like quitting:

How will I know if I have achieved my goal?

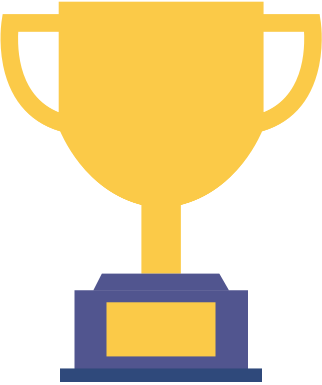

Goal tracking form : example

**Goal 1:** ***e.g. I will go for a brisk 30 min walk on days I work from home***				
**Why is it important:** ***e.g. I want to improve fitness and have time to myself***				
**WEEK 1**	**When and where** do you plan to do this?	Did you do this?	What got in the way or what helped?	Did you troubleshoot?
Mon				
Tues				
Wed				
Thur	8-8.30am local streets	✓	Used the active 10 app	N/A
Fri	8-8.30am local streets	✓	put my trainers by the front door as a reminder	N/A
Sat				

Space to summarise key points from call

REFERENCES

Here are a few of the key references we used to create this booklet. For a more extensive list of sources, including books, journal articles and documentaries, visit our website.

1. World Cancer Research Fund 'What is ultra-processed food and how should we be worried about it?' www.wcrf.org/what-is-ultra-processed-food-and-should-we-be-worried-about-it Accessed Sept. 2023
2. Hall *et al*. Ultra-Processed Diets Cause Excess Calorie Intake and Weight Gain: An Inpatient Randomized Controlled Trial of Ad Libitum Food Intake. Cell Metab. 2019;2;30;67-77.e3 .
3. Srour et al. Ultra-processed food intake and risk of cardiovascular disease: prospective cohort study (NutriNet-Santé) BMJ. 2019;365:l1451
4. Li *et al*. Association of Ultraprocessed Food Consumption With Risk of Dementia: A Prospective Cohort Study. Neurology. 2022; 99:e1056-e1066.
5. Nilson *et al*. Premature Deaths Attributable to the Consumption of Ultraprocessed Foods in Brazil. Am J Prev Med. 2023;64:129-136.
6. Bailey, *et al*. Goal setting and action planning for health behavior change. American J Lifestyle Med, 2019: 615-618
7. Samdal, *et al*. Effective behaviour change techniques for physical activity and healthy eating; systematic review and meta-regression analyses. Int J Behav Nutr Phys Act. 2017;14:42.
8. Warburton *et al*. Health benefits of physical activity: a systematic review of current systematic reviews. Curr Opin Cardiol. 2017; 32:541-556.

UPDATE

Contacts

If you need to book or rearrange any of your calls please email [redacted]

If you want to discuss any other part of the Behavioral Support programme please contact [redacted]

Don't forget to check out our website by scanning the QR code or visiting findmempf.com

UCL
