# Supplementary figures and images for: Development, content and planned evaluation of a behavioural support intervention to reduce ultraprocessed food intake and increase physical activity in UK healthcare workers: UPDATE trial stage 2 study protocol
Source: BMJ Open. 2025 Oct 29;15(10):e107435. doi: 10.1136/bmjopen-2025-107435 (PMC12574385; doi:10.1136/bmjopen-2025-107435)

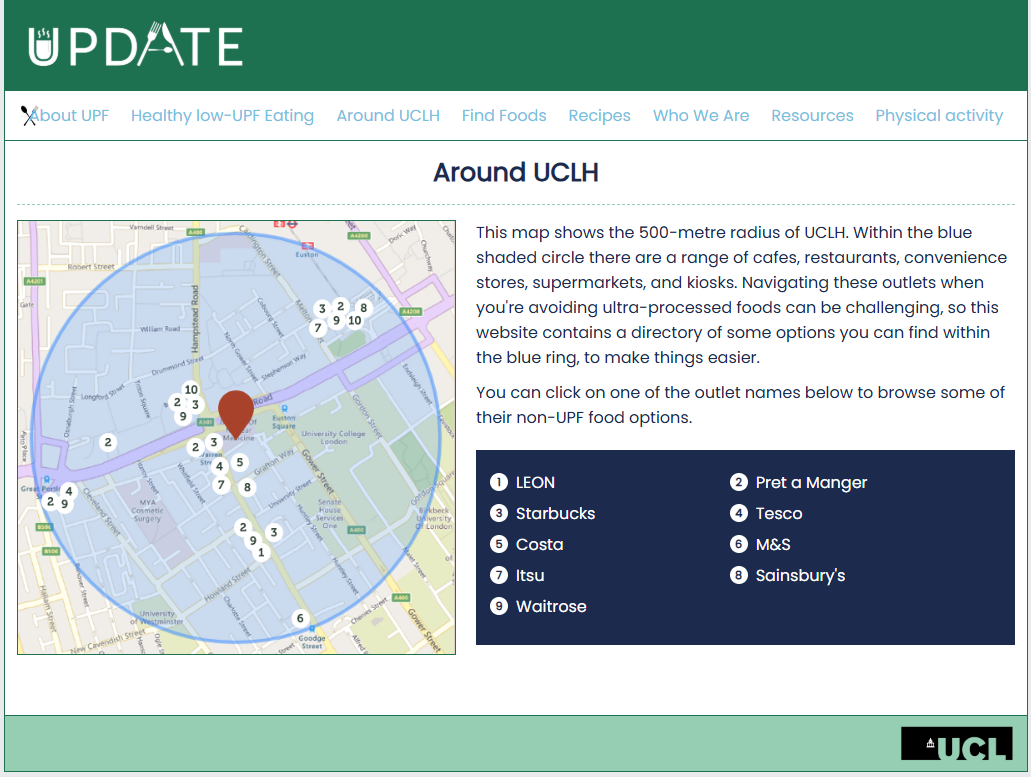


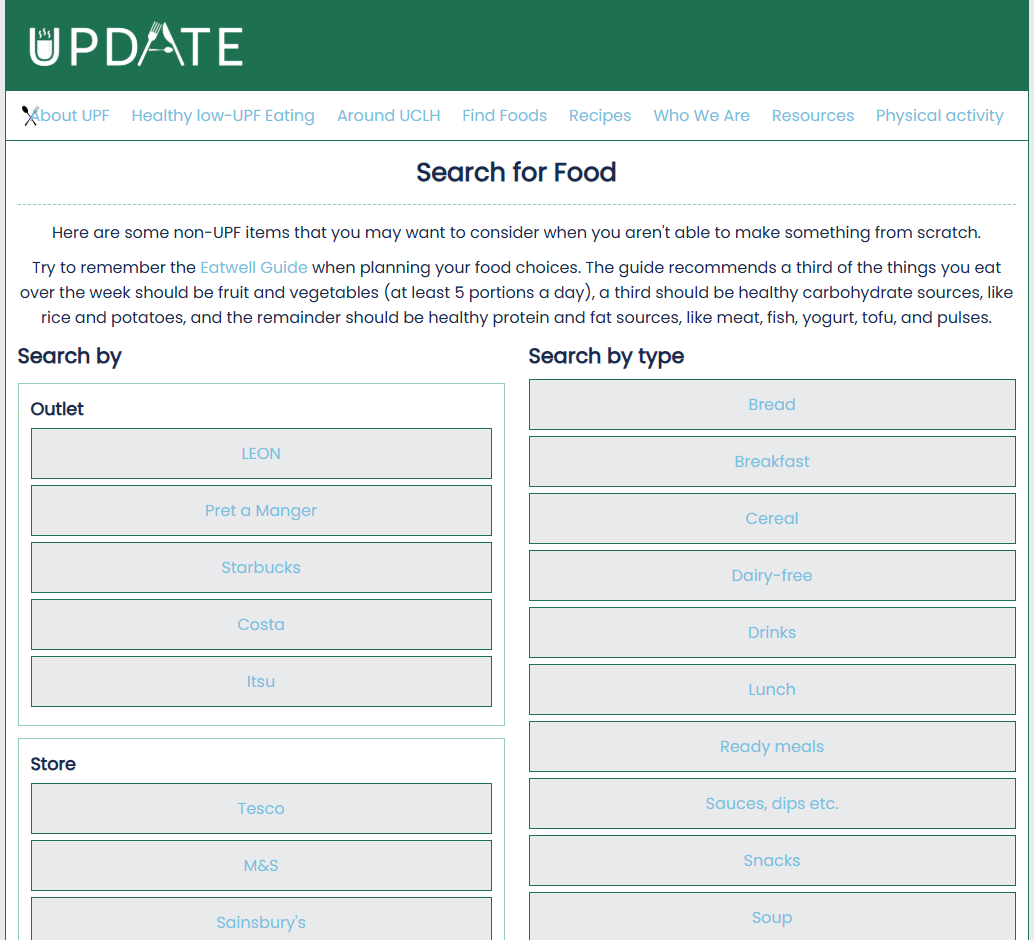


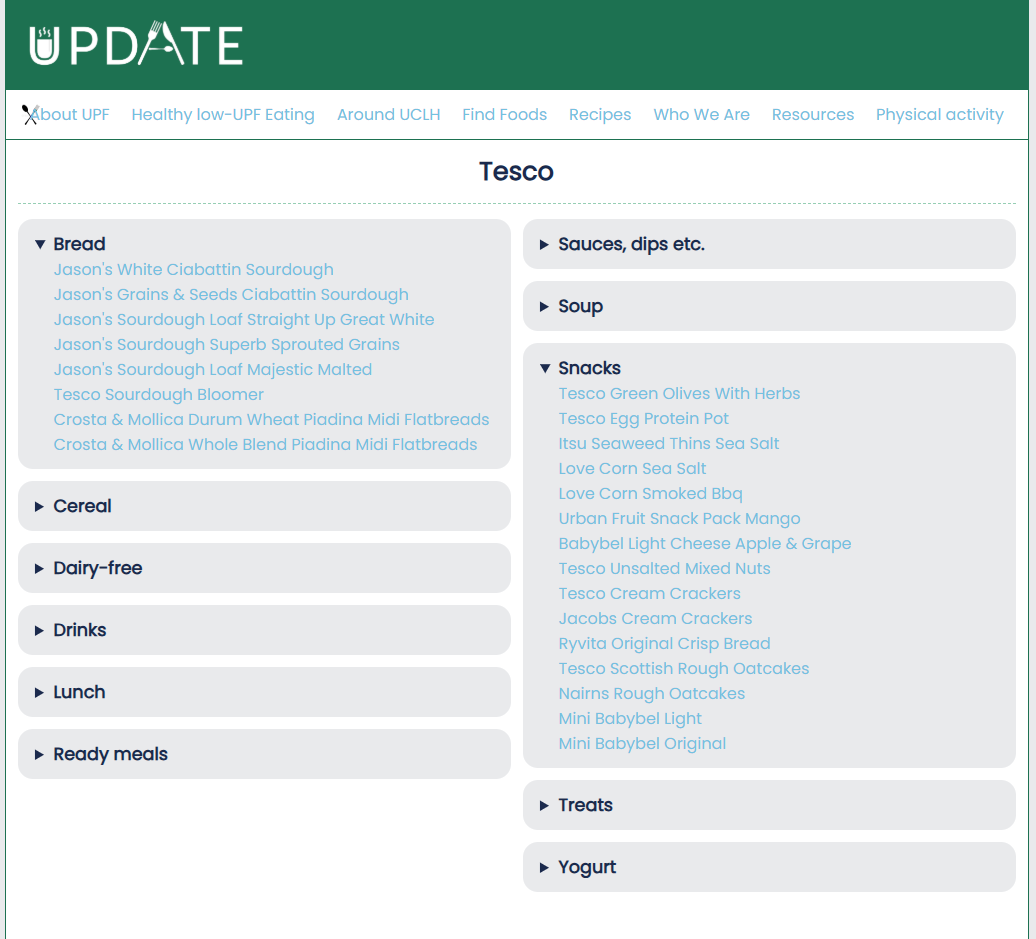


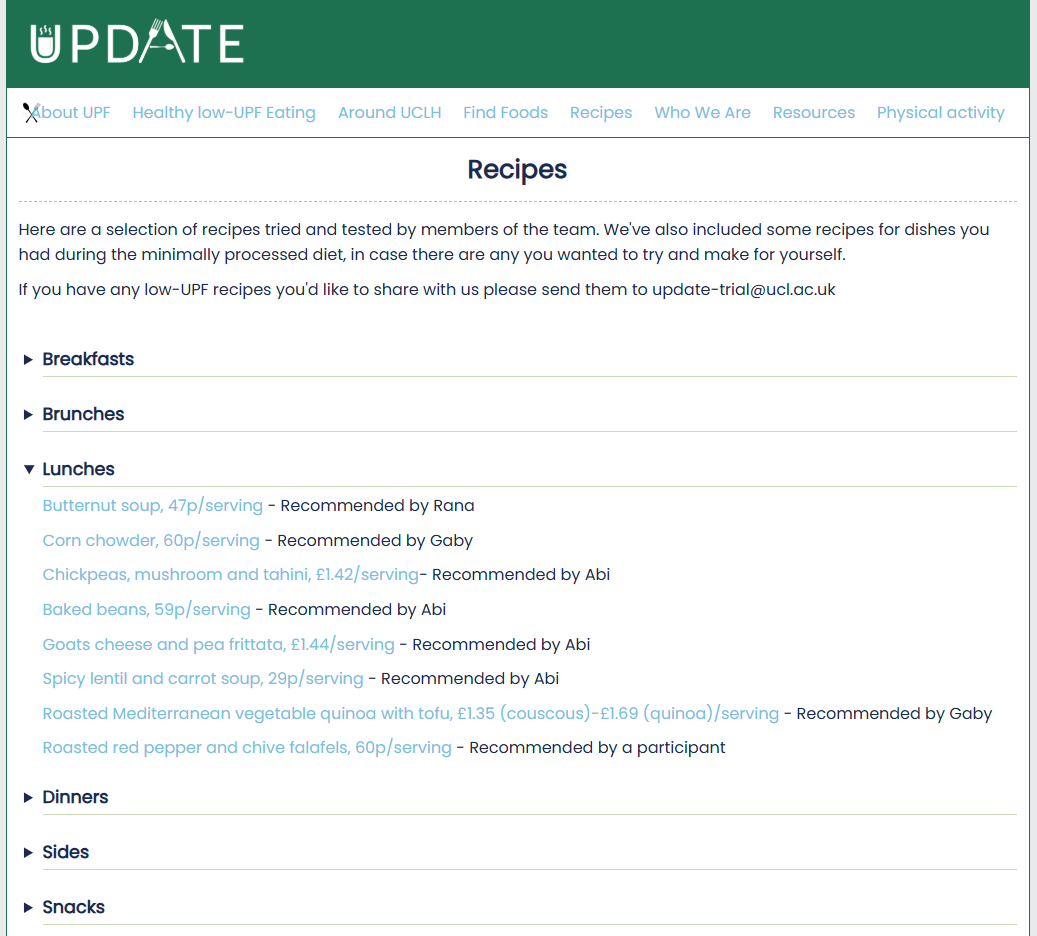

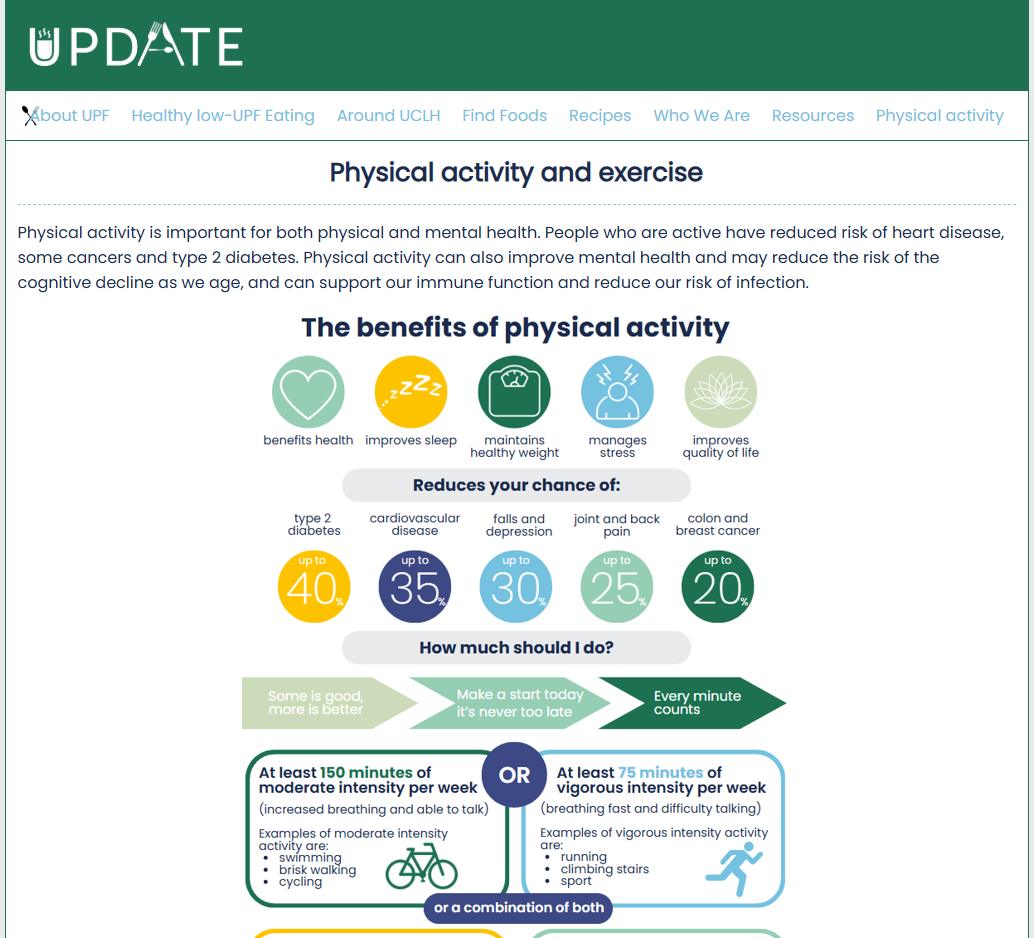


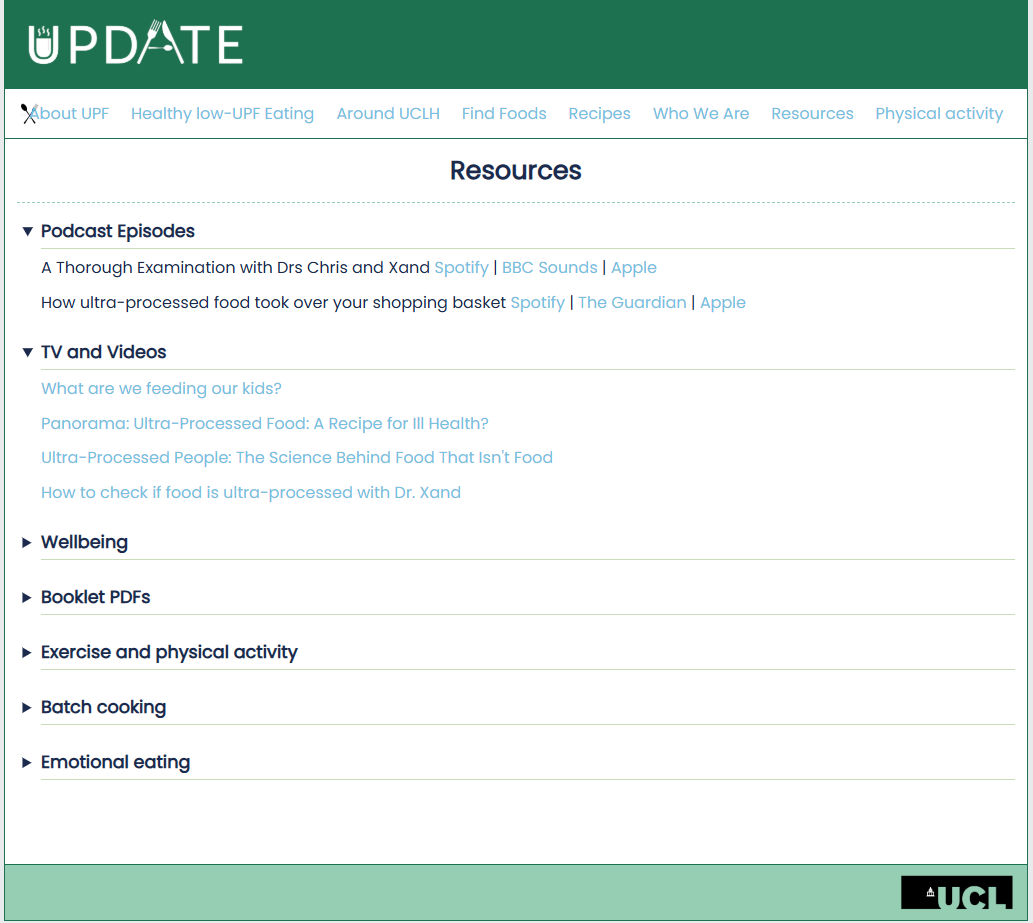

Supplement: online supplemental file 4 [file bmjopen-15-10-s004.docx]
