# Supplementary material for: Development, content and planned evaluation of a behavioural support intervention to reduce ultraprocessed food intake and increase physical activity in UK healthcare workers: UPDATE trial stage 2 study protocol
Source: BMJ Open. 2025 Oct 29;15(10):e107435. doi: 10.1136/bmjopen-2025-107435 (PMC12574385; doi:10.1136/bmjopen-2025-107435)
Supplement: online supplemental file 5 [file bmjopen-15-10-s005.docx]

Table 1 Adapted Dreyfus rating scale for the intervention BCT calls^1^

| **Scoring** | **Examples** | **Delivery Fidelity Category** |
| --- | --- | --- |
| 0 | Absence of feature and/ or highly inappropriate performance | Low fidelity |
| 1 | Minimal use of feature and/ or inappropriate performance | Low fidelity |
| 2 | Scope for improvement, alongside numerous minor and some major inconsistencies | Scope for improvement |
| 3 | Competent, good features but some minor inconsistencies or problems | Competent |
| 4 | Very good features, few inconsistencies or problems | Proficient |
| 5 | Excellent features, no problems or inconsistencies | Expert |

1. Cross R, Greaves CJ, Withall J, Rejeski WJ, Stathi A. Delivery fidelity of the REACT (REtirement in ACTion) physical activity and behaviour maintenance intervention for community dwelling older people with mobility limitations. *BMC Public Health*. Jun 3 2022;22(1):1112. doi:10.1186/s12889-022-13496-z
