# Supplementary material for: Development, content and planned evaluation of a behavioural support intervention to reduce ultraprocessed food intake and increase physical activity in UK healthcare workers: UPDATE trial stage 2 study protocol
Source: BMJ Open. 2025 Oct 29;15(10):e107435. doi: 10.1136/bmjopen-2025-107435 (PMC12574385; doi:10.1136/bmjopen-2025-107435)
Supplement: Supplementary file 6 [file bmjopen-2025-107435.reviewer_comments.pdf]

PEER REVIEW HISTORY

BMJ Open publishes all reviews undertaken for accepted manuscripts. Reviewers are asked to complete a checklist review form and are provided with free text boxes to elaborate on their assessment. These free text comments are reproduced below.

ARTICLE DETAILS

Title (Provisional)

Development, content and planned evaluation of a behavioural support intervention to reduce ultra-processed food intake and increase physical activity in UK healthcare workers: UPDATE trial Stage 2 study protocol

Authors

Heuchan, Gaby Niamh; Buck, Caroline; Conway, Rana; Dicken, Samuel; Van Tulleken, Chris; Brown, Adrian Carl; Jassil, Friedrich C.; Makaronidis, Janine; Gandini Wheeler-kingshott, Claudia Angela; Batterham, Rachel; Fisher, Abigail

---

VERSION 1 - REVIEW

**Reviewer**	**1**
**Name**	**Alon, Kaplan**
**Affiliation**	**Sheba Medical Center**
**Date**	**06-Aug-2025**
**COI**	**None**

This is a very interesting protocol of a crossover randomized controlled trial (RCT) aimed at assessing an intervention designed to reduce ultra-processed food (UPF) consumption through behavioral change. Below are several comments and suggestions for improvement:

Relevance of the Introduction: The introduction primarily discusses obesity. However, this study does not appear to be focused on weight loss as a primary outcome. I recommend revising the introduction to better align with the study’s central aim—reducing UPF consumption through behavioral modification—rather than emphasizing obesity.

Focus on the UK Population: While the study targets the UK population, this context does not differ significantly from other Western countries in terms of UPF consumption patterns. Therefore, emphasizing the UK-specific context may not be essential, unless there is a clear rationale or unique aspect that justifies this focus.

Individual Tailoring: The study's goal of assessing individually tailored interventions and responses is commendable and ideal. However, this approach may present challenges in

terms of scalability and real-world effectiveness. It would be beneficial to address these potential limitations in the discussion.

Behavioral Support Delivery: Providing one-on-one behavioral support from the same behavioral scientist across participants may introduce bias. The success or failure of the intervention could be strongly influenced by the interpersonal relationship developed, which limits generalizability and scalability. I suggest randomizing the behavioral support provider to minimize this potential bias.

Mode of Intervention Delivery: If feasible, the study could examine the impact of the mode of behavioral sessions (e.g., in-person vs. video call). This would provide useful insights into the flexibility and practicality of delivering the intervention in different settings.

Physical Activity Guidance: I recommend incorporating guidance and education regarding physical activity earlier in the intervention. This may support a more holistic approach to lifestyle improvement and complement the dietary goals.

Washout Period: The washout period between weeks 11 and 14 is important for the crossover design. However, results from this period should be interpreted with caution, as participants may have already adopted lasting behavioral changes that carry over into the subsequent phase.

Statistical Analysis Plan: It is important that the authors explicitly outline the statistical analysis plan for the second stage of the study. Given the complexity introduced by the crossover design and potential carry-over effects, a detailed and transparent plan is essential to ensure the validity and interpretability of the results.

VERSION 1 - AUTHOR RESPONSE

Reviewer 1: Dr Kaplan Alon

Comment: The introduction primarily discusses obesity. However, this study does not appear to be focused on weight loss as a primary outcome. I recommend revising the introduction to better align with the study's central aim - reducing UPF consumption through behavioural modification - rather than emphasizing obesity.

Response: We thank the reviewer for this helpful observation. We have revised the Introduction (paragraphs 3–4) to better align with the study's primary aim of reducing UPF intake through behavioural modification. Specifically, we have:

1) Reduced emphasis on obesity and weight change as outcomes, and instead highlighted the broader evidence linking UPF intake to a range of health outcomes (including metabolic, cardiometabolic, and dietary health)

2) Added reference to recent trials in diverse populations and settings where the focus was on reducing UPF intake for dietary quality and broader health benefits, not solely weight loss (new references 28–30).

3) Revised the narrative to position UPF reduction itself as the central behavioural target, while still acknowledging obesity as one of several potential outcomes.

We believe these changes strengthen the alignment between the introduction and the study's central focus.

Comment: Focus on the UK Population: While the study targets the UK population, this context does not differ significantly from other Western countries in terms of UPF consumption patterns. Therefore, emphasizing the UK-specific context may not be essential, unless there is a clear rationale or unique aspect that justifies this focus.

Response: We agree and have reduced emphasis on the UK-specific focus, except where directly relevant (i.e. healthcare worker context). For example, we removed *"Two further pilot pre-post studies in adults have been conducted in the US,(25, 26) where UPF consumption levels align more closely with those in the UK than in Brazil, potentially making their findings more comparable.(27)"* (from the 3rd paragraph of the introduction) and instead stated that there is a need in general for more interventions to reduce UPF intake across diverse settings/populations.

Comment: Individual tailoring: The study's goal of assessing individually tailored interventions and responses is commendable and ideal. However, this approach may present challenges in terms of scalability and real-world effectiveness. It would be beneficial to address these potential limitations in the discussion.

Response: We acknowledge this limitation and have added a statement recognising scalability challenges and the potential for automation of tailoring (e.g. COM-B coding, digital tools, AI-assisted tailoring) in the manuscript (page 12, para 2).

Comment: Behavioural Support Delivery: Providing one-on-one behavioral support from the same behavioral scientist across participants may introduce bias. The success or failure of the intervention could be strongly influenced by the interpersonal relationship developed, which limits generalizability and scalability. I suggest randomizing the behavioral support provider to minimize this potential bias.

Response: We appreciate this concern. For this developmental stage of the study, consistency of provider was prioritised for fidelity and feasibility. We were also limited by budget at this stage of the development process, which meant we had one behavioural scientist to deliver the intervention; however others did join the team later on and took on several participants. In a future efficacy trial we would randomly allocate.

Comment: Mode of Intervention Delivery: If feasible, the study could examine the impact of the mode of behavioral sessions (e.g., in-person vs. video call). This would provide useful insights into the flexibility and practicality of delivering the intervention in different settings.

Response: We agree this is important. However, due to the vast majority of participants expressing a desire for remote delivery due to work schedules, we have little data on this. It is something we would look at and encourage delivery of the intervention in varied settings in a future trial.

Comment: Physical Activity Guidance: I recommend incorporating guidance and education regarding physical activity earlier in the intervention. This may support a more holistic approach to lifestyle improvement and complement the dietary goals.

Response: We thank the reviewer for this suggestion. In designing the intervention, we considered the sequencing of content carefully. The introductory sessions were deliberately focused on UPF education, NOVA classification, goal setting, and familiarisation with the programme resources (booklets, website, tracking tools), and they were information-dense. Evidence from health behaviour change research suggests that introducing too many changes simultaneously can reduce engagement and intervention effectiveness (e.g., Baxter et al., 2025), so we felt that adding physical activity at this early stage risked cognitive overload and goal dilution, potentially reducing engagement and adherence. That is why we staggered the introduction of physical activity.

Comment: Washout Period: The washout period between weeks 11 and 14 is important for the crossover design. However, results from this period should be interpreted with caution, as participants may have already adopted lasting behavioral changes that carry over into the subsequent phase.

Response: We recognise that our original wording may not have made this sufficiently clear. To address this, we have clarified throughout the manuscript that the washout period applied only to Stage 1 (the crossover feeding trial) and not to Stage 2 (the behavioural support intervention reported in this paper). All participants completed Stage 1, including its washout period, before beginning Stage 2. We have amended the abstract, introduction, and "Exploratory evaluation: planned analyses" sections to make this distinction explicit.

We also acknowledge that experiences from Stage 1 could potentially have influenced participants' engagement with Stage 2, and we have added a statement to note this possibility. However, as Stage 2 was designed to explore feasibility, acceptability, and retention rather than provide powered estimates of intervention effects, any such carryover is not expected to compromise the evaluation aims. To provide a clearer test of efficacy, future pilot RCTs will recruit participants who have not taken part in Stage 1.

Comment: Statistical Analysis Plan: It is important that the authors explicitly outline the statistical analysis plan for the second stage of the study. Given the complexity introduced by the crossover design and potential carry-over effects, a detailed and transparent plan is essential to ensure the validity and interpretability of the results.

Response: We thank the reviewer for raising this point. To clarify, the crossover design and associated statistical analysis plan related to Stage 1 of UPDATE, which has been published previously (Dicken et al., 2025).

Stage 2 - described in the present manuscript - was a 6-month behavioural support programme that followed completion of Stage 1 and did not employ a crossover design. As noted in the manuscript, Stage 2 was not designed or powered to test efficacy, but rather to provide exploratory data on feasibility, uptake, retention, and acceptability. The planned analyses therefore consist of descriptive statistics on these feasibility and acceptability outcomes, with any pre-post changes considered exploratory only. We have revised the text in the Exploratory evaluation: planned analyses section to make this clearer (p16, paragraph 2), and now explicitly cite the published Stage 1 paper for readers seeking details of the crossover design and associated statistical analysis.

VERSION 2 - REVIEW

**Reviewer**	**1**
**Name**	**Alon, Kaplan**
**Affiliation**	**Sheba Medical Center**
**Date**	**10-Sep-2025**
**COI**	

The authors have provided adequate and satisfactory responses to all of my previous comments and concerns.
